# Brazilian recommendations of mechanical ventilation 2013. Part 2

**DOI:** 10.1590/S1806-37132014000500003

**Published:** 2014

**Keywords:** Recommendations, Mechanical Ventilation, Respiratory Insufficiency

## Abstract

Perspectives on invasive and noninvasive ventilatory support for critically ill patients are evolving, as much evidence indicates that ventilation may have positive effects on patient survival and the quality of the care provided in intensive care units in Brazil. For those reasons, the Brazilian Association of Intensive Care Medicine (*Associação de Medicina Intensiva Brasileira* - AMIB) and the Brazilian Thoracic Society (*Sociedade Brasileira de Pneumologia e Tisiologia* - SBPT), represented by the Mechanical Ventilation Committee and the Commission of Intensive Therapy, respectively, decided to review the literature and draft recommendations for mechanical ventilation with the goal of creating a document for bedside guidance as to the best practices on mechanical ventilation available to their members. The document was based on the available evidence regarding 29 subtopics selected as the most relevant for the subject of interest. The project was developed in several stages, during which the selected topics were distributed among experts recommended by both societies with recent publications on the subject of interest and/or significant teaching and research activity in the field of mechanical ventilation in Brazil. The experts were divided into pairs that were charged with performing a thorough review of the international literature on each topic. All the experts met at the Forum on Mechanical Ventilation, which was held at the headquarters of AMIB in São Paulo on August 3 and 4, 2013, to collaboratively draft the final text corresponding to each sub-topic, which was presented to, appraised, discussed and approved in a plenary session that included all 58 participants and aimed to create the final document.

The present recommendations are a joint initiative of the Mechanical Ventilation Committee of the Brazilian Intensive Care Medicine Association (Associação de Medicina Intensiva Brasileira - AMIB) and the Commission of Intensive Therapy of the Brazilian Thoracic Society (Sociedade Brasileira de Pneumologia e Tisiologia - SBPT).

## Introduction

Invasive or non-invasive mechanical ventilation (MV) must be performed in an adequate and safe manner to avoid the occurrence of ventilation-induced lung injury. Based on physiological principles, evidence collected in laboratory experiments, and randomized clinical or observational studies involving actual patients that were available in the literature, current MV recommendations indicate that ventilatory support should be performed at a tidal volume (Vt) of 6 mL/kg predicted body weight, with a delta between plateau pressure and positive end-expiratory pressure (PEEP) not greater than 15 cmH_2_O, and end-expiratory pressure levels sufficient to avoid airway and alveolar collapse and ensure adequate gas exchange. Other recommendations include positioning the patient to guarantee adequate and harmless ventilation (such as prone positioning in cases of severe acute respiratory distress syndrome - ARDS) and the use of advanced support techniques (such as extracorporeal carbon dioxide (CO_2_) removal) in cases of refractory ARDS. The development of increasingly more sophisticated ventilators allow for fine adjustment of sensitivity and include several trigger mechanisms, different inspiratory flow speeds, acceleration, mechanisms for ending inspiratory time, and monitoring options, which enable adjustment of the patient-ventilator synchrony and MV as a function of the patient's disease. In this regard, the possibility of providing differential ventilatory support for restrictive and obstructive conditions stands out.

For that reason, joint analysis of the available evidence on ventilatory support by Brazilian experts who deal with mechanical ventilation like anesthesiologists, intensivists, pulmonologists, physical therapists, nurses, nutritionists and speech therapists was necessary. Such evidence, taken together with experience gathered by the various specialties, may provide guidance to health care professionals in Brazilian intensive care units (ICU) on how to provide safe and effective respiratory support for patients with respiratory failure, based on the best evidence available, in order to avoid the occurrence of ventilator-associated lung injury.

Therefore, the aim of the present study was to review the available literature on 29 subtopics related to ventilatory support for individuals with respiratory failure, and following presentation, discussion, and approval at a plenary session including all 58 participating specialists, to present the results in the form of recommendations and suggestions.

## Methods

Literature available from Medline (2003-2013) and the Cochrane Central Register of Controlled Trials (CENTRAL) was reviewed by specialists with a higher education (intensivists, anesthetists, pulmonary specialists, physical therapists, and nurses) who were distributed in pairs for review of each of the 29 selected subtopics related to non-invasive and invasive ventilatory support for patients with respiratory failure.

After reviewing the articles available in the literature, each pair answered the questions formulated by the organizing commission (composed by Carmen Silvia Valente Barbas, President of the Committee of Respiratory Failure and Mechanical Ventilation of AMIB, Alexandre Marini Isola, National Coordinator of the Course of MV in ICU - VENUTI, and Augusto Manoel de Carvalho Farias, Coordinator of the Department of Intensive Care of the SBPT) according to criteria previously suggested by other authors.^(^ 1 ^-^ 4 ^)^ Thus, the term recommendation was used when the level of evidence was high, i.e., derived from randomized studies conducted with more than 100 participants, meta-analyses, all-or-nothing effect, or patient safety. The term suggestion was used when the available evidence was weak, i.e., based on observational or case-control studies, case series, or on the experience of specialists to provide guidance for efficient and safe ventilatory support in Brazil. We therefore hoped that these evidence-based recommendations would help to avoid potential deleterious effects associated with inadequate ventilatory support in our patients.

The 58 participating specialists were requested to answer the proposed questions during an eight-hour session conducted at the Brazilian Intensive Care Medicine Association (Associação de Medicina Intensiva Brasileira - AMIB) on August 3, 2013. The answers were formulated based on the evidence available in the literature and on the experience of the specialists and were then presented at a plenary session that included all 58 participating specialists, which was held on August 4, 2013 at AMIB headquarters. During that session, the answers were discussed, modified when needed, voted on, and approved in accordance with the suggestions and observations of the specialists who attended the meeting.

The reports made by all the pairs of specialists were gathered by the project organizing commission, which revised, formatted and drafted the final document, following the authors' revisions. The document was then printed in the form of a bedside manual of recommendations to be distributed to ICUs all across Brazil, and it was also sent for publication in the Brazilian Journal of Intensive Care (Revista Brasileira de Terapia Intensiva - RBTI) and the Brazilian Journal of Pulmonology (Jornal Brasileiro de Pneumologia).

## Mechanical ventilation in chest trauma

### Noninvasive mechanical ventilation

### **Recommendation -** Noninvasive ventilation (NIV) is contraindicated in patients with upper airway injury, in the presence of hemodynamic instability, and in severe craniocerebral trauma.^(^ 5 ^-^ 10 ^)^

**Recommendation -** In patients with isolated chest trauma, early application of NIV can improve gas exchange, prevent orotracheal intubation (OTI), and reduce complications and ICU length of stay.^(^ 5 ^-^ 10 ^)^

**Recommendation -** The use of NIV should be monitored at the bedside by a healthcare professional within 30 minutes to 2 hours. For NIV to be considered successful, the following criteria should be met: reduction of the respiratory rate (f), increase in the tidal volume (Vt), improvement of the level of consciousness, reduction or cessation of the use of accessory muscles, increase in the partial pressure of oxygen (PaO_2_) and/or the peripheral oxygen saturation (SpO_2_), and reduction of the partial pressure of carbon dioxide (PaCO_2_) without significant abdominal distension. In unsuccessful cases, OTI and invasive MV should be performed immediately.

### Invasive mechanical ventilation(11)

### **Recommendation -** Patients with severe chest trauma, respiratory failure, and specific contraindications to NIV should be promptly intubated and ventilated.

### **Recommendation -** Initially, use an assist-control mode of ventilation, i.e., volume-cycled ventilation (VCV) or pressure-control ventilation (PCV), in chest trauma with severe respiratory failure. 

**Recommendation -** Regardless of the mode selected (VCV or PCV), patients with chest trauma should be initially ventilated with a Vt of 6 mL/kg predicted body weight, an f of 16-20 breaths/min, and a fraction of inspired oxygen (FiO_2_) that is sufficient to maintain an SpO_2 _> 92% and a PEEP of 5-10 cmH_2_O. In cases of ARDS, follow the instructions in the related section of the present Recommendations.

**Recommendation -** In cases of high output bronchopleural fistula, use the PCV mode, which will compensate for the leak. Another option is the use of high frequency oscillatory ventilation, only in centers with this capability and specialized personnel. In cases that are more severe, asynchronous independent lung ventilation can be either used or not, and the lung with the fistula is ventilated in the PCV mode with a distending pressure of < 15 cmH_2_O and low PEEP levels (< 10cmH_2_O).

### Pain control

**Suggestion - **Thoracic epidural analgesia, as part of a multimodal strategy, is recommended. If epidural analgesia cannot be used or is contraindicated, patient-controlled i.v. analgesia or intercostal nerve blockade can be used.^(^ 11 ^)^

### **Suggestion - **Administration of intermittent analgesia can be used in cases of less severe pain.

## Mechanical ventilation during surgical procedures

**Comment -** Postoperative pulmonary complications (PPC) contribute to a substantial proportion of the risks associated with surgery and anesthesia, being the major cause of morbidity and mortality in the postoperative period.^(^ 12 ^)^ PPC, which include respiratory infections, respiratory failure, pleural effusion, atelectasis, pneumothorax, bronchospasm, and aspiration pneumonitis, affect approximately 5% of patients undergoing surgery.^(^ 12 ^,^ 13 ^)^

### Specific care before intubation

Preoperative risk stratification

**Recommendation -** All patients should be assessed for risk of PPC by using a specific scale. The American Society of Anesthesiology (ASA) classification is a scale that is subjective and imprecise. Among the scales suggested for stratifying patient risk for PPC are the European Surgical Outcomes Study (EuSOS) scoring system and the Assess Respiratory Risk in Surgical Patients in Catalonia (ARISCAT).^(^ 13 ^)^

Pre-oxygenation during induction of anesthesia

### **Recommendation -** Pre-oxygenation with an FiO_2 _of approximately 100% for all patients aims at increasing reserve and reducing the risk of hypoxemia.^(^ 14 ^)^

Use of noninvasive ventilation during induction of anesthesia

**Suggestion - **Use NIV during induction of anesthesia for elective surgery in patients with decreased abdominal compliance and in those in whom it is necessary to use an FiO_2_ of 100% for pre-oxygenation. The use of NIV can prevent the development of atelectasis in patients with decreased abdominal compliance and in those undergoing pre-oxygenation with an FiO_2_ of 100%.^(^ 14 ^)^

Use of positive end-expiratory pressure and alveolar recruitment maneuvers during induction of anesthesia

### **Suggestion - **Use recruitment maneuvers and a PEEP of approximately 5-8 cmH_2_O after induction of anesthesia in patients with decreased abdominal compliance or in those who develop hypoxemia.^(^ 14 ^)^

### Specific care during the intraoperative period

Mode of ventilation

**Suggestion - **Use PCV to improve pulmonary mechanics in patients undergoing video-assisted laparoscopic surgery. Observe appropriate expired Vt values.^(^ 15 ^,^ 16 ^)^ In the remaining scenarios, there is no benefit of a mode of ventilation over another, provided that respiratory mechanics are respected.

Tidal volume

**Recommendation -** The use of MV with a Vt of 6 mL/kg predicted body weight should be considered in all patients. Various studies in various scenarios (abdominal, thoracic, and cardiac surgery) have demonstrated the benefit of using low Vt during surgery.^(^ 17 ^-^ 19 ^)^

Positive end-expiratory pressure

**Suggestion - **The use of a PEEP of approximately 5-8 cmH_2_O should be considered. All studies assessing conventional MV strategies versus protective strategies in patients undergoing surgery considered using a low Vt and a high PEEP in the protective strategies. In general, the use of a high PEEP results in better oxygenation and a lower incidence of PPC.^(^ 17 ^,^ 20 ^)^

Alveolar recruitment maneuvers

**Suggestion - **Use alveolar recruitment maneuvers (RM) during the intraoperative period to reverse alveolar collapse and improve oxygenation. The use of RM, in association with maintenance of high PEEP levels, reduces the amount of collapsed lung and improves patient oxygenation during surgery.^(^ 17 ^,^ 20 ^)^ Among the RM most often cited in the literature is maximum recruitment strategy (MRS), which seeks to maintain a high plateau pressure (Pplat) in the airway (approximately 40-45 cmH_2_O) for 60 seconds (as described in Part 1 of the present Recommendations).

Fraction of inspired oxygen

**Suggestion - **The lowest FiO_2_ level that can maintain SpO_2_ at approximately 96-98% should be used. The combination of an FiO_2_ of approximately 30-40% and high PEEP levels can maintain adequate oxygenation and reduce lung atelectasis. The role of high levels of oxygen in preventing surgical wound infection remains controversial.^(^ 14 ^)^

Respiratory rate

**Recommendation -** Use f to maintain PaCO_2_ at 38-43 mmHg (end-tidal CO_2_ [CO_2_ at end-expiration] of approximately 35-40 mmHg). The trend to use a low Vt during surgery requires the adoption of a high f.^(^ 17 ^,^ 19 ^)^

Discontinuation of mechanical ventilation

**Recommendation -** Discontinue MV in the postoperative period as early and as quickly as possible, as soon as the patient is hemodynamically stable, is under adequate analgesia, has no electrolyte disturbances, and has regained a sufficient level of consciousness to ensure maintenance of ventilatory drive and airway protection. Extubation can be performed in the operating room, in the post-anesthesia care unit, or in the ICU.^(^ 21 ^)^

Noninvasive ventilation after extubation

**Suggestion - **The use of NIV should be considered in patients undergoing cardiac, thoracic, bariatric, or upper abdominal surgery, because it is associated with better oxygenation and a lower incidence of atelectasis. It should be performed with low pressure levels in upper abdominal and esophageal surgery. NIV should not delay reintubation.

### **Recommendation -** NIV should not be used following the onset of an acute respiratory failure episode after extubation.^(^ 22 ^)^

## Mechanical ventilation in obese patients

**Comment -** Patients with a body mass index (BMI) ≥ 30 kg/m^2^ are considered obese. This condition is characterized by a series of physiological changes, such as decreased lung compliance, which is caused by the direct mechanical effect of fat distribution and an abnormal position of the diaphragm, because of increased intra-abdominal pressure (IAP), reduced functional residual capacity (FRC), and reduced total lung capacity (TLC), and by the increased work of breathing, because of increased airway resistance (Raw) and increased chest wall resistance, as well as a need for high minute volumes, leading to an increase in PaCO_2_.^(^ 23 ^,^ 24 ^)^

**Recommendation -** Consider every obese patient as potentially difficult airway. In obese patients with a Malampatti score ≥ 3 and a Cormack score of 3-4, as well as increased neck circumference, consider difficult airway and prepare the necessary infrastructure to manage this condition.^(^ 25 ^)^

**Suggestion - **Use the reverse Trendelenburg position during ventilation.^(^ 26 ^)^ The goal is to improve PaO_2_, static respiratory system compliance (Crs), and cardiac output, as well as reducing the development of atelectasis. 

### **Suggestion - **Avoid the supine position, because of reduced FRC, cardiac output, and increased work of breathing. If it is possible to use it, the beach chair position is suggested.^(^ 27 ^)^

**Suggestion - **Use NIV in cases of hypercapnic respiratory failure, taking the necessary precautions. Care should be exercised in the use of NIV in patients with a BMI ≥ 45kg/m^2^, because there is a higher risk of failure in this group.

### **Suggestion - **In invasive MV, no mode is superior to another mode. It is suggested that the assist-control mode (AC) as either PCV or VCV be initially used.^(^ 28 ^)^

### **Suggestion - **Monitor respiratory mechanics. Monitoring of IAP should be considered in cases of increased PaCO_2_ levels and/or increased airway pressures that cannot be explained by pulmonary causes.

### **Recommendation -** Use a Vt of 6mL/kg predicted body weight;^(^ 26 ^-^ 29 ^)^ for FiO_2_, it is suggested that the lowest level that can maintain oxygen saturation (SatO_2_) ≥ 92% be used.

**Suggestion - **For PEEP/recruitment maneuvers,^(^ 30 ^,^ 31 ^)^ the goal is to increase FRC, prevent the development of atelectasis, and reduce the risk of ventilation-induced lung injury. In addition, it is suggested that MRS be performed in cases of hypoxemia, decreased Vt, or increased PaCO_2_ levels. 

### **Suggestion - **Use PEEP levels ≥ 10 cmH_2_O.

### **Recommendation -** Maintain Pplat at ≤ 35 cmH_2_O.^(^ 32 ^)^

### **Suggestion - **In cases of moderate and severe ARDS, a Pplat of up to 40 cmH_2_O may be tolerable, provided that the distending pressure is maintained at ≤ 15 cmH_2_O.

### **Recommendation -** Extubate patients as soon as their clinical status allows, and NIV can be used to facilitate it.

## Mechanical ventilation in patients with central nervous system involvement

### Gas exchange - oxygen

### **Recommendation -** Avoid hypoxemia in patients with acute neurological injury, because it leads to increased morbidity and mortality.^(^ 33 ^,^ 34 ^)^

### **Suggestion - **Avoid hyperoxia in cases of anoxic-ischemic encephalopathy.^(^ 35 ^)^

### Gas exchange - carbon dioxide

### **Recommendation -** Do not use prophylactic or prolonged hyperventilation, and maintain PaCO_2_ between 35 and 40 mmHg during the acute phase of injury.^(^ 36 ^-^ 38 ^)^

### **Recommendation -** Acute hyperventilation is indicated as rescue therapy in cases of cerebral herniation.^(^ 36 ^-^ 38 ^)^

### **Recommendation -** Monitor CO_2_ by capnography. When this tool is unavailable, assess PaCO_2_ levels through blood gas sampling, more frequently during the acute phase.

### **Suggestion - **In patients with acute ischemic stroke, avoid PaCO_2 _< 35 mmHg because of risk of ischemia in the penumbra region.

### Acute respiratory distress syndrome

**Recommendation -** Use a protective ventilation strategy for the treatment of ARDS in patients with neurological injury, accompanied by intracranial pressure (ICP) and cerebral perfusion pressure monitoring.^(^ 29 ^,^ 39 ^)^ More details are provided in the related section of the present Recommendations.

**Suggestion -** In cases of severe ARDS, the use of a high PEEP should be individualized and ICP should be monitored, because increased ICP can occur when there is a concomitant decrease in lung and brain compliance.^(^ 40 ^,^ 41 ^)^

### Modes of ventilation

### **Suggestion -** Use the VCV mode for patients with severe neurological injury during the acute phase,^(^ 42 ^)^ to avoid oscillations in Vt. 

### **Recommendation -** Patients with severe neurological injury, with intracranial hypertension (ICH) during the acute phase, should not be maintained on a spontaneous mode of ventilation. ^(^ 43 ^)^

### Airway approach

### **Recommendation -** Intubate patients with a Glasgow Coma Scale score ≤ 8 and in whom the cough reflex is absent.^(^ 44 ^)^

**Suggestion -** Use rapid sequence intubation in patients with ICH or with suspected ICH, minimizing the risk of secondary brain injury due to increased intracranial pressure. This technique uses a combination of sedatives that have minimal cardiodepressant effects, such as ketamine (1-2 mg/kg i.v.) or etomidate (0.3 mg/kg i.v.), particularly in patients with hypotension or at risk of hypotension, and short-acting neuromuscular blocking agents (succinylcholine, 1.5 mg/kg i.v.) so that, within 45 to 60 seconds, the goal of paralysis and sedation is achieved.

### Unconventional ventilation strategies

**Suggestion - **In patients with severe pulmonary involvement, individualize the use of new ventilation strategies such as recruitment maneuvers, prone positioning, arteriovenous extracorporeal membrane CO_2_ removal (AV-ECCO_2_R), and extracorporeal membrane oxygenation (ECMO), assessing risk versus benefit on a case-by-case basis.^(^ 45 ^-^ 47 ^)^

### Head of the bed between 30º and 45º

### **Recommendation -** Maintain the head of the bed between 30º and 45º because this improves cerebral venous return and reduces the effect of PEEP on ICP.^(^ 48 ^)^

## Mechanical ventilation in patients with neuromuscular diseases

### **Comment -** In respiratory failure from neuromuscular disease, ventilatory support depends on the location of the injuries (from spinal cord injuries to direct muscle involvement) (Chart 1).

**Chart 1:**
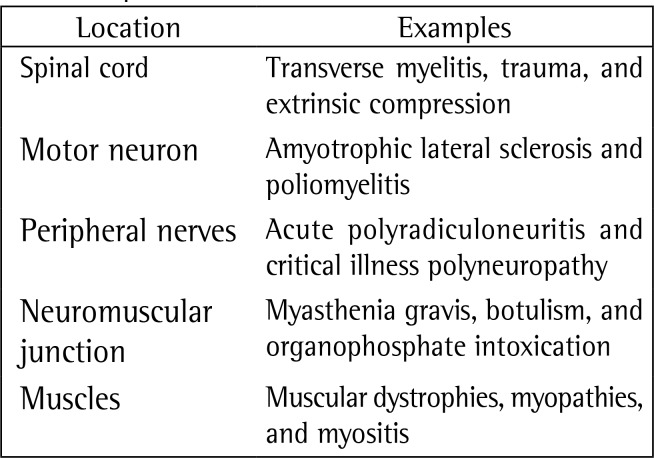
Location of the neuromuscular injuries and examples.

### Acute polyradiculoneuritis (Guillain-Barré syndrome)

**Comment -** One third of patients with acute polyradiculoneuritis (Guillain-Barré syndrome) require MV during the course of the disease. Generalized weakness, rapid progression, and bulbar involvement are associated with the need for MV in such patients.^(^ 49 ^-^ 52 ^)^

**Suggestion - **Patients with acute polyradiculoneuritis should be periodically assessed by measuring maximum inspiratory pressure (PImax) from residual volume, maximum expiratory pressure (PEmax) from TLC, and vital capacity (VC). Patients with PImax < −30 cmH_2_O, PEmax < 40 cmH_2_O, and VC < 20 mL/kg or a reduction of more than 30% in VC should be electively intubated to prevent emergency OTI.^(^ 49 ^-^ 52 ^)^

**Recommendation -** NIV should be used with care because of the instability of acute polyradiculoneuritis. Therefore, OTI and invasive MV should not be delayed when there is deterioration of lung function.^(^ 49 ^-^ 52 ^)^

**Suggestion - **The decision to tracheostomize patients with Guillain-Barré syndrome may be postponed for 2 weeks. If, after 2 weeks, lung function test results do no improve significantly, tracheostomy should be considered. If lung function test results are improving, tracheostomy may be postponed until weaning is achieved.^(^ 49 ^-^ 52 ^)^

### Myasthenia gravis

**Comment -** Acute respiratory failure results from a myasthenic crisis precipitated by neuromuscular junction failure, usually accompanied by bulbar weakness. Myasthenic-specific treatment with immunoglobulin or plasmapheresis should be initiated early.^(^ 53 ^-^ 55 ^)^

**Suggestion - **Consider using NIV (bilevel positive airway pressure [BiPAP]) in patients with myasthenic crisis in an attempt to prevent OTI, with PCO_2_ > 50mmHg being a predictor of BiPAP failure; the use of NIV can be considered in persistent or recurrent weakness following extubation.^(^ 53 ^-^ 55 ^)^

**Suggestion - **Periodically assess patients with myasthenic crisis by measuring PImax, PEmax, and VC. Patients with VC < 20 mL/kg, PImax < −30 cmH_2_O, and PEmax > 40 cmH_2_O can undergo a trial treatment with NIV (BiPAP) and, if there is failure, they should be electively intubated to prevent emergency OTI.^(^ 53 ^-^ 55 ^)^

**Suggestion - **Conduct an intensive respiratory program, including sighs, use of PEEP, frequent aspiration of the bronchial tree, respiratory therapy, body position change, and administration of antibiotic therapy (in cases of documented infection), in patients placed on MV for a myasthenic crisis.^(^ 53 ^-^ 55 ^)^

### Duchenne muscular dystrophy

**Comment -** Duchenne muscular dystrophy (DMD) is a recessive X-linked disease that affects 1:3,000 male births. It is characterized by progressive loss of muscle strength, with VC decreasing progressively until the onset of respiratory failure, usually between 18 and 20 years of age, with a consequent need for ventilatory support. Most patients develop cardiomyopathy. Forced VC (FVC) < 1L and the onset of nocturnal hypoventilation are signs of poor prognosis, and NIV can be used to improve survival and quality of life outcomes.^(^ 56 ^-^ 58 ^)^

### **Suggestion - **Use NIV in cases of nocturnal hypoventilation and/or decreased VC (< 1L).

### **Suggestion - **Invasive ventilation via elective tracheostomy is indicated for patients who do not tolerate NIV or who have been intubated for an acute event.^(^ 56 ^-^ 58 ^)^

### Amyotrophic lateral sclerosis

**Comment -** Amyotrophic lateral sclerosis (ALS) is a degenerative motor neuron disease, during the course of which respiratory failure due to muscle failure may occur. Chronic aspiration due to bulbar muscle dysfunction and ineffective cough are additional complications. Most patients die from respiratory complications that vary in course.^(^ 59 ^,^ 60 ^)^

### **Recommendation -** Use NIV in patients with ALS, except in the subgroup of patients with severe bulbar dysfunction.

**Recommendation -** Invasive MV via tracheostomy is indicated in patients with impaired airway protection and severe bulbar dysfunction, and it should only be instituted after its complications, as well as its social and logistical implications, are discussed in detail with the patients and their families.

**Recommendation -** Indications for ventilatory support include VC < 50% of predicted, PImax < −30 cmH_2_O or < 60% of predicted, peak expiratory flow (PEF) < 270 L/min, PCO_2_ > 45 mmHg, and nocturnal hypoventilation.^(^ 59 ^,^ 60 ^)^

**Suggestion - **The parameters for invasive and NIV are as follows. An oral or nasal mask can be used provided that it is properly fitted. BiPAP should be used. Invasive MV via tracheostomy is usually carried out in the ventilation mode that is most suitable for the type of demand, if there is associated lung disease. Patients should be monitored for episodes of atelectasis, accumulation of secretions, and pneumonias.^(^ 61 ^)^

## Mechanical ventilation in patients with cardiovascular disease

### **Comment -** The goal of MV in patients with cardiovascular disease is to adjust oxygenation and ventilation and to ensure cardiac output.

### **Recommendation -** Achieve an SpO_2_ ≥ 94% by using the lowest possible FiO_2_.

**Recommendation -** Noninvasive MV with either continuous positive airway pressure (CPAP) or BiPAP is safe, and both modes have similar effects and are effective in preventing OTI. They should be applied as a form of ventilatory support during acute pulmonary edema.^(^ 62 ^-^ 68 ^)^

### **Recommendation -** Apply the protective strategy to MV patients with cardiovascular disease.^(^ 69 ^,^ 70 ^)^

### **Recommendation -** Applying recruitment maneuvers is safe in patients with cardiovascular disease provided that there is proper monitoring and appropriate care.^(^ 69 ^,^ 70 ^)^

**Suggestion - **Monitoring of cardiac output and measurement of extravascular lung water are suggested in MV patients with cardiovascular disease and ARDS, with the aim of volume adjustment and hemodynamic optimization.^(^ 71 ^)^

**Suggestion - **Monitoring of cardiac output, in patients with cardiovascular disease, can be performed with a pulmonary artery catheter (PAC) or noninvasively, by means of pulse contour analysis.^(^ 69 ^,^ 70 ^)^

### **Suggestion - **Transthoracic echocardiography can be performed in MV patients with cardiovascular disease who are hemodynamically unstable.^(^ 70 ^)^

**Suggestion - **Perform transthoracic echocardiography in patients who exhibit hemodynamic instability after undergoing RM, in order to assess them for volume status and right ventricular (RV) dysfunction.^(^ 69 ^,^ 70 ^)^

**Recommendation -** Discontinuation of MV in patients with cardiovascular disease follows the recommendations for patients with no cardiovascular disease. The use of NIV should be prioritized to facilitate the process of discontinuation of MV, and NIV should be applied immediately after extubation.^(^ 62 ^-^ 70 ^)^

### **Suggestion - **Increased brain natriuretic peptide (BNP) levels during weaning from ventilation in patients with cardiovascular disease show accuracy in predicting weaning failure.^(^ 70 ^,^ 72 ^)^

### **Recommendation -** A positive fluid balance should be avoided in MV patients with cardiovascular disease who are hemodynamically stable.^(^ 70 ^)^

### **Recommendation -** Inhaled nitric oxide is an effective strategy in MV patients with cardiovascular disease, RV dysfunction, and pulmonary hypertension.^(^ 70 ^)^

### **Recommendation -** No mode of ventilation is recommended over another in patients with cardiovascular disease.^(^ 70 ^)^

### **Suggestion - **In patients on inotropic support, this support may be continued until after extubation.^(^ 70 ^)^

### Mechanical ventilation in patients with cardiovascular disease undergoing surgery

Tidal volume

### **Recommendation -** The use of a Vt of 6 mL/kg predicted body weight, in the volume-control mode or peak/plateau inspiratory pressure, is sufficient to maintain this same volume in PCV.^(^ 70 ^)^

Positive end-expiratory pressure

### **Recommendation -** Apply PEEP during general anesthesia, because it is associated with improved oxygenation and prevention of atelectasis.^(^ 70 ^)^

Alveolar recruitment maneuvers

### **Suggestion -** RM can be used intraoperatively to prevent alveolar collapse.^(^ 69 ^)^

Fraction of inspired oxygen

### **Recommendation -** At induction of anesthesia, use an FiO_2_ of 100% to ensure adequate oxygenation for intubation. Fractions of oxygen needed to maintain SpO_2_ > 94% are recommended.^(^ 70 ^)^

Discontinuation of mechanical ventilation

### **Recommendation -** Discontinuation of MV should be gradual, and pressure support ventilation (PSV) can be used. NIV is an important resource, which should be used immediately after extubation.^(^ 62 ^-^ 70 ^)^

Postoperative analgesia

### **Recommendation -** Achieving adequate postoperative analgesia is associated with optimization of postoperative pulmonary function.

## Mechanical ventilation in interstitial lung diseases

**Comment -** Interstitial lung diseases (ILDs) are a heterogeneous group of diseases that predominantly affect the lung interstitium, with varying degrees of inflammation and fibrosis,^(^ 73 ^,^ 74 ^)^ and that can progress with varying degrees of hypoxemia and gradual reduction in lung volumes. Patients with ILD may require MV because of a number of factors, including the anesthesia process in surgical procedures, such as open lung biopsy and other elective or emergency operations, respiratory infections leading to respiratory failure, and acute exacerbations (AEs - noninfectious) of the underlying interstitial disease.^(^ 75 ^,^ 76 ^)^

### Indications for mechanical ventilation

**Comment -** Respiratory failure in patients with ILD should be divided into two groups: progression of the underlying disease and AEs. AEs are characterized by acute, unexplained worsening of clinical symptoms of ILD, especially dyspnea and cough, usually in the last 30 days, accompanied by radiological worsening, often in the form of ground-glass changes superimposed on previous changes. AEs were initially described in patients with idiopathic pulmonary fibrosis (IPF), but they can occur in other forms of ILD.^(^ 76 ^-^ 79 ^)^ The onset of AEs seems to occur at some point in the course of IPF in 5 to 10% of patients, and mortality in those requiring MV is nearly 100%.

### **Recommendation -** Before diagnosing an AE, it is necessary to exclude infections, pulmonary thromboembolism, cardiac dysfunction, drug-induced pulmonary toxicity, etc.^(^ 76 ^-^ 80 ^)^

Acute complication

**Suggestion - **In AEs of ILD, the patient's previous status should be assessed. Invasive MV is indicated if the cause of acute respiratory failure is diagnosed as not being due to progression of the underlying disease.

Progression of the underlying disease

### **Recommendation -** ICU admission and invasive MV should be avoided, and any decision in this regard should be discussed with the patients or their families.

Noninvasive mechanical ventilation

**Suggestion - **NIV can be used as initial ventilatory support in patients with ILD who develop acute respiratory failure or as palliative ventilatory support in patients who have previously expressed the desire not to be intubated. Either CPAP or bilevel NIV can be used and should be applied early.

**Recommendation -** The use of NIV should be monitored at the bedside by a healthcare professional within thirty minutes to two hours. For NIV to be considered successful, the following criteria should be met: reduction of f, increase in Vt, improvement of the level of consciousness, reduction or cessation of the use of accessory muscles, increase in PaO_2_ and/or SpO_2_, and reduction of PaCO_2_ without significant abdominal distension. In unsuccessful cases, OTI and invasive MV should be performed immediately. Success is expected in 50% of this population.^(^ 80 ^)^

Noninvasive mechanical ventilation

**Comment -** Since the histological finding in AEs is diffuse alveolar damage (DAD), similar to that observed in patients with ARDS, and given the lack of prospective studies, some authors have suggested that strategies used for ARDS could be extrapolated to patients with AEs of ILD. Therefore, some experts advocate the use of protective ventilation, with a low Vt, set at approximately 6 mL/kg ideal body weight, and a Pplat ≤ 30 cmH_2_O.^(^ 80 ^-^ 84 ^)^ The use of a high PEEP in patients with ILD has not been investigated in any study. Two retrospective studies found an association between PEEP and the outcome of patients with ILD who underwent MV: Suh et al. reported low mortality in a group of patients with acute interstitial pneumonia who received an early intervention strategy, which involved a series of measures, including MV with a low Vt and a moderate PEEP, with a median of 11 cmH_2_O.^(^ 79 ^)^ In contrast, in a more heterogeneous group of patients with ILD who underwent MV, Fernandez-Perez et al. observed that a PEEP > 10 cmH_2_O on the first day of MV was associated with high mortality, but they themselves comment that a high PEEP may be a marker of greater respiratory failure severity.^(^ 76 ^)^

**Suggestion - **Patients with ILD who require MV should be ventilated with a low Vt, set at approximately 6 mL/kg ideal body weight, and a Pplat < 30 cmH_2_O; a high f (> 30 breaths/min) and a short inspiratory time can be used to prevent hypercapnia. Use a PEEP between 5 and 10 cmH_2_O.

**Suggestion - **The use of a high PEEP can be attempted with caution and should be individualized for each patient. Rescue therapies for refractory hypoxemia, such as prone positioning, RM, and nitric oxide, can be used in referral centers with expertise in these therapies.

## Discontinuation of mechanical ventilation

### Identifying patients who are ready to wean

### **Recommendation -** Discontinue patients from invasive MV as soon as it is clinically possible.^(^ 85 ^,^ 86 ^)^

**Recommendation -** The definitions of terms related to discontinuation of MV must be made clear in ICU guidelines. The concept of "successful weaning" refers to a patient successfully completing the spontaneous breathing trial (SBT) while still connected to the ventilator. "Successful extubation" refers to a patient having the endolaryngeal tube removed (extubation) after passing the SBT and not being reintubated in the following 48 hours. In tracheostomized patients, successful extubation means tolerating being disconnected from the ventilator after passing the SBT and not needing to be reconnected to the ventilator in the following 48 hours.^(^ 87 ^-^ 89 ^)^

**Recommendation -** Assess and identify patients daily (active surveillance by means of internal guidelines established by a multidisciplinary team) with a view to the possibility of discontinuing ventilation, in order to reduce duration of MV and decrease costs.^(^ 90 ^-^ 94 ^)^

### Sedation

**Recommendation -** Sedation should be interrupted daily to assess the patient's ability to maintain spontaneous ventilation^(^ 95 ^)^ (see the "Sedation and Analgesia" section in Part 1 of the present Recommendations).

### Criteria required for assessment of readiness to wean

### **Recommendation -** Perform active surveillance, including the topics in Chart 2.^(^ 88 ^,^ 90 ^-^ 96 ^)^

**Chart 2:**
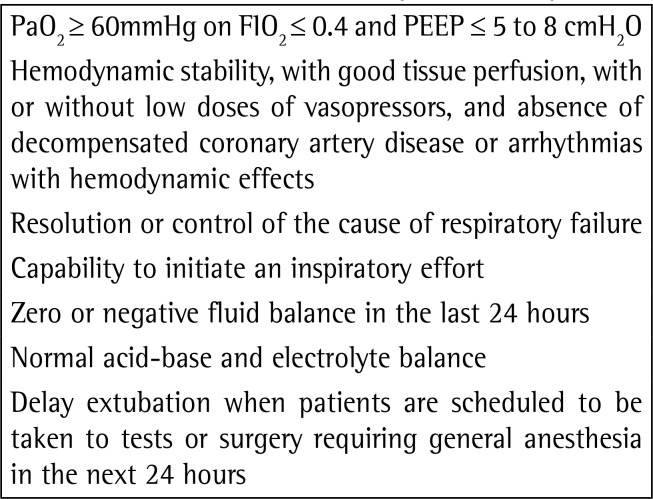
Topics that are to be routinely assessed during active surveillance in mechanically ventilated patients. PaO2: partial pressure of oxygen; FIO2: fraction of inspired oxygen; and PEEP: positive end-expiratory pressure.

### Predictive indices

**Chart 3:**
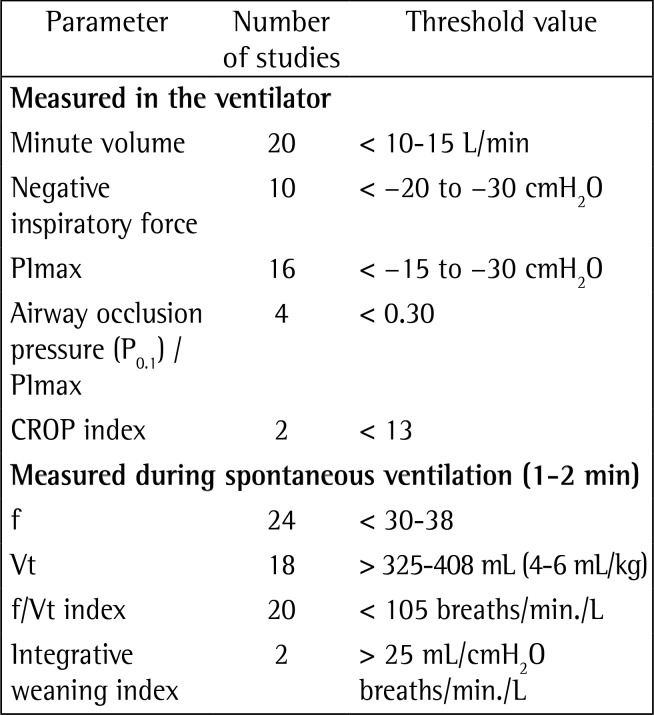
Significant parameters for predicting successful weaning. CROP: compliance, respiratory rate, oxygenation, and pressure; PImax: maximum inspiratory pressure; f: respiratory rate; and Vt: tidal volume.

**Recommendation -** The most accurate weaning-predictive indices are the rapid shallow breathing index (RSBI), i.e., f divided by Vt (f/Vt), and the integrative weaning index (IWI) (Chart 3). They should only be calculated in situation in which the decision is difficult and must NOT be used as the sole tool in the decision-making process regarding the SBT.^(^ 97 ^-^ 99 ^)^

### Autonomous breathing trial (spontaneous breathing trial)

**Chart 4:**
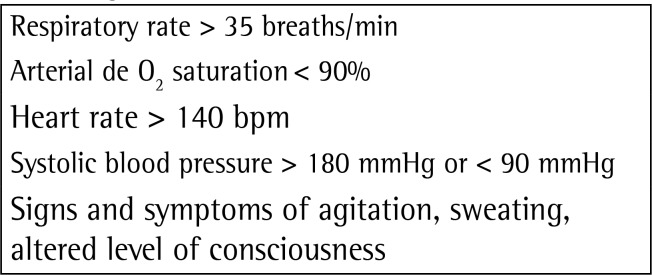
Signs of intolerance to the spontaneous breathing trial.

**Recommendation -** In the SBT, patients should be placed on T-tube or ventilated with PSV of 5-7 cmH_2_O for 30-120 minutes. During the SBT, patients should be monitored for signs of failure.^(^ 85 ^-^ 94 ^)^ Successful SBTs are defined as those in which patients maintain an adequate breathing pattern, as well as adequate gas exchange, hemodynamic stability, and comfort (Chart 4).^(^ 85 ^-^ 94 ^,^ 100 ^-^ 102 ^)^

### **Recommendation -** After a successful SBT, determine whether the airways are patent and whether the patient is able to protect them.

### How to determine the timing of extubation

Assessment of airway protection

**Recommendation -** Determine whether the patient has the required level of consciousness (a Glasgow coma scale score > 8), an effective cough (a positive white card test result and a peak flow > 60 lpm), and a small amount of secretions (with no need for suctioning every 1 or 2 hours).^(^ 103 ^)^

Assessment of airway patency

**Chart 5:**
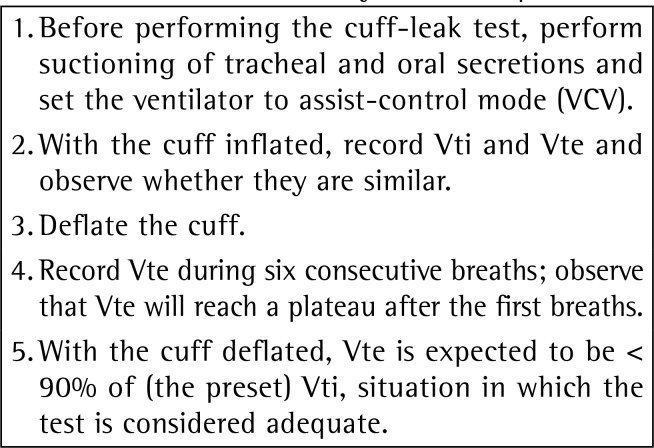
How to perform the endotracheal tube cuff-leak test in mechanically ventilated patients.

VCV: volume-cycled ventilation; Vte: expired tidal volume; and Vti: inspired tidal volume.

**Suggestion - **Test airway patency in patients at higher risk for laryngeal stridor and airway obstruction (prolonged ventilation and trauma); to that end, a qualitative or quantitative approach can be used. Perform thorough suctioning of the mouth and larynx before deflating the tube cuff for the test, in order to prevent unwanted material from entering the lower airways iatrogenically (Chart 5).^(^ 104 ^)^

### Use of corticosteroids

**Recommendation -** In patients at high risk for laryngeal stridor and laryngeal edema, as identified by the cuff leak test, the preventive use of corticosteroids may be beneficial. The prescribed doses range from 20 to 40 mg of i.v. methylprednisolone every 4 to 6 hours, started at least 4 hours, more commonly 12 to 24 hours, before extubation.^(^ 105 ^)^

### Use of noninvasive ventilation in the discontinuation of mechanical ventilation

Use of noninvasive ventilation to facilitate discontinuation of mechanical ventilation - early weaning (noninvasive ventilation as a facilitation technique)

**Figure 1:**
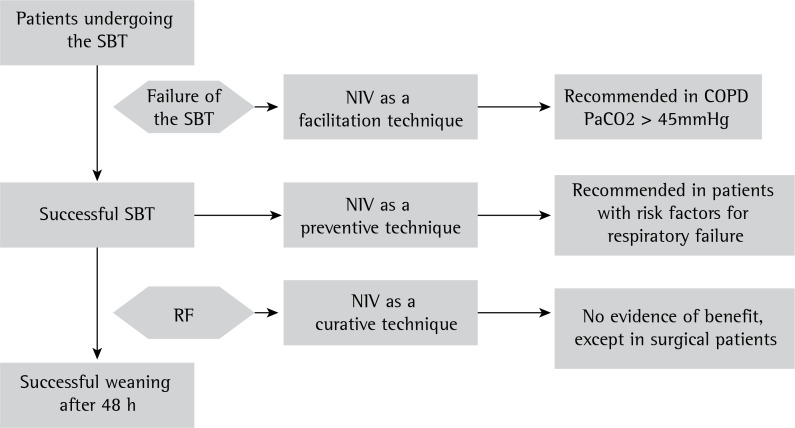
Use of noninvasive ventilation for discontinuation of mechanical ventilation. SBT - spontaneous breathing trial; NIV - noninvasive ventilation; COPD - chronic obstructive pulmonary disease; RF - acute or exacerbated respiratory failure.

**Recommendation -** It is recommended that NIV be used to facilitate early discontinuation of MV in patients with chronic obstructive pulmonary disease (COPD), even in those who fail the SBT, provided that their clinical status is adequate. The patient should be managed in centers with experience in the use of NIV (Figure 1).^(^ 106 ^)^

Use of noninvasive ventilation to prevent extubation failure (noninvasive ventilation as a preventive technique)

### **Recommendation -** NIV should be used immediately after extubation, in a preventive manner, in patients identified as high risk, especially in hypercapnic patients (Figure 1 and Chart 6).^(^ 107 ^-^ 111 ^)^

**Chart 6:**
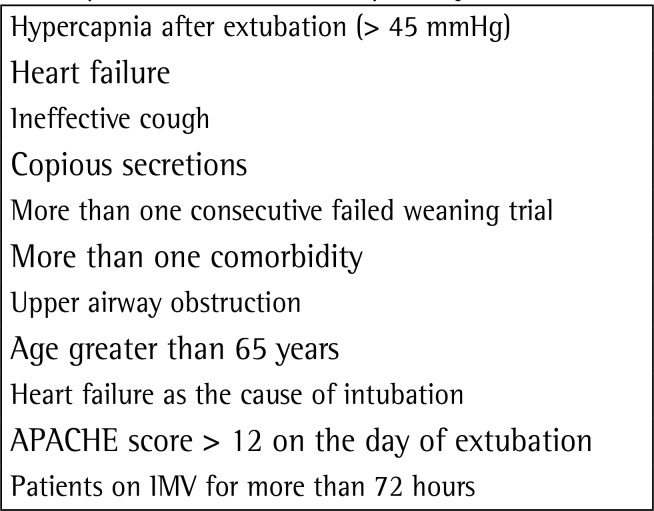
Noninvasive ventilation as a preventive technique: risk factors for respiratory failure. IMV: invasive mechanical ventilation.

Use of noninvasive ventilation in respiratory failure after extubation (noninvasive ventilation as a curative technique)

**Recommendation -** Avoid the use of NIV in extubated patients who again develop respiratory failure within 48 hours. Do not delay reintubation in this situation, except in surgical patients who develop respiratory failure in the postoperative period (Figure 1).^(^ 112 ^)^

How to manage patients with weaning failure (patients who fail their first spontaneous breathing trial)

**Recommendation -** Reinstate ventilatory support that provides patients with comfort and adequate gas exchange, maintaining it for 24 hours, so that the SBT can be repeated. Try to identify the causes of failure^(^ 86 ^)^ (for difficult-to-wean patients and those requiring long-term weaning, see the related section of the present Recommendations).

### Gradual weaning methods

### **Recommendation -** Avoid the use of synchronized inspiratory mandatory ventilation, because it can increase the time to discontinuation of invasive MV.^(^ 113 ^)^

### How to manage patients with extubation failure

**Recommendation -** Reintubate patients as soon as possible; identify and treat the causes of failure; and, as soon as possible, restart the discontinuation process (exception: noninvasive ventilation as a curative technique may be tried in surgical patients).

## Patients with prolonged weaning

**Suggestion - **Use classification definitions for the duration of the weaning process to categorize your patients as undergoing simple weaning^(^ 114 ^)^ (when patients successfully complete their first SBT), difficult weaning (when patients fail their first SBT and require up to three SBTs or up to seven days of MV following the first SBT), and, finally, prolonged weaning (when patients fail more than three consecutive SBTs or require >7 days of MV following the first SBT).

### **Suggestion - **Use the concept of prolonged MV defined as MV that is needed for ≥ 21 consecutive days for 6 hours daily.

### **Recommendation -** Identify causes of failure to discontinue MV (Chart 7).^(^ 88 ^,^ 114 ^-^ 130 ^)^

**Chart 7:**
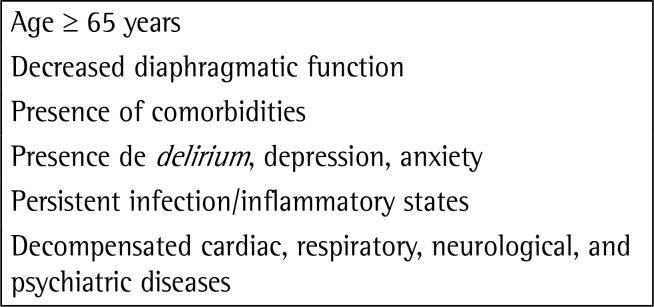
Causes of failure to discontinue mechanical ventilation.(89,115-131)

### Muscle disorders

### **Suggestion - **Assess the possibility of critical illness polyneuropathy and of phosphorus, magnesium, calcium, and potassium disorders.

### Endocrine and metabolic diseases

### **Recommendation -** Adequately control diabetes, hypothyroidism, and adrenal failure.

**Comment -** Obesity can be an additional factor for prolonged weaning, because it is characterized by an increase in O_2_ consumption (VO_2_) and CO_2_ production (VCO_2_), a decrease in static compliance, VC, and TLC, and a possible increase in IAP and Raw.

### Electrolyte and acid-base disturbances

**Recommendation -** Monitor, diagnose, and treat states of hyperhydration, which are associated with increased morbidity, mortality, and ICU length of stay. Identify and treat cases of metabolic alkalosis, the most common causes of which are chronic respiratory acidosis and use of diuretics. Metabolic alkalosis is associated with increased mortality, decreased respiratory drive, decreased O_2_ delivery (DO_2_), shift of the oxyhemoglobin curve to the left, ventilation/perfusion (V/Q) mismatch, and systemic vasoconstriction. Provide an adequate means of nutrition (see the related section of the present Recommendations) to prevent malnutrition, increased protein catabolism, reduced body muscle mass and decreased effectiveness of the thoracic pump mechanism, with reduced strength and endurance and increased VO_2_, and perpetual dependence on the ventilator. Decreased albumin is associated with prolonged weaning and should be monitored.^(^ 114 ^-^ 130 ^)^

### Rehabilitation strategies and strategies to facilitate discontinuation from mechanical ventilation

Reassessment of the underlying disease and comorbidities

### **Recommendation -** Treat cardiac, pulmonary, psychiatric, and infectious underlying diseases as much as possible, and maintain clinically adequate nutrition.

Specific care for discontinuation of mechanical ventilation

### **Suggestion - **Transfer patients undergoing prolonged weaning to a unit specializing in discontinuation of MV, if available.

**Recommendation -** Tracheostomy is indicated in patients who repeatedly fail SBTs, from the tenth day of MV onward, as part of a discontinuation protocol and in accordance with specifications in the related section of the present Recommendations.

**Suggestion - **Perform SBTs daily, using a tracheostomy collar or T-piece. In cases of tolerance (f < 35 breaths/minute or over 35 breaths/minute for less than 5 consecutive minutes; SaO2 > 90%; HR < 140 beats/minute or a sustained change of 20% in any direction; 90 mmHg > blood pressure < 180 mmHg with no anxiety or diaphoresis), gradually increase the duration of T-piece use and rest the patient overnight on assist-control ventilation. In cases of failure, return the patient to an assist-control mode for rest, in order to make a second attempt within 24 hours.^(^ 123 ^)^

**Suggestion - **Long-term MV is characterized when there is failure of the entire discontinuation process, especially in patients with spinal trauma, end-stage COPD, advanced dementia, pulmonary fibrosis, and irreversible neuromuscular disease. In these situations, explain the concept of futile treatment and palliative care to patients and families in order to arrive at a joint decision regarding the best approach.^(^ 114 ^)^

**Recommendation -** Early physical therapy and passive mobilization should be performed during MV and also during the discontinuation process. These activities are considered safe and are associated with better functional results and shorter duration of MV^(^ 121 ^,^ 130 ^)^ (see the related section of the present Recommendations).

### **Suggestion - **Inspiratory muscle training can be considered in patients who fail to wean, in order to increase PImax and facilitate discontinuation of ventilatory support.^(^ 130 ^,^ 131 ^)^

## Hemodynamic changes and hemodynamic care in patients on invasive mechancial ventilation

### **Comment -** The cardiovascular effects of positive pressure MV are described in Chart 8.

**Chart 8:**
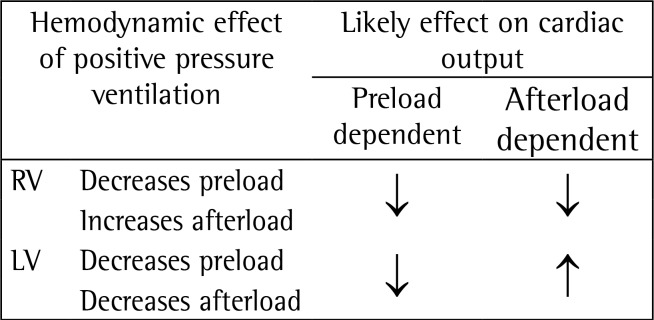
Cardiovascular effects of positive pressure mechanical ventilation. RV: right ventricle; and LV: left ventricle.

### Hemodynamic care in mechanically ventilated patients

**Recommendation -** Resuscitation aimed at restoration of tissue perfusion begins in the early acute phase, in which resuscitation should be performed as soon as possible to achieve mixed venous O_2_ saturation (SvO_2_) > 65% or central venous saturation (SvcO_2_) > 70%, blood lactate concentration < 2 mmol/L (18 mg/dL), and adequacy of DO_2_/VO_2_.^(^ 132 ^-^ 135 ^)^ The process should continue in the post-resuscitation phase (when adequate tissue perfusion is achieved), and fluid restriction should be performed to maintain a zero or negative fluid balance.^(^ 136 ^)^

**Suggestion - **In ARDS patients receiving PEEP ≥ 15 cmH_2_O and < 20 cmH_2_O, perform echocardiography at least once and, if necessary, monitor cardiac output.^(^ 137 ^,^ 138 ^)^ In ARDS patients receiving PEEP ≥ 20 cmH_2_O or experiencing hemodynamic instability, perform monitoring with serial echocardiography and/or with a volumetric PAC, if available.^(^ 137 ^,^ 138 ^)^

**Suggestion - **During MRS with decremental PEEP titration, use invasive blood pressure monitoring; perform echocardiography after 6 hours, or earlier if the patient exhibits hemodynamic instability, to assess RV dysfunction. ^(^ 137 ^,^ 138 ^)^

### **Suggestion - **In moderate/severe ARDS, consider extravascular lung water monitoring (if available).^(^ 139 ^,^ 140 ^)^

### **Suggestion - **Avoid systemic systemic vasodilators in refractory hypoxemia (they inhibit hypoxic vasoconstriction).^(^ 141 ^)^

### **Suggestion - **Interpret SvO_2_ by considering PaO_2_. High PaO_2_ values can increase SvO_2_.^(^ 142 ^)^

### Mechanical ventilation in patients with left ventricular failure

**Recommendation -** For the diagnosis of left ventricular (LV) failure, use Doppler echocardiography, which should show ejection fraction, velocity-time integral, as well as assess diastolic function [E/A, E/E', global end-diastolic volume].^(^ 143 ^)^ If you use a PAC, a pulmonary artery occlusion pressure >18 mmHg and a cardiac index < 2.2 L/min/m^2^ characterize LV failure.^(^ 143 ^)^

### **Recommendation -** Use inotropes, vasopressors if necessary, diuretics, and vasodilators when possible. In selected cases, use mechanical circulatory support.^(^ 143 ^)^

**Suggestion - **Favor the use of high PEEP (because of decreased LV preload and afterload). If there is concomitant RV failure, increase PEEP carefully (monitor the RV and RV flow).^(^ 144 ^)^ Prevent severe hypercapnia (pH < 7.15 or PaCO_2_ > 80 mmHg).^(^ 145 ^)^ Consider kidney ultrafiltration for achieving a negative fluid balance in situations of refractoriness to diuretics.^(^ 146 ^)^

### Mechanical ventilation in patients with right ventricular failure

**Recommendation -** Regarding diagnosis, use Doppler echocardiography (RV diastolic diameter > 3.5 cm, RV/LV ratio > 1, intraventricular septal flattening or paradoxical interventricular septal motion, pulmonary artery systolic pressure > 35 mmHg, tricuspid annular plane systolic excursion < 1.8 cm)^(^ 147 ^)^ or a PAC (a volumetric PAC, if available): central venous pressure > pulmonary artery occlusion pressure; mean pulmonary artery pressure > 25 mmHg; systolic index < 30 mL.min^-1^.m^-2^; RV end-diastolic volume index > 140 mL.m^-2^.^(^ 137 ^)^

### **Suggestion - **Details regarding monitoring, treatment, and specific care are shown in Chart 9 and Figure 2.

**Chart 9:**
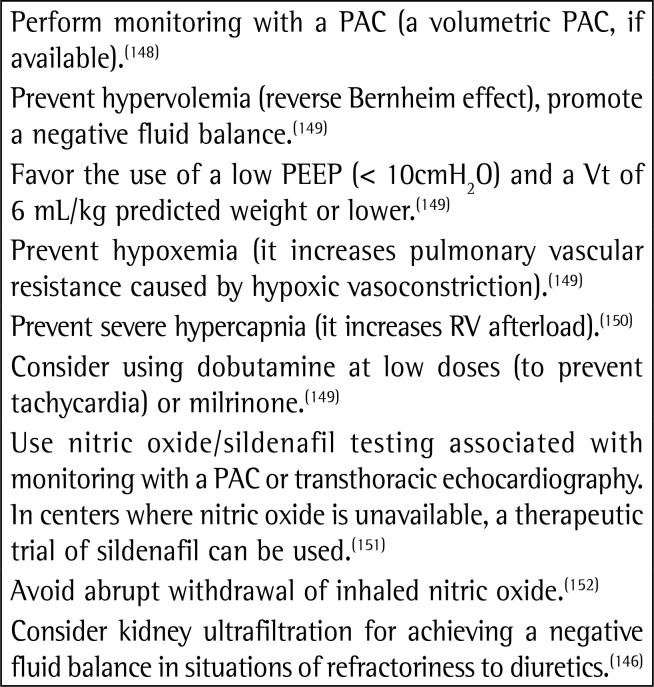
Suggestions for monitoring, treatment, and specific care in patients with right ventricular failure.(146,148-152) PAC: pulmonary artery catheter; PEEP: positive end-expiratory pressure; Vt: tidal volume; and RV: right ventricular.

**Figure 2:**
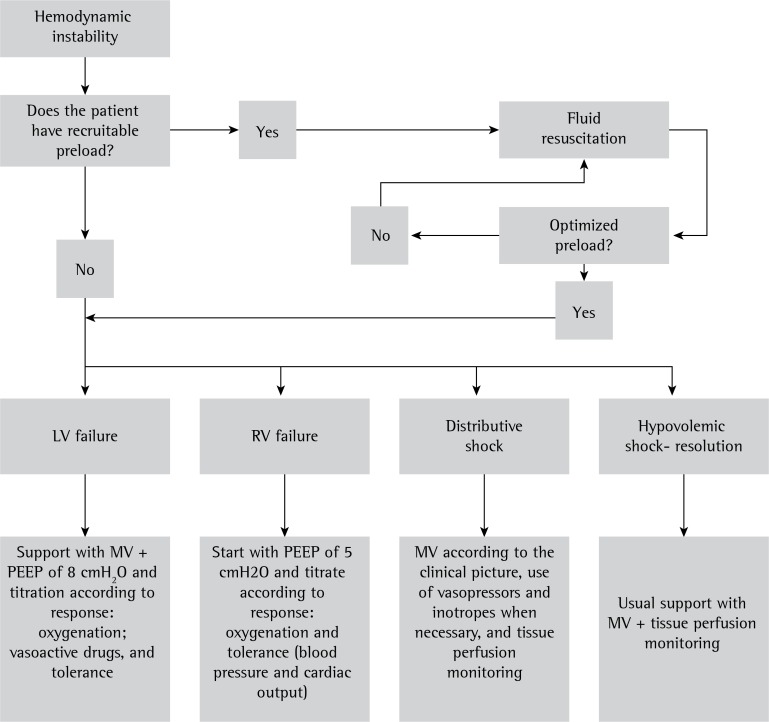
Hemodynamic management algorithm in mechanically ventilated patients. LV: left ventricular; RV: right ventricular; MV: mechanical ventilation; PEEP: positive end-expiratory pressure.

### Resources available for hemodynamic monitoring in mechanically ventilated patients

**Suggestion - **Suggested methods of hemodynamic monitoring in MV patients include predicting the response to fluid loading (a > 15% increase in the cardiac index) in patients with the following characteristics: PEEP < 10 cmH_2_O; Vt of 8-10 mL.kg-1 ideal body weight; f < 30 min-1; respiratory compliance > 30 mL.cmH_2_O; no arrhythmias; no respiratory effort; no cor pulmonale; delta pulse pressure > 13%; systolic volume variation > 10%; delta velocity time integral > 15%; superior vena cava collapsibility > 36%; and inferior vena cava distensibility > 18%. ^(^ 153 ^,^ 154 ^)^ Also perform the expiratory port occlusion maneuver - in spontaneously breathing patients receiving PEEP ≤ 10 cmH_2_O,^(^ 155 ^)^ with a delta central venous pressure > 1 mmHg (1.36 cmH_2_O).^(^ 156 ^)^ In patients receiving high PEEP and/or low Vt, passive leg raising maneuver,^(^ 157 ^)^ fluid challenge with small aliquots of fluid (250 mL), and cardiac index monitoring can be used (Figure 2).^(^ 158 ^)^

## Speech-language pathology care in the rehabilitation of patients after mechanical ventilation

**comment -** The role of speech-language pathologists in the ICU is endorsed by Resolution-RDC 07/2010 of the Brazilian *Agência Nacional de Vigilância Sanitária* (ANVISA, National Health Oversight Agency). In dysphagia, the role of speech-language pathologists in the ICU is regulated by Resolution 356 of the Brazilian Federal Speech-Language Pathology Council, passed on December 6, 2008, and published in the *Diário Oficial da União* (Official Federal Government Gazette) on December 9, 2008. In a multidisciplinary team, the speech-language pathologist evaluates the safety of oral feeding/swallowing and manages swallowing difficulties, thereby contributing to the prevention of aspiration pneumonia and to the tracheostomy weaning process.

**Suggestion - **Request a speech-language pathology evaluation^(^ 159 ^-^ 165 ^)^ for all patients who required OTI ≥ 48 hours, for patients who underwent repeated OTI, and for those with tracheostomy (whether or not they are mechanically ventilated). 

**Recommendation -** Regarding the timing for speech-language pathology evaluations after extubation, it is recommended that patients be evaluated 24^(^ 166 ^)^ to 48 hours after extubation and that those who have dysphagia and are at risk for aspiration be started on speech therapy.^(^ 167 ^-^ 171 ^)^

**Suggestion - **Do NOT perform speech-language therapy in orotracheally intubated patients, because there is a lack of clinical evidence regarding its benefits to the swallowing function; however, early identification of patients who, even during OTI, have several associated risk factors that may compromise swallowing dynamics (patients with cardiovascular disease, patients with Parkinson's disease, post-stroke patients, and patients with dementia) is indicated.

**Suggestion - **Perform clinical speech-language evaluations (structural and functional) at the bedside^(^ 163 ^,^ 172 ^)^ and determine the need for instrumental examination of swallowing (functional nasal fiberoptic endoscopy and modified barium swallow test).^(^ 173 ^)^

**Suggestion - **The speech-language pathologist should define the type of food consistency and the need for the use of thickeners for the administration of fluids, in collaboration with the Nutrition Service, when the patient is ready to initiate oral intake.

**Suggestion - **Fit a speaking valve into the mechanical ventilator circuit or directly into the tracheostomy, with the aid of a physical therapist and/or a physician, provided that cuff deflation is feasible and patient tolerance is assessed.^(^ 174 ^,^ 175 ^)^

**Suggestion - **In tracheostomized patients, use a blue food dye in the food delivered orally and/or in the saliva swallowing test, to evaluate the occurrence of discharge of blue saliva and/or secretions through the tracheostomy, characterizing an aspiration event.^(^ 176 ^-^ 178 ^)^

**Suggestion - **Assess for signs and symptoms of dysphagia during the delivery of oral nutrition, especially those that may be associated with bronchial aspiration, such as choking, cough, and wet voice.^(^ 179 ^-^ 181 ^)^

**Suggestion - **Discuss with the medical team the use of xerostomic medications in patients on MV and/or with tracheostomy who do not tolerate cuff deflation or who experience significant aspiration of saliva.^(^ 182 ^)^

### **Suggestion - **Perform direct and indirect swallowing therapy in patients who have dysphagia and/or are at risk for aspiration.^(^ 183 ^)^

## Nursing care in patients receiving invasive or noninvasive ventilatory support

### **Comment -** The nursing team, as part of the multidisciplinary ICU team, actively participates in the administrative and healthcare activities involving invasive and noninvasive support in MV patients.

### Care of circuits, filters, and humidifiers

### **Recommendation -** Maintain the lower airways warm and moist during MV. 

**Recommendation -** Replace (hygroscopic and hydrophobic) heat and moisture exchangers every 7 days, provided that appropriate height and positioning of the devices relative to the endotracheal tube are maintained (the devices should be connected vertically to the tube and the circuit so that they are not flooded with droplets and dirt). If there is dirt, condensation, or damage, the filter should be replaced.^(^ 184 ^)^

**Recommendation -** Do not replace the mechanical ventilator circuit routinely; replace it only when there is dirt visible to the naked eye, when there is damage, or in cases of prolonged ventilation (> 30 days).^(^ 185 ^,^ 186 ^)^

### Cleaning and maintenance of the equipment

### **Recommendation -** Mechanical ventilator circuits require high-level disinfection (5% sodium hypochlorite and a contact time of 60 minutes) or sterilization.^(^ 187 ^)^

### Precautions during bed bath and patient repositioning

**Recommendation -** Assess the vital signs, analyze and record the MV parameters (ventilation mode, peak pressure, PEEP, f, Vt, and FIO_2_), and check the alarms and clinical parameters before a bed bath and before patient repositioning. Continue cardiac monitoring and SatO_2_ monitoring during the bed bath and during patient repositioning. Allow a 5- to 10-minute equilibrium period before determining hemodynamic intolerance/instability due to patient repositioning and/or a bed bath.^(^ 188 ^,^ 189 ^)^

**Recommendation -** Work with the multidisciplinary team to determine the most appropriate time to perform a bed bath in critically ill, clinically unstable patients. The nurse should assess the patient before allowing the bath, postponing it in cases of severity that can compromise patient safety.

### **Recommendation -** Perform patient repositioning every 2 hours, with a lift sheet and at least two nursing professionals.^(^ 190 ^)^

### **Suggestion - **Perform continuous lateral rotation therapy with the use of a bed for kinetic therapy, when available.^(^ 191 ^)^

**Recommendation -** Maintain the head of the bed between 30º and 45º in MV patients. The evidence is conflicting as to aspiration of gastric content (45º) and pressure ulcers (30º). Positioning at 30° is preferred as long as it does not pose risks to or cause conflict with medical and nursing procedures.^(^ 192 ^)^

**Suggestion - **Use the beach chair position 2 to 4 times/day; it requires less personnel than do other interventions and therefore allows early mobility of ICU patients and improvement in pulmonary function.^(^ 193 ^)^

**Recommendation -** Maintain the cuff pressure of the endotracheal tube between 18 and 22 mmHg or between 25 and 30 cmH_2_O (cuff meter) in order to prevent air leaks without excessive compression of the tracheal mucosa. Avoid cuff pressures > 22 mmHg or 30 cmH_2_O. Check the cuff pressure at least 4 times/day and before performing oral hygiene.

**Recommendation -** Maintain the endotracheal tube secured and centralized by using an adhesive device or a shoelace so that the cuff pressure is homogeneously distributed in the trachea. Pay attention to lesions in the oral cavity, in the corners of the lips, and on the face.^(^ 194 ^)^

## **Recommendation -** The precautions to be followed during patient repositioning and while tilting the patient laterally in a bed bath are described in Chart 10.^(^ 195 ^)^

**Chart 10:**
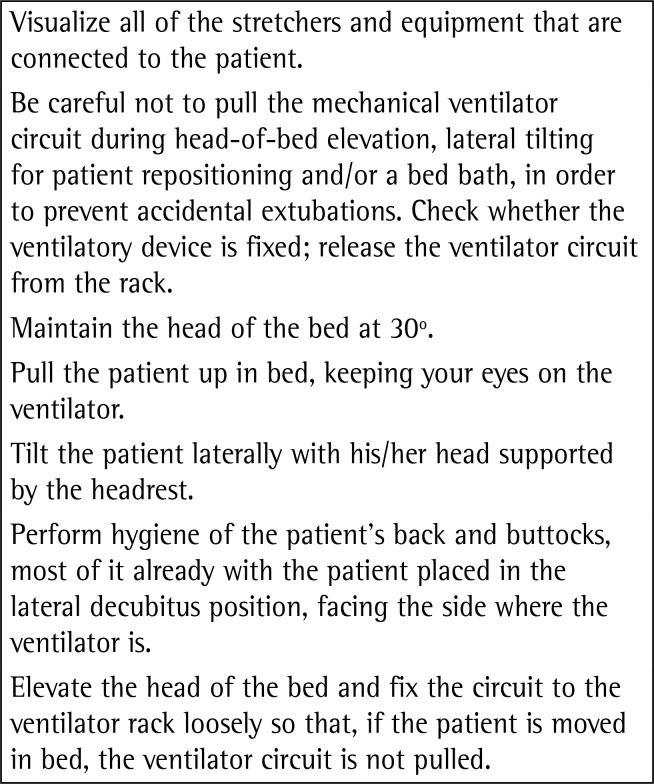
Precautions during patient repositioning and while tilting the patient laterally in a bed bath.

**Recommendation -** In patients being placed in the prone position, it is recommended that the procedure be performed in the presence of at least five members of the ICU team, including at least one physician and one nurse. The skin of the frontal region, nose, knees, iliac crest, genitalia, and nipples should be protected. Patient rotation should be performed in two steps, with total attention being paid to the invasive devices. The dorsal electrocardiogram should be monitored, and the reverse Trendelenburg position may be used to decrease facial edema.^(^ 194 ^,^ 195 ^)^

**Recommendation -** Use a closed suctioning system to perform tracheal suctioning in hemodynamically unstable patients, in order to prevent desaturation in at-risk patients (i.e., patients with cardiovascular disease) and to maintain alveolar recruitment and prevent atelectasis in ARDS patients receiving PEEP ≥ 10 cmH_2_O. The system should be changed every 7 days. Closed suctioning systems have not been shown to reduce the occurrence of ventilator-associated pneumonia, mortality, or ICU length of stay, when compared with open systems.^(^ 196 ^)^

### Specific instructions for oral hygiene, oral feeding, and enteral feeding

**Recommendation -** It is recommended that oral hygiene with brushing be performed every 12 hours, with an aqueous solution containing 0.12% chlorhexidine gluconate. In the intervals, oral hygiene should be performed with distilled or filtered water and/or alcohol-free mouthwash four times/day.^(^ 196 ^-^ 198 ^)^

### **Recommendation -** Check the cuff pressure of the endotracheal tube or tracheostomy before performing oral hygiene.^(^ 189 ^)^

**Recommendation -** The gastric and post-pyloric routes can be used for enteral feeding in MV patients, with post-pyloric tube placement being reserved for patients with gastric intolerance and/or contraindication.^(^ 200 ^)^

### **Recommendation -** Secure the nasogastric tube with a securing device (a commercially available nasal bridle or an adhesive device) in order to reduce the rate of unintentional tube dislodgement.^(^ 201 ^)^

### **Suggestion - **Monitor the difference between prescribed and delivered enteral nutrition as a marker of dietary compliance.^(^ 202 ^)^

## Physical therapy care in patients on ventilatory support

**Comments -** ICU patients may experience respiratory and muscle dysfunction and, over time, develop neuromuscular weakness and complications of immobility, which can make discontinuation of MV difficult. Prolonged immobility leads to loss of motor function and loss of quality of life, both of which can be minimized with the institution of early mobilization and respiratory care. The incidence of ICU-acquired muscle weakness (neuromuscular weakness) in patients requiring prolonged MV ranges from 25 to 60%,^(^ 203 ^)^ which contributes to increasing ICU and hospital length of stay. Physical therapy works to maintain and/or restore the functionality of the patient by preventing musculoskeletal changes and respiratory complications.

### **Recommendation -** A physical therapy diagnosis should precede any intervention.^(^ 204 ^)^

**Recommendation -** Physical therapy in MV patients in the ICU should be delivered 24 hours/day, having benefits in reducing duration of MV, ICU and hospital length of stay, hospital costs, and mortality.^(^ 200 ^,^ 205 ^)^

### Physical therapy maneuvers and approaches in mechanically ventilated patients

**Recommendation -** Bronchial hygiene therapy (positioning, manual inflation, vibration, and chest compression): indicated in patients with increased Raw due to the presence of secretions, causing asynchrony of MV and/or reduced oxygenation; and mandatory in lobar atelectasis.^(^ 206 ^)^

### **Suggestion - **Lung expansion techniques can be used in the presence of lung collapse, with reduced compliance and oxygenation.^(^ 207 ^)^

**Recommendation -** Perform inspiratory muscle training in patients with inspiratory muscle weakness who are undergoing prolonged MV, in order to improve muscle strength. The role of inspiratory muscle training in reducing duration of MV and successfully discontinuing MV has yet to be established.^(^ 208 ^)^

### Early mobilization in noninvasive and invasive mechanical ventilation

**Recommendation -** Early mobilization should be initiated less than 72 hours following the initiation of mechanical ventilation, because it is feasible, safe, and results in significant functional benefits.^(^ 206 ^)^

### **Suggestion - **Neuromuscular electrical stimulation and a cycle ergometer can be considered as a complement to the early mobilization program.^(^ 208 ^)^

**Suggestion - **Training for transfer from sitting to standing can be included in the treatment plan and precede ambulation, taking the correlation with functional limitation into account, as consensually determined by the multidisciplinary team.^(^ 209 ^)^

### **Suggestion - **Intervention in functional decline can be used to increase the chances of a return to independent performance of activities of daily living after discharge.^(^ 209 ^)^

## Nutritional care in mechanically ventilated patients

### Determination of caloric needs

**Suggestion - **Use indirect calorimetry or predictive formulas (equations or a pocket formula) to determine the caloric needs of critically ill MV patients. Indirect calorimetry should be considered when available, but it is necessary to take the patient's clinical status into account, as well as the frequency it will be used. There is not enough evidence to indicate that any of the available formulas in the literature is superior to the others.^(^ 210 ^-^ 214 ^)^ Chart 11 suggests the formulas most commonly used in daily practice.

**Chart 13:**
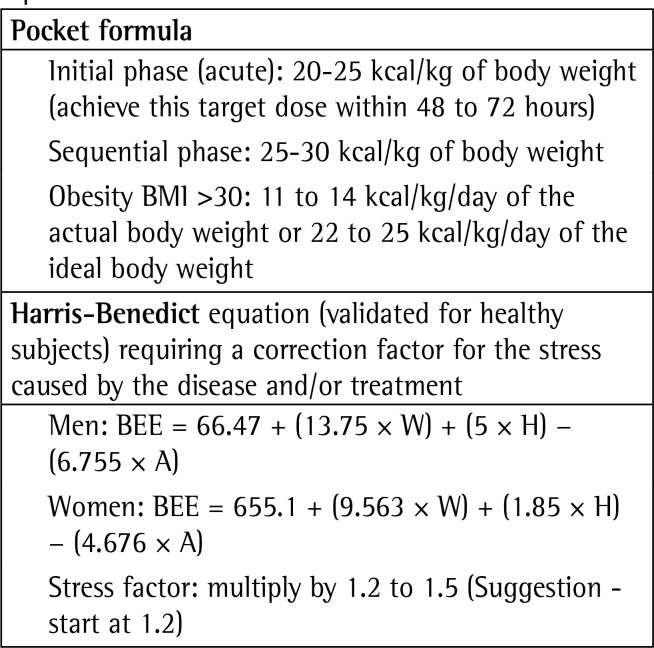
Pocket formula and the Harris-Benedict equation. BMI: body mass index; BEE: basal energy expenditure; W: weight; H: height; and A: age.

**Recommendation -** Initiate the enteral diet by delivering a small amount (20 to 25% of the target dose) and progressively increase it until achieving the target dose within 48 to 72 hours, in order to avoid the risk of refeeding syndrome. Before each increase, assess tolerability.

### Determination of protein needs

### **Suggestion - **Determine the amount of protein for MV patients on the basis of their BMI,^ 210-215)^ as shown in Chart 12. 

**Chart 12:**
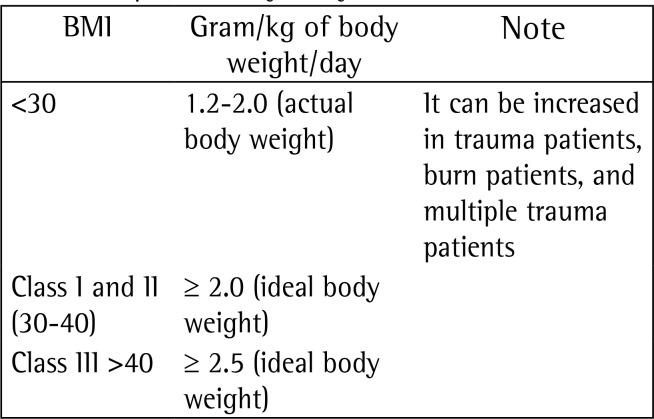
Amount of protein for mechanically ventilated patients, by body mass index.

**Suggestion - **Individualize the protein needs for critically ill MV patients with acute renal dysfunction. An important aspect to consider is that these patients should not receive a protein- restricted diet as a means to prevent or delay renal replacement therapy. Consider that patients on renal replacement therapy experience a significant loss of amino acids (10 to 15 g) during a dialysis session.^(^ 210 ^-^ 216 ^)^ In patients who are not candidates for dialysis, special diets formulated for nephropathic patients can be used.

### Routes of administration

### **Recommendation -** Use the enteral route as the primary option, whenever there is viability of the gastrointestinal tract.^(^ 211 ^,^ 217 ^)^

### **Suggestion - **Avoid using parenteral nutrition in critically ill MV patients until all strategies to optimize enteral nutrition (EN) have been attempted.

Early enteral nutrition

**Recommendation -** Initiate early EN (within 24 to 48 hours after admission to the ICU), provided that the patient is hemodynamically stable. Early EN therapy has been shown to reduce the mortality rate in critically ill MV patients and has been associated with a reduction in infectious complications and hospital length of stay.^(^ 208 ^-^ 211 ^,^ 217 ^,^ 218 ^)^

### Strategy to optimize delivery of enteral nutrition and minimize risks in mechanically ventilated patients(219)

Elevation of the head of the bed

### **Recommendation -** The head of the bed should be maintained between 30^o^ and 45^o^, unless there is a contraindication, for all intubated patients receiving EN.^(^ 200 ^,^ 210 ^,^ 220 ^)^

Tube placement for nutrition

**Recommendation -** Two routes (gastric and/or post-pyloric) should be considered in MV patients, with post-pyloric tube placement being reserved for patients with gastric intolerance and/or contraindication.^(^ 200 ^,^ 211 ^)^

### **Suggestion - **Consider gastrostomy or jejunostomy in MV patients requiring EN < 4 weeks, according to the patient's clinical status.^(^ 221 ^)^

Monitoring of gastric residuals in mechanically ventilated patients

**Recommendation -** Do not use monitoring of gastric residual volumes in MV patients with the aim of preventing ventilator-associated pneumonia. ^(^ 222 ^,^ 223 ^)^ As a positive effect, early EN without monitoring of gastric residuals in MV patients has been shown to improve delivery of EN.

### Continuous enteral nutrition compared with other methods

### **Suggestion - **The continuous method using an infusion pump^(^ 210 ^)^ can be used in critically ill MV patients with intolerance to EN.

### Establishing a nutritional therapy protocol

### **Suggestion - **Guidelines can be implemented in the facility to optimize EN in MV patients, in order to mitigate the calorie-protein deficit.^(^ 224 ^)^

### **Suggestion - **Prokinetic agents (preferably metoclopramide) can be used to improve tolerance, in order to attain the enteral caloric goal.^(^ 210 ^)^

### Specific care

Lipid-rich, carbohydrate-poor diet

**Suggestion - **(Lipid-rich, carbohydrate-poor) formulations designed to manipulate the respiratory quotient and reduce CO_2_ production can be used in selected patients (COPD patients with CO_2_ retention, patients with severe ARDS and permissive hypercapnia receiving borderline protective ventilation, those with CO_2_ retention undergoing difficult or prolonged weaning). Efforts should be made to prevent excess total calories.^(^ 200 ^,^ 210 ^,^ 225 ^)^

Enteral feeding enriched with fish oil, borage oil, antioxidant vitamins

**Suggestion - **Enteral formulations with an anti-inflammatory lipid profile and with oxidants can be used in MV patients with ARDS.^(^ 200 ^,^ 211 ^,^ 226 ^- 229)^ High doses of omega-3 should be avoided in patients who have coagulation disorders.^(^ 200 ^,^ 211 ^,^ 226 ^-^ 229 ^)^

Phosphorus replacement

**Suggestion - **Correction of phosphorus deficit is desirable in MV patients. This is justified by the association between hypophosphatemia and failure to discontinue MV.^(^ 230 ^)^

## References

[1] Guyatt GH, Oxman AD, Vist GE, Kunz R, Falck-Ytter Y, Alonso-Coello P, Schünemann HJ, GRADE Working Group (2008). GRADE: an emerging consensus on rating quality of
evidence and strength of recommendations. BMJ.

[2] Guyatt GH, Oxman AD, Kunz R, Vist GE, Falck-Ytter Y, Schünemann HJ, GRADE Working Group (2008). What is "quality of evidence" and why is it important to
clinicians?. BMJ.

[3] Guyatt GH, Oxman AD, Kunz R, Falck-Ytter Y, Vist GE, Liberati A, Schünemann HJ, GRADE Working Group (2008). Going from evidence to recommendations. BMJ.

[4] Brozek J, Oxman AD, Schünemann HJ GRADEpro (Computer Program) Version 3.2 for Windows.

[5] Hernandez G, Fernandez R, Lopez-Reina P, Cuena R, Pedrosa A, Ortiz R (2010). Noninvasive ventilation reduces intubation in chest
trauma-related  hypoxemia: a randomized clinical trial. Chest.

[6] Bolliger CT, Van Eeden SF (1990). Treatment of multiple rib fractures. Randomized
controlled trial comparing ventilatory with nonventilatory
management. Chest.

[7] Duggal A, Perez P, Golan E, Tremblay L, Sinuff T (2013). Safety and efficacy of noninvasive ventilation in
patients with blunt chest trauma: a systematic review. Crit Care.

[8] Chiumello D, Coppola S, Froio S, Gregoretti C, Consonni D (2013). Noninvasive ventilation in chest trauma: systematic
review and meta-analysis. Intensive Care Med.

[9] Gunduz M, Unlugenc H, Ozalevli M, Inanoglu K, Akman H (2005). A comparative study of continuous positive airway
pressure (CPAP) and intermittent positive pressure ventilation (IPPV) in patients
with flail chest. Emerg Med J..

[10] Round JA, Mellor AJ (2010). Anaesthetic and critical care management of thoracic
injuries. J R Army Med Corps.

[11] Carrier FM, Turgeon AF, Nicole PC, Trépanier CA, Fergusson DA, Thauvette D (2009). Effect of epidural analgesia in patients with traumatic
rib fractures: a systematic review and meta-analysis of randomized controlled
trials. Can J Anaesth.

[12] Smetana GW, Lawrence VA, Cornell JE, American College of Physicians (2006). Preoperative pulmonary risk stratification for
noncardiothoracic surgery: systematic review for the American College of
Physicians. Ann Intern Med.

[13] Canet J, Gallart L (2013). Predicting postoperative pulmonary complications in the
general population. Curr Opin Anaesthesiol.

[14] Hedenstierna G (2012). Oxygen and anesthesia: what lung do we deliver to the
post-operative ward?. Acta Anaesthesiol Scand.

[15] Heimberg C, Winterhalter M, Strüber M, Piepenbrock S, Bund M (2006). Pressure-controlled versus volume-controlled one-lung
ventilation for MIDCAB. Thorac Cardiovasc Surg.

[16] Gupta SD, Kundu SB, Ghose T, Maji S, Mitra K, Mukherjee M (2012). A comparison between volume-controlled ventilation and
pressure-controlled ventilation in providing better oxygenation in obese patients
undergoing laparoscopic cholecystectomy. Indian J Anaesth.

[17] Hemmes SN, Serpa A, Schultz MJ (2013). Intraoperative ventilatory strategies to prevent
postoperative pulmonary complications: a meta-analysis. Curr Opin Anaesthesiol.

[18] Serpa A, Cardoso SO, Manetta JA, Pereira VG, Espósito DC, Pasqualucci Mde O (2012). Association between use of lung-protective ventilation
with lower tidal volumes and clinical outcomes among patients without acute
respiratory distress syndrome: a meta-analysis. JAMA.

[19] Futier E, Constantin JM, Paugam-Burtz C, Pascal J, Eurin M, Neuschwander A, Marret E, Beaussier M, Gutton C, Lefrant JY, Allaouchiche B, Verzilli D, Leone M, De Jong A, Bazin JE, Pereira B, Jaber S, IMPROVE Study Group (2013). A trial of intraoperative low-tidal-volume ventilation
in abdominal surgery. N Engl J Med.

[20] Unzueta C, Tusman G, Suarez-Sipmann F, Böhm S, Moral V (2012). Alveolar recruitment improves ventilation during
thoracic surgery: a randomized controlled trial. Br J Anaesth.

[21] de Abreu MG, Pelosi P (2013). How can we prevent postoperative pulmonary
complications?. Curr Opin Anaesthesiol.

[22] Neligan PJ (2012). Postoperative noninvasive ventilation. Anesthesiol Clin.

[23] Jones RL, Nzekwu MM (2006). The effects of body mass index on lung
volumes. Chest..

[24] Pelosi P, Croci M, Ravagnan I, Tredici S, Pedoto A, Lissoni A (1998). The effects of body mass on lung volumes, respiratory
mechanics, and gas exchange during general anesthesia. Anesth Analg.

[25] Brodsky JB, Lemmens HJ, Brock-Utne JG, Vierra M, Saidman LJ (2002). Morbid obesity and tracheal intubation. Anesth Analg.

[26] Perilli V, Sollazzi L, Bozza P, Modesti C, Chierichini A, Tacchino RM (2000). The effects of the reverse trendelenburg position on
respiratory mechanics and blood gases in morbidly obese patients during bariatric
surgery. Anesth Analg.

[27] Valenza F, Vagginelli F, Tiby A, Francesconi S, Ronzoni G, Guglielmi M (2007). Effects of the beach chair position, positive
end-expiratory pressure, and pneumoperitoneum on respiratory function in morbidly
obese patients during anesthesia and paralysis. Anesthesiology.

[28] Aldenkortt M, Lysakowski C, Elia N, Brochard L, Tramèr MR (2012). Ventilation strategies in obese patients undergoing
surgery: a quantitative systematic review and meta-analysis. Br J Anaesth.

[29] (2000). Ventilation with lower tidal volumes as compared with
traditional tidal volumes for acute lung injury and the acute respiratory distress
syndrome. The Acute Respiratory Distress Syndrome Network. N Engl J Med.

[30] O'Brien JM Jr, Welsh CH, Fish RH, Ancukiewicz M, Kramer AM, National Heart, Lung, and Blood Institute Acute Respiratory Distress
Syndrome Network (2004). Excess body weight is not independently associated with
outcome in mechanically ventilated patients with acute lung injury. Ann Intern Med.

[31] Sprung J, Whalley DG, Falcone T, Wilks W, Navratil JE, Bourke DL (2003). The effects of tidal volume and respiratory rate on
oxygenation and respiratory mechanics during laparoscopy in morbidly obese
patients. Anesth Analg.

[32] Reinius H, Jonsson L, Gustafsson S, Sundbom M, Duvernoy O, Pelosi P (2009). Prevention of atelectasis in morbidly obese patients
during general anesthesia and paralysis: a computerized tomography
study. Anesthesiology.

[33] Stochetti N, Furlan A, Volta F (1996). Hypoxemia and arterial hypotension at the accident scene
in head injury. J Trauma.

[34] Chesnut RM, Marshall LF, Klauber MR, Blunt BA, Baldwin N, Eisenberg HM (1993). The role of secondary brain injury in determining
outcome from severe head injury. J Trauma..

[35] Bellomo R, Bailey M, Eastwood GM, Nichol A, Pilcher D, Hart GK, Reade MC, Egi M, Cooper DJ, Study of Oxygen in Critical Care (SOCC) Group (2011). Arterial hyperoxia and in-hospital mortality after
resuscitation from cardiac arrest. Crit Care.

[36] Curley G, Kavanagh BP, Laffey JG (2010). Hypocapnia and the injured brain: more harm than
benefit. Crit Care Med.

[37] Muizelaar JP, Marmarou A, Ward JD, Kontos HA, Choi SC, Becker DP (1991). Adverse effects of prolonged hyperventilation in
patients with severe head injury: a randomized clinical trial. J Neurosurg.

[38] Marion DW, Puccio A, Wisniewski SR, Kochanek P, Dixon CE, Bullian L (2002). Effect of hyperventilation on extracellular
concentrations of glutamate, lactate, pyruvate, and local cerebral blood flow in
patients with severe traumatic brain injury. Crit Care Med..

[39] Amato MB, Barbas CV, Medeiros DM, Magaldi RB, Schettino GP, Lorenzi-Filho G (1998). Effect of a protective-ventilation strategy on mortality
in the acute respiratory distress syndrome. New Engl J Med.

[40] Caricato A, Conti G, Della Corte F, Mancino A, Santilli F, Sandroni C (2005). Effects of PEEP on the intracranial system of patients
with head injury and subarachnoid hemorrhage: the role of respiratory system
compliance. J Trauma.

[41] McGuire G, Crossley D, Richards J, Wong D (1997). Effects of varying levels of positive end-expiratory
pressure on intracranial pressure and cerebral perfusion pressure. Crit Care Med.

[42] Pelosi P, Ferguson MD, Frutos-Vivar F, Anzueto A, Putensen C, Raymondos K, Apezteguia C, Desmery P, Hurtado J, Abroug F, Elizalde J, Tomicic V, Cakar N, Gonzalez M, Arabi Y, Moreno R, Esteban A, Ventila Study Group (2011). Management and outcome of mechanically ventilated
neurologic patients. Crit Care Med.

[43] Jaskulka R, Weinstabl C, Schedl R (1993). The course of intracranial pressure during respirator
weaning after severe craniocerebral trauma. Unfallchirurg.

[44] Chan B, Gaudry P, Grattan-Smith TM, McNeil R (1993). The use of Glasgow Coma Scale in
poisoning. J Emerg Med.

[45] Bein T, Kuhr LP, Bele S, Ploner F, Keyl C, Taeger K (2002). Lung recruitment maneuver in patients with cerebral
injury: effects on intracranial pressure and cerebral metabolism. Intensive Care Med.

[46] Reinprecht A, Greher M, Wolfsberger S, Dietrich W, Illievich UM, Gruber A (2003). Prone position in subarachnoid haemorrhage patients with
acute respiratory distress syndrome: effects on cerebral tissue oxygenation and
intracranial pressure. Crit Care Med.

[47] Yen TS, Liau CC, Chen YS, Chao A (2008). Extracorporeal membrane oxygenation resuscitation for
traumatic brain injury after decompressive craniotomy. Clin Neurol Neurosurg.

[48] Abbushi W, Herkt G, Speckner E, Birk M (1980). Intracranial pressure - variations in brain-injured
patients caused by PEEP-ventilation and lifted position of the upper part of the
body (author's transl). Anaesthesist.

[49] Vianello A, Bevilacqua M, Arcaro G, Gallan F, Serra E (2000). Non-invasive ventilatory approach to treatment of acute
respiratory failure in neuromuscular disorders. A comparison with endotracheal
intubation. Intensive Care Med.

[50] Mehta S (2006). Neuromuscular disease causing acute respiratory
failure. Respir Care.

[51] Lawn ND, Fletcher DD, Henderson RD, Wolter TD, Wijdicks EF (2001). Anticipating mechanical ventilation in Guillain-Barré
syndrome. Arch Neurol..

[52] Lawn ND, Wijdicks EF (2000). Post-intubation pulmonary function test in
Guillain-Barré syndrome. Muscle Nerve.

[53] Varelas PN, Chua HC, Natterman J, Barmadia L, Zimmerman P, Yahia A (2002). Ventilatory care in myasthenia gravis crisis: assessing
the baseline adverse event rate. Crit Care Med.

[54] Ambrosino N, Carpenè N, Gherardi M (2009). Chronic respiratory care for neuromuscular diseases in
adults. Eu Respir J..

[55] Rabinstein A, Wijdicks EF (2002). BiPAP in acute respiratory failure due to myasthenic
crisis may prevent intubation. Neurology.

[56] Eng D. (2006). Management guidelines for motor neurone disease patients
on non-invasive ventilation at home. Palliat Med.

[57] Radunovic A, Annane D, Rafiq MK, Mustfa N (2013). Mechanical ventilation for amyotrophic lateral
sclerosis/motor neuron disease. Cochrane Database Syst Rev..

[58] Finder JD, Birnkrant D, Carl J, Farber HJ, Gozal D, Iannaccone ST, Kovesi T, Kravitz RM, Panitch H, Schramm C, Schroth M, Sharma G, Sievers L, Silvestri JM, Sterni L (2004). American Thoracic Society. Respiratory care of the
patient with Duchene muscular dystrophy: ATS consensus statement. Am J Respir Crit Care Med.

[59] Pascoal IA, Villalba WO, Pereira MC (2007). Insuficiencia respiratória crônica nas doenças
neuromusculares: diagnóstico e tratamento. J Bras Pneumol.

[60] National Institute for Health and Clinical Excelence 2010.
Motor neurone disease: The use of non-invasive ventilation in the management of
motor neurone disease.

[61] Wards S, Chatwin M, Heather S, Simonds AK (2005). Randomised controlled trial of non-invasive ventilation
(NIV) for nocturnal hypoventilation in neuromuscular and chest wall disease in
patients with daytime normocapnia. Thorax.

[62] Boldrini R, Fasano L, Nava S (2012). Noninvasive mechanical ventilation. Curr Opin Crit Care.

[63] Rialp Cervera G, del Castillo Blanco A, Pérez Aizcorreta O, Parra Morais L, GT-IRA of SEMICYUC (2014). Noninvasive mechanical ventilation in chronic
obstructive pulmonary disease and in acute cardiogenic pulmonary
edema. Med Intensiva.

[64] Brunner ME, Lyazidi A, Richard JC, Brochard L (2012). Non-invasive ventilation: indication for acute
respiratory failure. Rev Med Suisse.

[65] Agarwal R, Aggarwal AN, Gupta D, Jindal SK (2005). Non-invasive ventilation in acute cardiogenic pulmonary
oedema. Postgrad Med J..

[66] Ursella S, Mazzone M, Portale G, Conti G, Antonelli M, Gentiloni Silveri N (2007). The use of non-invasive ventilation in the treatment of
acute cardiogenic pulmonary edema. Eur Rev Med Pharmacol Sci.

[67] Peter JV, Moran JL, Phillips-Hughes J, Graham P, Bersten AD (2006). Effect of non-invasive positive pressure ventilation
(NIPPV) on mortality in patients with acute cardiogenic pulmonary oedema: a
meta-analysis. Lancet.

[68] Gray A, Goodacre S, Newby DE, Masson M, Sampson F, Nicholl J, 3CPO Trialists (2008). Noninvasive ventilation in acute cardiogenic pulmonary
edema. N Engl J Med.

[69] Pinsky MR (2005). Cardiovascular issues in respiratory
care. Chest.

[70] Wiesen J, Ornstein M, Tonelli AR, Menon V, Ashton RW (2013). State of the evidence: mechanical ventilation with PEEP
in patients with cardiogenic shock. Heart.

[71] Kushimoto S, Endo T, Yamanouchi S, Sakamoto T, Ishikura H, Kitazawa Y, Taira Y, Okuchi K, Tagami T, Watanabe A, Yamaguchi J, Yoshikawa K, Sugita M, Kase Y, Kanemura T, Takahashi H, Kuroki Y, Izumino H, Rinka H, Seo R, Takatori M, Kaneko T, Nakamura T, Irahara T, Saito N, the PiCCO Pulmonary Edema Study Group (2013). Relationship between extravascular lung water and
severity categories of acute respiratory distress syndrome by the Berlin
definition. Crit Care.

[72] Mekontso Dessap A, Roche-Campo F, Kouatchet A, Tomicic V, Beduneau G, Sonneville R (2012). Natriuretic peptide-driven fluid management during
ventilator weaning: a randomized controlled trial. Am J Respir Crit Care Med.

[73] American Thoracic Society;European Respiratory Society. American Thoracic
Society/European Respiratory Society International Multidisciplinary Consensus
Classification of the Idiopathic Interstitial Pneumonias (2002). This joint statement of the American Thoracic Society
(ATS), and the European Respiratory Society (ERS) was adopted by the ATS board of
directors, June 2001 and by the ERS Executive Committee, June 2001. Am J Respir Crit Care Med.

[74] Baldi BG, Pereira CA (2012). Diretrizes de Doenças Pulmonares Intersticiais da
Sociedade Brasileira de Pneumologia e Tisiologia. J Bras Pneumol.

[75] Mollica C, Paone G, Conti V, Ceccarelli D, Schmid G, Mattia P (2010). Mechanical ventilation in patients with end-stage
idiopathic pulmonary fibrosis. Respiration.

[76] Fernández-Pérez ER, Yilmaz M, Jenad H, Daniels CE, Ryu JH, Hubmayr RD (2008). Ventilator settings and outcome of respiratory failure
in chronic interstitial lung disease. Chest.

[77] Collard HR, Moore BB, Flaherty KR, Brown KK, Kaner RJ, King TE Jr, Lasky JA, Loyd JE, Noth I, Olman MA, Raghu G, Roman J, Ryu JH, Zisman DA, Hunninghake GW, Colby TV, Egan JJ, Hansell DM, Johkoh T, Kaminski N, Kim DS, Kondoh Y, Lynch DA, Müller-Quernheim J, Myers JL, Nicholson AG, Selman M, Toews GB, Wells AU, Martinez FJ (2007). Idiopathic Pulmonary Fibrosis Clinical Research Network
Investigators. Acute exacerbations of idiopathic pulmonary
fibrosis. Am J Respir Crit Care Med..

[78] Hyzy R, Huang S, Myers J, Flaherty K, Martinez F (2007). Acute exacerbation of idiopathic pulmonary
fibrosis. Chest.

[79] Suh GY, Kang EH, Chung MP, Lee KS, Han J, Kitaichi M (2006). Early intervention can improve clinical outcome of acute
interstitial pneumonia. Chest.

[80] Park IN, Kim DS, Shim TS, Lim CM, Lee SD, Koh Y (2007). Acute exacerbation of interstitial pneumonia other than
idiopathic pulmonary fibrosis. Chest.

[81] Yokoyama T, Kondoh Y, Taniguchi H, Kataoka K, Kato K, Nishiyama O (2010). Noninvasive ventilation in acute exacerbation of
idiopathic pulmonary fibrosis. Intern Med..

[82] Yokoyama T, Tsushima K, Yamamoto H, Koizumi T, Kubo K (2012). Potential benefits of early continuous positive pressure
ventilation in patients with rapidly progressive interstitial
pneumonia. Respirology.

[83] Al-Hameed FM, Sharma S (2004). Outcome of patients admitted to the intensive care unit
for acute exacerbation of idiopathic pulmonary fibrosis. Can Respir J..

[84] Mallick S (2008). Outcome of patients with idiopathic pulmonary fibrosis
(IPF) ventilated in intensive care unit. Respir Med.

[85] Brochard L, Rauss A, Benito S, Conti G, Mancebo J, Rekik N (1994). Comparison of three methods of gradual withdrawal from
ventilatory support during weaning from mechanical ventilation. Am J Respir Crit Care Med.

[86] Esteban A, Frutos F, Tobin MJ, Alía I, Solsona JF, Valverdú I (1995). A comparison of four methods of weaning patients from
mechanical ventilation. Spanish Lung Failure Collaborative Group. N Engl J Med.

[87] Epstein SK (2002). Decision to extubate. Intensive Care Med.

[88] MacIntyre NR, Cook DJ, Ely EW Jr, Epstein SK, Fink JB, Heffner JE, Hess D, Hubmayer RD, Scheinhorn DJ, American College of Chest Physicians, American Association for Respiratory Care, American College of Critical Care Medicine (2001). Evidence-based guidelines for weaning and discontinuing
ventilatory support: a collective task force facilitated by the American College
of Chest Physicians; the American Association for Respiratory Care; and the
American College of Critical Care Medicine. Chest.

[89] Esteban A, Alia I (1998). Clinical management of weaning from mechanical
ventilation. Intensive Care Med.

[90] Ely EW, Baker AM, Dunagan DP, Burke HL, Smith AC, Kelly PT (1996). Effect on the duration of mechanical ventilation of
identifying patients capable of breathing spontaneously. N Engl J Med.

[91] Kollef MH, Shapiro SD, Silver P, St John RE, Prentice D, Sauer S (1997). A randomized, controlled trial of protocol-directed
versus physician-directed weaning from mechanical ventilation. Crit Care Med.

[92] Marelich GP, Murin S, Battistella F, Inciardi J, Vierra T, Roby M (2000). Protocol weaning of mechanical ventilation in medical
and surgical patients by respiratory care practitioners and nurses: effect on
weaning time and incidence of ventilator-associated pneumonia. Chest.

[93] Navalesi P, Frigerio P, Moretti MP, Sommariva M, Vesconi S, Baiardi P (2008). Rate of reintubation in mechanically ventilated
neurosurgical and neurologic patients: evaluation of a systemic approach to
weaning and extubation. Crit Care Med.

[94] Blackwood B, Alderdice F, Burns K, Cardwell C, Lavery G, O'Halloran P (2011). Use of weaning protocols for reducing duration of
mechanical ventilation in critically ill adult patients: Cochrane systematic
review and meta-analysis. BMJ.

[95] Kress JP, Pohlman AS, O'Connor MF, Hall JB (2000). Daily interruption of sedative infusions in critically
ill patients undergoing mechanical ventilation. N Engl J Med.

[96] Goldwasser R, Farias A, Freitas EE, Saddy F, Amado V, Okamoto V (2007). Desmame e interrupção da ventilação
mecânica. J Bras Pneumol.

[97] Yang KL, Tobin MJ (1991). A prospective study of indexes predicting the outcome of
trials of weaning from mechanical ventilation. N Engl J Med.

[98] Nemer SN, Barbas CS, Caldeira JB, Cárias TC, Santos RG, Almeida LC (2009). A new integrative weaning index of discontinuation from
mechanical ventilation. Crit Care.

[99] Azeredo LM, Nemer SN, Caldeira JB, Guimaraes B, Noé R, Caldas CP (2011). Applying a new weaning index in ICU older
patients. Crit Care.

[100] Esteban A, Alía I, Gordo F, Fernández R, Solsona JF, Vallverdú I (1997). Extubation outcome after spontaneous breathing trials
with T-tube or pressure support ventilation. Am J Respir Crit Care Med.

[101] Esteban A, Alía I, Tobin MJ, Gil A, Gordo F, Vallverdú I (1999). Effect of spontaneous breathing trial duration on
outcome of attempts to discontinue mechanical ventilation. Am J Respir Crit Care Med.

[102] Perren A, Domenighetti G, Mauri S, Genini F, Vizzardi N (2002). Protocol-directed weaning from mechanical ventilation:
clinical outcome in patients randomized for a 30-min or 120-min trial with
pressure support ventilation. Intensive Care Med.

[103] Salam A, Tilluckdharry L, Amoateng-Adjepong Y, Manthous CA (2004). Neurologic status, cough, secretions and extubation
outcomes. Intensive Care Med.

[104] Zhou T, Zhang HP, Chen WW, Xiong ZY, Fan T, Fu JJ (2011). Cuff-leak test for predicting postextubation airway
complications: a systematic review. J Evid Based Med.

[105] Jaber S, Jung B, Chanques G, Bonnet F, Marret E (2009). Effects of steroids on reintubation and post-extubation
stridor in adults: meta-analysis of randomised controlled trials. Crit Care.

[106] Zhu F, Liu ZL, Long X, Wu XD, Zhou J, Bai CX (2013). Effect of noninvasive positive pressure ventilation on
weaning success in patients receiving invasive mechanical ventilation: a
meta-analysis. Chin Med J (Engl).

[107] Nava S, Gregoretti C, Fanfulla F, Squadrone E, Grassi M, Carlucci A (2005). Noninvasive ventilation to prevent respiratory failure
after extubation in high-risk patients. Crit Care Med.

[108] Ferrer M, Valencia M, Nicolas JM, Bernadich O, Badia JR, Torres A (2006). Early noninvasive ventilation averts extubation failure
in patients at risk: a randomized trial. Am J Respir Crit Care Med.

[109] Keenan SP, Powers C, McCormack DG, Block G (2002). Noninvasive positive-pressure ventilation for
postextubation respiratory distress: a randomized controlled trial. JAMA.

[110] Esteban A, Frutos-Vivar F, Ferguson ND, Arabi Y, Apezteguía C, González M (2004). Noninvasive positive-pressure ventilation for
respiratory failure after extubation. N Engl J Med.

[111] Ornico SR, Lobo SM, Sanches HS, Deberaldini M, Tófoli LT, Vidal AM (2013). Noninvasive ventilation immediately after extubation
improves weaning outcome after acute respiratory failure: a randomized controlled
trial. Crit Care.

[112] Glossop AJ, Shephard N, Bryden DC, Mills GH (2012). Non-invasive ventilation for weaning, avoiding
reintubation after extubation and in the postoperative period: a
meta-analysis. Br J Anaesth.

[113] Esen F, Denkel T, Telci L, Kesecioglu J, Tütüncü AS, Akpir K (1992). Comparison of pressure support ventilation (PSV) and
intermittent mandatory ventilation (IMV) during weaning in patients with acute
respiratory failure. Adv Exp Med Biol.

[114] White AC (2012). Long-term mechanical ventilation: management
strategies. Respir Care.

[115] MacIntyre NR, Epstein SK, Carson S, Scheinhorn D, Christopher K, Muldoon S (2005). National Association for Medical Direction of
Respiratory Care. Management of patients requiring prolonged mechanical
ventilation: report of a NAMDRC consensus conference. Chest.

[116] Scheinhorn DJ, Hassenpflug MS, Votto JJ, Chao DC, Epstein SK, Doig GS, Knight EB, Petrak RA, Ventilation Outcomes Study Group (2007). Post-ICU mechanical ventilation at 23 long-term care
hospitals: a multicenter outcomes study. Chest.

[117] Morandi A, Brummel NE, Ely EW (2011). Sedation, delirium and mechanical ventilation: the
'ABCDE' approach. Curr Opin Crit Care.

[118] Jubran A, Lawm G, Kelly J, Duffner LA, Gungor G, Collins EG (2010). Depressive disorders during weaning from prolonged
mechanical ventilation. Intens Care Med.

[119] Porhomayon J, Papadakos P, Nader ND (2012). Failed weaning from mechanical ventilation and cardiac
dysfunction. Crit Care Res Pract.

[120] McConville JF, Kress JP (2012). Weaning patients from the ventilator. N Eng J Med.

[121] Martin AD, Smith BK, Davenport PD, Harman E, Gonzalez-Rothi RJ, Baz M (2011). Inspiratory muscle strenght training improves weaning
outcome in failure to wean patients: a randomized trial. Crit Care.

[122] Daniel Martin A, Smith BK, Gabrielli A (2013). Mechanical ventilation, diaphragm weakness and weaning:
a rehabilitation perspective. Respir Physiol Neurobiol.

[123] Jubran A, Grant BJ, Duffner LA, Collins EG, Lanuza DM, Hoffman LA (2013). Effect of pressure support vs unassisted breathing
through a tracheostomy collar on weaning duration in patients requiring prolonged
mechanical ventilation: a randomized trial. JAMA.

[124] MacIntyre NR (2012). Evidence-based assessments in the ventilator
descontinuation process. Respir Care.

[125] Garnacho-Montero J, Amaya-Villar R, García-Garmendía JL, Madrazo-Osuna J, Ortiz-Leyba C (2005). Effect of critical illness polyneuropathy on the
withdrawal from mechanical ventilation and the lenght of stay in septic
patients. Crit Care Med.

[126] Hannan LM, Tan S, Hopkinson K, Marchingo E, Rautela L, Detering K (2013). Inpatient and long-term outcomes of individuals admitted
for weaning from mechanical ventilation at a specialized ventilation weaning
unit. Respirology.

[127] Pelosi P, Croci M, Ravagnan I, Cerisara M, Vicardi P, Lissoni A (1997). Respiratory system mechanics in sedated, paralyzed,
morbidly obese patients. J Appl Physiol.

[128] Burns SM, Egloff MB, Ryan B, Carpenter R, Burns JE (1994). Effect of body position on spontaneous respiratory rate
and tidal volume in patients with obesity, abdominal distension and
ascites. Am J Crit Care.

[129] Llano-Diez M, Renaud G, Andersson M, Marrero HG, Cacciani N, Engquist H (2012). Mechanisms underlying ICU muscle wasting and effects of
passive mechanical loading. Crit Care.

[130] Gosselink R, Bott J, Johnson M, Dean E, Nava S, Norrenberg M (2008). Physiotherapy for adult patients with critical illness:
recommendations of the European Respiratory Society and European Society of
Intensive Care Medicine Task Force on Physiotherapy for Critically Ill
Patients. Intensive Care Med..

[131] Rivers E, Nguyen B, Havstad S, Ressler J, Muzzin A, Knoblich B, Peterson E, Tomlanovich M, Early Goal-Directed Therapy Collaborative Group (2001). Early goal-directed therapy in the treatment of severe
sepsis and septic shock. N Engl J Med..

[132] Jones AE, Shapiro NI, Trzeciak S, Arnold RC, Claremont HA, Kline JA, Emergency Medicine Shock Research Network (EMShockNet)
Investigators (2010). Lactate clearance vs central venous oxygen saturation as
goals of early sepsis therapy: a randomized clinical trial. JAMA.

[133] Jansen TC, van Bommel J, Schoonderbeek J, Sleeswijk Visser SJ, van der Klooster JM, Lima AP, Willemsen SP, Bakker J, LACTATE study group (2010). Early lactate-guided therapy in intensive care unit
patients: a multicenter, open-label, randomized, controlled trial. Am J Respir Crit Care Med.

[134] Friedman G, De Backer D, Shahla M, Vincent JL (1998). Oxygen supply dependency can characterize septic
shock. Intensive Care Med.

[135] Wiedemann HP, Wheeler AP, Bernard GR, Thompson BT, Hayden D, deBoisblanc B, National Heart, Lung, and Blood Institute Acute Respiratory Distress
Syndrome (ARDS) Clinical Trials Network (2006). Comparison of two fluid-management strategies in acute
lung injury. N Engl J Med.

[136] Vieillard-Baron A, Schmitt JM, Augarde R, Fellahi JL, Prin S, Page B (2001). Acute cor pulmonale in acute respiratory distress
syndrome submitted to protective ventilation: incidence, clinical implications,
and prognosis. Crit Care Med.

[137] Osman D, Monnet X, Castelain V, Anguel N, Warszawski J, Teboul JL, Richard C, French Pulmonary Artery Catheter Study Group (2009). Incidence and prognostic value of right ventricular
failure in acute respiratory distress syndrome. Intensive Care Med.

[138] Phillips CR, Chesnutt MS, Smith SM (2008). Extravascular lung water in sepsis-associated acute
respiratory distress syndrome: indexing with predicted body weight improves
correlation with severity of illness and survival. Crit Care Med..

[139] Zhang Z, Lu B, Ni H. (2012). Prognostic value of extravascular lung water index in
critically ill patients: a systematic review of the literature. J Crit Care.

[140] D'Oliveira M, Sykes MK, Chakrabarti MK, Orchard C, Keslin J (1981). Depression of hypoxic pulmonary vasoconstriction by
sodium nitroprusside and nitroglycerine. Br J Anaesth.

[141] Zampieri FG, Park M, Azevedo LC, Amato MB, Costa EL (2012). Effects of arterial oxygen tension and cardiac output on
venous saturation: a mathematical modeling approach. Clinics (Sao Paulo).

[142] Yancy CW, Jessup M, Bozkurt B, Butler J, Casey DE Jr, Drazner MH (2013). 2013 ACCF/AHA guideline for the management of heart
failure: executive summary: a report of the American College of Cardiology
Foundation/American Heart Association Task Force on practice
guidelines. Circulation.

[143] Kushner FG, Hand M, Smith SC Jr, King SB 3rd, Anderson JL, Antman EM (2009). 2009 focused updates: ACC/AHA guidelines for the
management of patients with ST-elevation myocardial infarction (updating the 2004
guideline and 2007 focused update) and ACC/AHA/SCAI guidelines on percutaneous
coronary intervention (updating the 2005 guideline and 2007 focused update) a
report of the American College of Cardiology Foundation/American Heart Association
Task Force on Practice Guidelines. J Am Coll Cardiol.

[144] Jardin F, Farcot JC, Boisante L, Curien N, Margairaz A, Bourdarias JP (1981). Influence of positive end-expiratory pressure on left
ventricular performance. N Engl J Med.

[145] Hata K, Goto Y, Kawaguchi O, Takasago T, Saeki A, Nishioka T (1994). Hypercapnic acidosis increases oxygen cost of
contractility in the dog left ventricle. Am J Physiol..

[146] Rowe PA, Rocker GM, Burden RP (1988). Treatment of diuretic resistant cor pulmonale by
continuous arteriovenous haemofiltration. Thorax..

[147] Roberts JD, Forfia PR (2011). Diagnosis and assessment of pulmonary vascular disease
by Doppler echocardiography. Pulm Circ..

[148] De Backer D, Fagnoul D, Herpain A (2013). The role of invasive techniques in cardiopulmonary
evaluation. Curr Opin Crit Care.

[149] Zamanian RT, Haddad F, Doyle RL, Weinacker AB (2007). Management strategies for patients with pulmonary
hypertension in the intensive care unit. Crit Care Med..

[150] Carvalho CR, Barbas CS, Medeiros DM, Magaldi RB, Lorenzi G, Kairalla RA (1997). Temporal hemodynamic effects of permissive hypercapnia
associated with ideal PEEP in ARDS. Am J Respir Crit Care Med..

[151] Bhorade S, Christenson J, O'Connor M, Lavoie A, Pohlman A, Hall JB (1999). Response to inhaled nitric oxide in patients with acute
right heart syndrome. Am J Respir Crit Care Med..

[152] Christenson J, Lavoie A, O'Connor M, Bhorade S, Pohlman A, Hall JB (2000). The incidence and pathogenesis of cardiopulmonary
deterioration after abrupt withdrawal of inhaled nitric oxide. Am J Respir Crit Care Med..

[153] Michard F, Boussat S, Chemla D, Anguel N, Mercat A, Lecarpentier Y (2000). Relation between respiratory changes in arterial pulse
pressure and fluid responsiveness in septic patients with acute circulatory
failure. Am J Respir Crit Care Med..

[154] da Silva Ramos FJ, de Oliveira EM, Park M, Schettino GP, Azevedo LC (2011). Heart-lung interactions with different ventilatory
settings during acute lung injury and hypovolaemia: an experimental
study. Br J Anaesth.

[155] Monnet X, Osman D, Ridel C, Lamia B, Richard C, Teboul JL (2009). Predicting volume responsiveness by using the
end-expiratory occlusion in mechanically ventilated intensive care unit
patients. Crit Care Med..

[156] Magder S, Georgiadis G, Cheong T (1992). Respiratory variations in right atrial pressure predict
response to fluid challenge. J Crit Care.

[157] Monnet X, Rienzo M, Osman D, Anguel N, Richard C, Pinsky MR (2006). Passive leg raising predicts fluid responsiveness in the
critically ill. Crit Care Med..

[158] Vincent JL. (2011). "Let's give some fluid and see what happens" versus the
"mini-fluid challenge". Anesthesiology.

[159] DeVita MA, Spierer-Rundback L (1990). Swallowing disorders in patients with prolonged
orotracheal intubation or thacheostomy tubes. Crit Care Med.

[160] Elpern EH, Scott MG, Petro L, Ries MH (1994). Pulmonary aspiration in mechanically ventilated patients
with tracheostomies. Chest..

[161] Leder SB (2002). Incidence and type of aspiration in acute care patients
requiring mechanical ventilation via a new tracheostomy. Chest..

[162] Davis LA, Thompson Stanton S (2004). Characteristics of dysphagia in elderly patients
requiring mechanical ventilation. Dysphagia.

[163] Barker J, Martino R, Reichardt B, Hickey E, Ralph-Edwards A (2009). Incidence and impact of dysphagia in patients receiving
prolonged endotracheal intubation after cardiac surgery. Can J Surg..

[164] Skoretz SA, Flowers HL, Martino R (2010). The incidence of dysphagia following endotracheal
intubation: a systematic review. Chest..

[165] Macht M, King CJ, Wimbish T, Clark BJ, Benson AB, Burnham EL (2013). Post-extubation dysphagia is associated with longer
hospitalization in survivors of critical illness with neurologic
impairment. Crit Care..

[166] de Larminat V, Montravers P, Dureuil B, Desmonts JM (1995). Alteration in swallowing reflex after extubation in
intensive care unit patients. Crit Care Med..

[167] Ajemian MS, Nirmul GB, Anderson MT, Zirlen DM, Kwasnik EM (2001). Routine fiberoptic endoscopic evaluation of swallowing
following prolonged intubation: implications for management. Arch Surg..

[168] El Solh A, Okada M, Bhat A, Pietrantoni C (2003). Swallowing disorders post orotracheal intubation in the
elderly. Intensive Care Med..

[169] Keeling WB, Lewis V, Blazick E, Maxey TS, Garrett JR, Sommers KE (2007). Routine evaluation for aspiration after thoracotomy for
pulmonary resection. Ann Thorac Surg..

[170] Barquist E, Brown M, Cohn S, Lundy D, Jackowsky J. (2001). Postextubation fiberoptic endoscopic evaluation of
swallowing after prolonged endotracheal intubation: a randomized, prospective
trial. Crit Care Med..

[171] Leder SB, Cohn SM, Moller BA (1998). Fiberoptic endoscopic documentation of the high
incidence of aspiration following extubation in critically ill trauma
patients. Dysphagia..

[172] Mangilli LD, Moraes DP, Medeiros GC, Andrade CR, Limongi SC (2011). Protocolo de avaliação fonoaudiológica
preliminar. Disfagia: prática baseada em evidências.

[173] American Speech-Language-Hearing Association (2000). Clinical indicators for instrumental assessment of
dysphagia.

[174] Suiter DM, McCullough GH, Powell PW (2003). Effects of cuff deflation and one-way tracheostomy
speaking valve placement on swallow physiology. Dysphagia..

[175] Dikeman KJ, Kazandjian MS (1995). Communication and swallowing management of tracheostomized and
ventilator dependent adults.

[176] Donzelli J, Brady S, Wesling M, Craney M (2001). Simultaneous modified Evans blue dye procedure and video
nasal endoscopic evaluation of the swallow. Laryngoscope..

[177] O'Neil-Pirozzi TM, Lisiecki DJ, Jack Momose K, Connors JJ, Milliner MP (2003). Simultaneous modified barium swallow and blue dye tests:
a determination of the accuracy of blue dye test aspiration
findings. Dysphagia.

[178] Belafsky PC, Blumenfeld L, LePage A, Nahrstedt K (2003). The accuracy of the modified Evan's blue dye test in
predicting aspiration. Laryngoscope.

[179] Warms T, Richards J (2000). "Wet voice" as a predictor of penetration and aspiration
in oropharyngeal dysphagia. Dysphagia.

[180] Wu MC, Chang YC, Wang TG, Lin LC (2004). Evaluating swallowing dysfunction using a 100-ml water
swallowing test. Dysphagia.

[181] Woisard V, Réhault E, Brouard C, Fichaux-Bourin P, Puech M, Grand S. (2009). Study of the predictive value of detection tests for
silent aspirations. Rev Laryngol Otol Rhinol (Bord).

[182] Santoro PP, Barros AP, Dedivitis RA, Santana RB (2012). Tratamento medicamentoso da sialorreia. Deglutição, voz e fala nas alterações neurológicas.

[183] Furkim AM, Silva RG, Furkim AM, Silva RG (1999). Procedimentos fonoaudiológicos. Programas de reabilitação em disfagia neurogênica.

[184] Kola A, Eckmanns T, Gastmeier P (2005). Efficacy of heat and moisture exchangers in preventing
ventilator-associated pneumonia: meta-analysis of randomized controlled
trials. Intensive Care Med..

[185] Lorente L, Lecuona M, Galván R, Ramos MJ, Mora ML, Sierra A. (2004). Periodically changing ventilator circuits is not
necessary to prevent ventilator-associated pneumonia when a heat and moisture
exchanger is used. Infect Control Hosp Epidemiol..

[186] Samransamruajkit R, Jirapaiboonsuk S, Siritantiwat S, Tungsrijitdee O, Deerojanawong J, Sritippayawan S (2010). Effect of frequency of ventilator circuit changes (3 vs
7 days) on the rate of ventilator-associated pneumonia in PICU. J Crit Care..

[187] Rutala WA, Gergen MF, Weber DJ (2008). Impact of an oil-based lubricant on the effectiveness of
the sterilization processes. Infect Control Hosp Epidemiol..

[188] Happ MB, Tate JA, Swigart VA, DiVirgilio-Thomas D, Hoffman LA (2010). Wash and wean: bathing patients undergoing weaning
trials during prolonged mechanical ventilation. Heart Lung..

[189] Hodgson CL, Berney S, Harrold M, Saxena M, Bellomo R (2013). Clinical review: Early patient mobilization in the
ICU. Crit Care..

[190] Winkelman C, Chiang LC. (2010). Manual turns in patients receiving mechanical
ventilation. Crit Care Nurse..

[191] Metheny NA, Frantz RA (2013). Head-of-bed elevation in critically ill patients: a
review. Crit Care Nurse..

[192] Castellões TM, da Silva LD (2009). Ações de enfermagem para a prevenção da extubação
acidental. Rev Bras Enferm..

[193] Caraviello KA, Nemeth LS, Dumas BP (2010). Using the beach chair position in ICU
patients. Crit Care Nurse..

[194] Guérin C, Reignier J, Richard JC, Beuret P, Gacouin A, Boulain T, Mercier E, Badet M, Mercat A, Baudin O, Clavel M, Chatellier D, Jaber S, Rosselli S, Mancebo J, Sirodot M, Hilbert G, Bengler C, Richecoeur J, Gainnier M, Bayle F, Bourdin G, Leray V, Girard R, Baboi L, Ayzac L, PROSEVA Study Group (2013). Prone positioning in severe acute respiratory distress
syndrome. N Engl J Med..

[195] Roche-Campo F, Aguirre-Bermeo H, Mancebo J (2011). Prone postioning in acute respiratory distress syndrome
(ARDS): when and how?. Presse Med..

[196] Jelic S, Cunningham JA, Factor P (2008). Clinical review: airway hygiene in the intensive care
unit. Crit Care..

[197] Dong L, Yu T, Yang Y, Qiu HB (2012). The effects and safety of closed versus open tracheal
suction system: a meta analysis. Zhonghua Nei Ke Za Zhi..

[198] Associação de Medicina Intensiva Brasileira (AMIB).Departamento de
Odontologia e Departamento de Enfermagem Recomendações para higiene bucal do paciente adulto em UTI -
AMIB.

[199] Vieira DF (2009). Implantação de protocolo de prevenção da pneumonia associada à
ventilação mecânica: impacto do cuidado não farmacológico.

[200] McClave SA, Martindale RG, Vanek VW, McCarthy M, Roberts P, Taylor B, Ochoa JB, Napolitano L, Cresci G, A.S.P.E.N. Board of Directors (2009). American College of Critical Care Medicine; Society of
Critical Care Medicine. Guidelines for the Provision and Assessment of Nutrition
Support Therapy in the Adult Critically Ill Patient: Society of Critical Care
Medicine (SCCM) and American Society for Parenteral and Enteral Nutrition
(A.S.P.E.N.). JPEN J Parenter Enteral Nutr..

[201] Seder CW, Stockdale W, Hale L, Janczyk RJ (2010). Nasal bridling decreases feeding tube dislodgment and
may increase caloric intake in the surgical intensive care unit: a randomized,
controlled trial. Crit Care Med..

[202] Silva MA, Santos Sda G, Tomasi CD, Luz Gd, Paula MM, Pizzol FD (2013). Enteral nutrition discontinuation and outcomes in
general critically ill patients. Clinics (Sao Paulo).

[203] Dekker J, van Baar ME, Curfs EC, Kerssens JJ (1993). Diagnosis and treatment in physical therapy: an
invetigation of their relationship. Phys Ther..

[204] Castro AA, Calil SR, Freitas SA, Oliveira AB, Porto EF (2013). Chest physiotherapy effectiveness to reduce
hospitalization and mechanical ventilation length of stay, pulmonary infection
rate and mortality in ICU patients. Respir Med..

[205] Lord RK, Mayhew CR, Korupolu R, Mantheiy EC, Friedman MA, Palmer JB (2013). ICU early physical rehabilitation programs: financial
modeling of cost savings. Crit Care Med..

[206] Stiller K. (2013). Physiotherapy in intensive care: an updated systematic
rewiew. Chest..

[207] França EET, Ferrari FR, Fernandes Patrícia V, Cavalcanti R, Duarte A, Aquim EE, Damasceno MCP Força tarefa sobre a fisioterapia em pacientes críticos
adultos: Diretrizes da Associação Brasileira de Fisioterapia Respiratória e
Terapia Intensiva (ASSOBRAFIR) e Associação de Medicina Intensiva Brasileira
(AMIB).

[208] Kralj A, Jaeger RJ, Munih M (1990). Analysis of standing up and sitting down in humans:
definitions and normative data presentation. J Biomech..

[209] Ellis G, Langhorne P (2005). Comprehensive geriatric asssesment for older hospital
patients. Br Med Bull..

[210] Heyland DK, Dhaliwal R, Drover JW, Gramlich L, Dodek P (2003). Canadian Critical Care Clinical Practice Guidelines
Committee. Canadian clinical practice guidelines for nutrition support in
mechanically ventilated, critically ill adults patients. JPEN J Parenter Enteral Nutr..

[211] Kreymann KG, Berger MM, Deutz NE, Hiesmayr M, Jolliet P, Kazandjiev G, Nitenberg G, van den Berghe G, Wernerman J, Ebner C, Hartl W, Heymann C, Spies C, DGEM (German Society for Nutritional Medicine), ESPEN (European Society for Parenteral and Enteral Nutrition) (2006). ESPEN guidelines on enteral nutrition: intensive
care. Clin Nutr..

[212] Frankenfield DC, Ashcraft CM (2011). Estimating energy needs in nutrition support
patients. JPEN J Parenter Enteral Nutr..

[213] Faisy C, Lerolle N, Dachraoui F, Savard JF, Abboud I, Tadie JM (2009). Impact of energy deficit calculated by a predictive
method on outcome in medical patients requiring prolonged acute mechanical
ventilation. Br J Nutr..

[214] Weijs PJ, Stapel SN, de Groot SD, Driessen RH, de Jong E, Girbes AR (2012). Optimal protein and energy nutrition decreases mortality
in mechanically ventilated, critically ill patients: a rospective observational
cohort study. JPEN J Parenter Enteral Nutr..

[215] Krenitsky J, Rosner MH (2011). Nutritional support for patients with acute kidney
injury: how much protein is enough or too much?. Pract Gastroenterol..

[216] Chen F, Wang J, Jiang Y (2011). Influence of different routes of nutrition on the
respiratory muscle strength and prognosis of elderly patients in respiratory
intensive care unit. Chinese J Clin Nutr..

[217] Marick PE, Zalog GP (2001). Early enteral nutrition in acutely ill patients: a
systematic rewiew. Crit Care Med..

[218] Doig GS, Heighes PT, Simpson F, Sweetman EA, Davies AR (2009). Early enteral nutrition, provided within 24 h of injury
or intensive care unit admission, significantly reduces mortality in critically
ill patients: a meta-analysis of randomised controlled trials. Intensive Care Medicine..

[219] Heyland DK, Drover JW, Dhaliwal R, Greenwood J. (2002). Optimizing the benefits and minimizing the risks of
enteral nutrition in the critically ill: role of small bowel
feeding. JPEN J Parenter Enteral Nutr..

[220] Alexiou VG, Ierodiakonou V, Dimopoulos G, Falagas ME (2009). Impact of patient position on the incidence of
ventilator-associated pneumonia: a meta-analysis of randomized controlled
trials. J Crit Care..

[221] Doley J, Mallampalli A, Sandberg M (2011). Nutrition management for the patient requiring prolonged
mechanical ventilation. Nutr Clin Pract..

[222] Poulard F, Dimet J, Martin-Lefevre L, Bontemps F, Fiancette M, Clementi E (2010). Impact of not measuring residual gastric volume in
mechanically ventilated patients receiving early enteral feeding: a prospective
before-after study. JPEN J Parenter Enteral Nutr..

[223] Reignier R, Mercier E, Le Gouge A, Boulain T, Desachy A, Bellec F, Clavel M, Frat JP, Plantefeve G, Quenot JP, Lascarrou JB, Clinical Research in Intensive Care and Sepsis (CRICS) Group (2013). Effect of not monitoring residual gastric volume on risk
of ventilator-associated pneumonia in adults receiving mechanical ventilation and
early enteral feeding: a randomized controlled trial. JAMA.

[224] Mackenzie SL, Zygun DA, Whitmore BL, Doig CJ, Hameed SM (2005). Implementation of a nutrition support protocol increases
the proportion of mechanically ventilated patients reaching enteral nutrition
targets in the adult intensive care unit. JPEN J Parenter Enteral Nutr..

[225] Talpers SS, Roberger DJ, Bunce SB, Pingleton SK (1992). Nutritionally associated increased carbon dioxide
production. Excess total calories vs high proportion of carbohydrate
calories. Chest..

[226] Singer P, Theilla M, Fisher H, Gibstein L, Grozovski E, Cohen J (2006). Benefit of an enteral diet enriched with
eicosapentaenoic acid and gamma-linolenic acid in ventilated patients with acute
lung injury. Crit Care Med..

[227] Grau-Carmona T, Morán-García V, García-de-Lorenzo A, Heras-de-la-Calle G, Quesada-Bellver B, López-Martínez J (2011). Effect of an enteral diet enriched with eicosapentaenoic
acid, gamma-linolenic acid and anti-oxidants on the outcome of mechanically
ventilated, critically ill, septic patients. Clin Nutr..

[228] Rice TW, Wheeler AP, Thompson BT, deBoisblanc BP, Steingrub J, Rock P, NIH NHLBI Acute Respiratory Distress Syndrome Network of
Investigators (2011). Enteral omega-3 fatty acid, gamma-linolenic acid, and
antioxidant supplementation in acute lung injury. JAMA.

[229] Pontes-Arruda A, Aragão AM, Albuquerque JD (2006). Effects of enteral feeding with eicosapentaenoic acid,
gamma-linolenic acid, and antioxidants in mechanically ventilated patients with
severe sepsis and septic shock. Crit Care Med..

[230] Alsumrain MH, Jawad SA, Imran NB, Riar S, DeBari VA, Adelman M. (2010). Association of hypophosphatemia with failure-to-wean
from mechanical ventilation. Ann Clin Lab Sci..

