# Supplementary material for: Brazilian recommendations of mechanical ventilation 2013. Part 2
Source: J Bras Pneumol. 2014 Sep-Oct;40(5):458–86. doi: 10.1590/S1806-37132014000500003 (PMC4263328; doi:10.1590/S1806-37132014000500003)
Supplement: Translation

# Translation: Recomendações brasileiras de ventilação mecânica 2013. Parte 2

**Keywords:** Recomendações, Ventilação Mecânica, Insuficiência Respiratória

## Abstract

O suporte ventilatório artificial invasivo e não invasivo ao paciente grave tem evoluído e inúmeras evidências têm surgido, podendo ter impacto na melhora da sobrevida e da qualidade do atendimento oferecido nas unidades de terapia intensiva no Brasil. Isto posto, a Associação de Medicina Intensiva Brasileira (AMIB) e a Sociedade Brasileira de Pneumologia e Tisiologia (SBPT) - representadas por seu Comitê de Ventilação Mecânica e sua Comissão de Terapia Intensiva, respectivamente, decidiram revisar a literatura e preparar recomendações sobre ventilação mecânica, objetivando oferecer aos associados um documento orientador das melhores práticas da ventilação mecânica na beira do leito, com base nas evidências existentes, sobre os 29 subtemas selecionados como mais relevantes no assunto. O projeto envolveu etapas que visaram distribuir os subtemas relevantes ao assunto entre *experts* indicados por ambas as sociedades, que tivessem publicações recentes no assunto e/ou atividades relevantes em ensino e pesquisa no Brasil, na área de ventilação mecânica. Esses profissionais, divididos por subtemas em duplas, responsabilizaram-se por fazer uma extensa revisão da literatura mundial. Reuniram-se todos no Fórum de Ventilação Mecânica, na sede da AMIB, na cidade de São Paulo (SP), em 3 e 4 de agosto de 2013, para finalização conjunta do texto de cada subtema e apresentação, apreciação, discussão e aprovação em plenária pelos 58 participantes, permitindo a elaboração de um documento final.

Essas recomendações são uma iniciativa conjunta do Comitê de Ventilação Mecânica da Associação de Medicina Intensiva Brasileira (AMIB) e da Comissão de Terapia Intensiva da Sociedade Brasileira de Pneumologia e Tisiologia (SBPT).

## Introdução

O suporte ventilatório mecânico, tanto não invasivo como invasivo, deve ser realizado de forma adequada e segura, para evitarmos a lesão induzida pela ventilação mecânica. A ventilação mecânica moderna e atual, guiada pelos conhecimentos de fisiologia e pelas evidências literárias, tanto dos experimentos de laboratório, como pelos ensaios clínicos randomizados e/ou observacionais com pacientes, indicam um suporte ventilatório com volumes correntes de 6mL/kg de peso predito; delta entre a pressão de platô (PPlatô) e a pressão expiratória final positiva (PEEP) de, no máximo, 15cmH_2_O; níveis de pressão expiratória final suficientes para evitar o colabamento das vias aéreas e dos alvéolos e garantir uma troca gasosa adequada; posicionamento dos pacientes no leito de maneira a garantir uma ventilação adequada e não lesiva (como a posição prona, nos casos de síndrome do desconforto respiratório agudo - SDRA grave); e técnicas de suporte avançado (como a circulação extracorpórea com remoção do gás carbônico (CO_2_) nos casos de SDRA refratária). Com o advento de ventiladores cada vez mais sofisticados e com a possibilidade de ajuste fino de sensibilidade e de diversos mecanismos de disparo, de diferentes velocidades e aceleração de fluxo inspiratório, diversos mecanismos de término de tempo inspiratório e diversas opções de monitorização, há a possibilidade de ajuste de sincronia do paciente com o ventilador mecânico e a ventilação mecânica de acordo com a doença respiratória apresentada pelos pacientes, destacando-se o suporte ventilatório direcionado para as doenças restritivas, diferentemente das obstrutivas.

Assim, fez-se a necessidade de reunião de especialistas brasileiros envolvidos com suporte ventilatório (anestesia, terapia intensiva, pneumologia, fisioterapia, enfermagem, nutrição e fonoaudiologia), para análise das evidências literárias disponíveis sobre ventilação mecânica que, associados à experiência das diversas especialidades, pudessem orientar os profissionais das unidades de terapias intensivas (UTI) brasileiras sobre como oferecer aos nossos pacientes com insuficiência respiratória um suporte respiratório seguro e eficaz, baseado nas melhores evidências disponíveis, para evitarmos a possibilidade de lesão associada à ventilação mecânica. 

Assim, o objetivo do presente trabalho foi rever a literatura disponível sobre 29 subtemas relacionados ao suporte ventilatório do paciente em insuficiência respiratória e, após apresentação, discussão e aprovação em plenária composta por 58 especialistas, apresentá-los sob a forma de recomendações ou sugestões. 

## Metodologia

A literatura disponível no MEDLINE (2003-2013) e na *Cochrane Central Register of Controlled Trials* (CENTRAL) foi revisada por especialistas com nível escolar superior (médicos - intensivistas, pneumologistas e anestesiologistas - fisioterapeutas, enfermeiros, nutricionistas e fonoaudiólogos), dispostos em duplas para cada um dos 29 subtemas escolhidos sobre suporte ventilatório não invasivo e invasivo em pacientes com insuficiência respiratória

Após a revisão dos artigos disponíveis na literatura, cada dupla respondeu as perguntas formuladas pela Comissão Organizadora, constituída pelos Drs. Carmen Sílvia Valente Barbas, Presidente do Comitê de Ventilação Mecânica da Associação de Medicina Intensiva Brasileira (AMIB), Alexandre Marini Ísola, Coordenador Nacional do Curso de Ventilação Mecânica para Adultos em UTI da AMIB (VENUTI) e Augusto Manoel de Carvalho Farias, Coordenador da Comissão de Terapia Intensiva da Sociedade Brasileira de Pneumologia e Tisiologia (SBPT), de acordo com os critérios previamente sugeridos por outros autores.^(^ 1 ^-^ 4 ^)^ Assim, usou-se o termo "recomendação" quando o nível de evidência era forte, com base em estudos randomizados com mais de cem pacientes, meta-análises e efeito tudo ou nada ou de segurança para os pacientes. O termo "sugestão" foi utilizado quando a evidência foi considerada fraca, ou seja, baseada em estudos observacionais, casos-controle, série de casos e ou experiência de especialistas para orientação do suporte ventilatório nacional de maneira eficaz e segura, evitando-se os possíveis efeitos deletérios associados ao suporte ventilatório não adequado para nossos pacientes.

Os 58 especialistas participantes responderam as perguntas propostas durante 8 horas de reunião na sede da AMIB, no dia 3 de agosto de 2013. Após a elaboração das respostas às perguntas formuladas baseadas nas evidências disponíveis na literatura e na experiência dos especialistas, estas foram apresentadas em plenária para todos os 58 especialistas presentes no dia 4 de agosto de 2013, na sede da AMIB, sendo discutidas, modificadas quando necessário, votadas e aprovadas, de acordo com sugestões e observações dos especialistas presentes.

Todos os relatórios entregues pelas duplas foram reunidos pela Comissão Organizadora do projeto, que revisou, formatou e produziu o documento final, após revisão dos autores. A seguir, o documento foi encaminhamento para impressão de manuais de beira de leito, contendo as recomendações para distribuição nas UTIs de todo o Brasil e para publicação na Revista Brasileira de Terapia Intensiva (RBTI) e no Jornal de Pneumologia. 

## Ventilação mecânica no trauma torácico

Ventilação mecânica não invasiva 

**Recomendação - **A ventilação não invasiva (VNI) está contraindicada nos pacientes com lesão das vias aéreas superiores, na presença de instabilidade hemodinâmica e no traumatismo craniencefálico (TCE) grave.^(^ 5 ^-^ 10 ^)^

**Recomendação - **Em pacientes com trauma torácico isolado, a aplicação precoce de VNI é capaz de melhorar as trocas gasosas, prevenir a intubação orotraqueal (IOT), reduzir o tempo de estadia na UTI e complicações.^(^ 5 ^-^ 10 ^)^

**Recomendação - **O uso de VNI deve ser monitorado por profissional da saúde à beira do leito de 0,5 a 2 horas. Para ser considerado sucesso, devem ser observados: diminuição da frequência respiratória (f), aumento do volume corrente (VC), melhora do nível de consciência, diminuição ou cessação de uso de musculatura acessória, aumento da pressão parcial de oxigênio (PaO_2_) e/ou da saturação periférica de oxigênio (SpO_2_) e diminuição da pressão parcial de gás carbônico (PaCO_2_), sem distensão abdominal significativa. Quando não há sucesso, recomendam-se imediata IOT e ventilação invasiva.

Ventilação mecânica invasiva(11)

**Recomendação - **Devem-se intubar e ventilar prontamente pacientes com trauma torácico grave, com insuficiência respiratória e contraindicações formais a VNI. 

**Recomendação - **Inicialmente, utilizar modo de ventilação tipo assistido-controlada, ventilação ciclada a volume (VCV) ou ventilação controlada à pressão (PCV), no trauma torácico com insuficiência respiratória grave. 

**Recomendação - **Independente da modalidade escolhida (VCV ou PCV), pacientes com trauma torácico devem ser ventilados inicialmente com VC 6mL/kg de peso predito, f entre 16-20 rpm e fração inspirada de oxigênio (FiO_2_) suficiente para manter uma SpO_2_>92% e PEEP entre 5 e 10cmH_2_O. Nos casos de SDRA, seguir as orientações constantes no tema específico dessas recomendações.

**Recomendação - **Nos casos de fístula broncopleural de alto débito, utilizar modo PCV, que compensará o vazamento. Outra opção é o uso da ventilação de alta frequência (HFOV), apenas nos centros com esse recurso e pessoal especializado. Nos casos mais graves, pode-se usar ventilação independente assíncrona ou não, ventilando-se o pulmão da fístula com modo PCV com pressão de distensão <15cmH_2_O e PEEP mais baixos. (<10cmH_2_O).

Controle da dor 

**Sugestão -** Recomenda-se analgesia epidural torácica dentro de uma estratégia multimodal. Na impossibilidade ou contraindicação da epidural, pode-se utilizar analgesia intravenosa (IV) controlada pelo paciente ou bloqueio dos nervos intercostais.^(^ 11 ^)^

**Sugestão -** Sugere-se que, em situações de dor menos intensa, a aplicação de analgesia intermitente possa ser utilizada. 

## Ventilação mecânica durante procedimentos cirúrgicos

**Comentário -** As complicações pulmonares pós-operatórias (CPP) contribuem para uma proporção substancial dos riscos associados à cirurgia e à anestesia, sendo as maiores causas de morbimortalidade no pós-operatório.^(^ 12 ^)^ As CPP, que compreendem infecções respiratórias, falência respiratória, derrame pleural, atelectasias, pneumotórax, broncoespasmo e pneumonite aspirativa, comprometem aproximadamente 5% dos pacientes submetidos à cirurgia.^(^ 12 ^,^ 13 ^)^

Cuidados específicos na pré-intubação

Estratificação de risco pré-operatório

**Recomendação - **Todos os pacientes devem ser avaliados quanto ao risco de CPP por meio de escala específica. A classificação de *American Society Anesthesiology* (ASA) é uma escala subjetiva e com baixa precisão. Entre as escalas sugeridas para a estratificação de risco dos pacientes quanto a CPP, encontram-se a *European Surgical Outcomes Study *(EuSOS) e a *Assess Respiratory Risk in Surgical Patients in Catalonia* (ARISCAT).^(^ 13 ^)^

Pré-oxigenação durante a indução anestésica

**Recomendação - **A pré-oxigenação com FiO_2_ ao redor de 100% para todos os pacientes visa aumentar a reserva e diminuir o risco de hipoxemia.^(^ 14 ^)^

Utilização de ventilação não invasiva durante a indução anestésica

**Sugestão -** Utilizar VNI durante a indução anestésica para cirurgias eletivas em pacientes com redução da complacência abdominal e naqueles em que é necessária a utilização de FiO_2_ de 100% para pré-oxigenação, sendo capaz de prevenir a formação de atelectasias em pacientes com redução da complacência abdominal e submetidos à pré-oxigenação com FiO_2_ de 100%.^(^ 14 ^)^

Utilização de pressão positiva ao final da expiração e manobra de recrutamento alveolar durante a indução anestésica

**Sugestão -** Utilizar manobras de recrutamento e PEEP em torno de 5 a 8cmH_2_O após a indução anestésica em pacientes com redução da complacência abdominal ou naqueles que apresentarem hipoxemia.^(^ 14 ^)^

Cuidados específicos durante o intraoperatório

Modalidade ventilatória

**Sugestão -** Utilizar ventilação com PCV em pacientes submetidos à cirurgia videolaparoscópica visando à melhor mecânica pulmonar. Observar adequado valor de VC expirado.^(15,16) ^No restante dos cenários, não existe benefício de um modo ventilatório em detrimento de outro, se respeitada a mecânica ventilatória.

Volume corrente

**Recomendação - **O emprego de ventilação mecânica com VC de 6mL/kg de peso predito deve ser considerado em todos os pacientes. Diversos estudos em diversos cenários (cirurgia abdominal, torácica e cardíaca) demonstraram o benefício da utilização de baixo VC durante cirurgia.^(^ 17 ^-^ 19 ^)^

Pressão positiva ao final da expiração 

**Sugestão -** A utilização de PEEP ao redor de 5 a 8cmH_2_O deve ser considerada. Todos os estudos avaliando estratégia convencional de ventilação mecânica *versus* estratégia protetora em pacientes submetidos à cirurgia consideraram, na estratégia protetora, a utilização de VC baixo e PEEP elevada. De forma geral, a utilização de PEEP mais elevada resulta em melhor oxigenação e em menor incidência de CPP.^(^ 17 ^,^ 20 ^)^

Manobras de recrutamento alveolar 

**Sugestão -** Utilizar manobra de recrutamento alveolar (MR) durante o intraoperatório para reverter colapso alveolar e melhorar a oxigenação. A utilização de RM, associada à manutenção de níveis mais elevados de PEEP, reduz a quantidade de pulmão colabado e melhora a oxigenação de pacientes durante a cirurgia.^(17,20) ^Entre as RM mais citadas na literatura, encontra-se a manobra de recrutamento máximo, que busca manter uma PPlatô mais alta na via aérea (ao redor de 40 a 45cmH_2_O) por 60 segundos (MRM, como descrito na primeira parte destas Recomendações).

Fração inspirada de oxigênio 

**Sugestão -** Deve-se utilizar a mais baixa FiO_2_ capaz de manter a SpO_2_ ao redor de 96 a 98%. A combinação de FiO_2_ ao redor de 30 a 40% e níveis mais elevados de PEEP é capaz de manter uma adequada oxigenação e reduzir a atelectasia pulmonar. O papel de altos níveis de oxigênio na prevenção da infecção de ferida operatória ainda é controverso.^(^ 14 ^)^

Frequência respiratória

**Recomendação - **Utilizar f visando manter PaCO_2_ entre 38 e 43mmHg (ETCO_2 _[CO_2_ exalado ao final da expiração] ao redor de 35 e 40mmHg). A tendência a utilização de VC mais baixo, durante a cirurgia, torna necessária a adoção de uma f mais elevada.^(^ 17 ^,^ 19 ^)^

Retirada da ventilação mecânica

**Recomendação - **Realizar a retirada da ventilação mecânica no pós-operatório o mais precoce e rapidamente possível, quando o paciente apresentar-se hemodinamicamente estável, com analgesia adequada, sem distúrbios hidroeletrolíticos e com nível de consciência suficiente para manutenção do *drive* ventilatório e proteção da via aérea. A extubação pode ser realizada na sala cirúrgica, na recuperação pós-anestésica ou na UTI.^(^ 21 ^)^

Ventilação não invasiva após a extubação

**Sugestão -** O uso da VNI deve ser considerado em pacientes submetidos à cirurgia cardíaca, torácica, bariátrica e abdominal alta, pois está associado à melhor oxigenação e à menor incidência de atelectasia. Deve ser realizado com níveis baixos de pressão em cirurgias abdominais alta e de esôfago. A VNI não deve retardar a reintubação. 

**Recomendação - **A VNI não deve ser usada na ocorrência de insuficiência respiratória aguda iniciada após a extubação.^(^ 22 ^)^

## Ventilação mecânica nos pacientes obesos

**Comentário -** Pacientes com índice de massa corporal (IMC) ≥30kg/m^2^ são considerados obesos. Essa condição cursa com uma série de alterações fisiológicas, como a redução da complacência pulmonar, decorrente do efeito mecânico direto da distribuição de gordura e da posição anormal do diafragma, devido ao aumento da pressão intra-abdominal (PIA), à redução da capacidade residual funcional (CRF) e à capacidade pulmonar total (CPT), e do aumento do trabalho respiratório, por aumento da resistência das vias aéreas (Rva) e da parede torácica, além da necessidade de alto volume minuto, levando à elevação de PaCO_2_.^(^ 23 ^,^ 24 ^)^

**Recomendação - **Considerar todo paciente obeso como potencial via aérea difícil (VAD). Nesses pacientes, se escalas de Malampatti ≥3 e Cormack 3-4, e aumento da circunferência cervical, considerar VAD e preparar infraestrutura para essa condição.^(^ 25 ^)^

**Sugestão -** Adotar a posição de Trendelemburg reverso durante a ventilação.^(^ 26 ^)^ O objetivo é melhorar a PaO_2_, a complacência estática do sistema respiratório (Csr) e o débito cardíaco (DC), além de reduzir a formação de atelectasias. 

**Sugestão -** Evitar a posição supina, em virtude da redução da capacidade residual funcional, do débito cardíaco e pelo aumento do trabalho respiratório. Na possibilidade da realização, sugere-se adotar a posição *beach chair* (cadeira de praia).^(^ 27 ^)^

**Sugestão -** Utilizar VNI em casos de insuficiência respiratória hipercápnica com os cuidados pertinentes à tecnica. A utilização em pacientes com IMC ≥45kg/m^2^ deve ser realizada com mais cuidado, em virtude do maior risco de falha nesse grupo.

**Sugestão -** Na ventilação mecânica invasiva, não há superioridade entre os modos. Sugere-se inicialmente usar modo assistido/controlado (A/C) PCV ou VCV.^(^ 28 ^)^

**Sugestão -** Realizar a monitorização da mecânica respiratória. A monitorização da PIA deve ser avaliada em casos de aumento dos níveis de PaCO_2_ e/ou aumento das pressões nas vias aéreas, que não possa ser justificado por causas pulmonares.

**Recomendação - **Para VC, 6mL/kg de peso predito,^(26-29) ^para FiO_2_, sugere-se manter o mínimo valor para manter saturação de oxigênio (SatO_2_) ≥92%.

**Sugestão -** Para PEEP/manobras de recrutamento,^(^ 30 ^,^ 31 ^)^ o objetivo é aumentar a CRF, prevenir a formação de atelectasias e reduzir o risco de VILI. Sugere-se também realizar manobras de MRM nos casos de hipoxemia, diminuição do VC ou níveis elevados de PaCO_2_. 

**Sugestão -** Utilizar níveis de PEEP ≥10cmH_2_O.

**Recomendação - **Limitar PPlatô ≤ 35cmH_2_O.^(^ 32 ^)^

**Sugestão -** Em casos de SARA moderada e grave, tolera-se subir a PPlatô até um máximo de 40cmH_2_O, mantendo-se uma Pdistensão ≤15cmH_2_O, necessariamente.

**Recomendação - **Extubar assim que as condições clínicas permitirem, podendo-se utilizar VNI para facilitação.

## Ventilação mecânica nos pacientes com acometimento do sistema nervoso central

Troca gasosa - oxigênio 

**Recomendação - **Evitar hipoxemia em pacientes com lesão neurológica aguda, uma vez que leva ao aumento na morbidade e taxa de mortalidade.^(^ 33 ^,^ 34 ^)^

**Sugestão -** Evitar hiperóxia em casos de encefalopatia anóxico-isquêmica.^(^ 35 ^)^

Troca gasosa - gás carbônico 

**Recomendação - **Não utilizar hiperventilação profilática ou prolongada, e manter PaCO_2_ entre 35 e 40mmHg na fase aguda da injúria.^(^ 36 ^-^ 38 ^)^

**Recomendação - **Indicar hiperventilação aguda em casos de herniação cerebral como tratamento de resgate.^(^ 36 ^-^ 38 ^)^

**Recomendação - **Monitorização de CO_2_ por capnografia. Na ausência desta, checar PaCO_2_ em gasometria com maior frequência na fase aguda.

**Sugestão -** Nos pacientes com acidente vascular cerebral (AVC) isquêmico agudo, evitar PaCO_2_<35mmHg por risco de isquemia na área de penumbra. 

Sindrome do desconforto respiratório agudo 

**Recomendação - **Utilizar estratégia protetora para tratamento de SDRA em pacientes com lesão neurológica, com monitorização de pressão intra-craniana (PIC) e pressão de perfusão cerebral.^(29,39) ^Mais detalhes no tema específico dessas recomendações.

**Sugestão -** Em casos de SDRA grave, o uso de PEEP alto deve ser individualizado e deve ser monitorizada PIC, porque pode ocorrer elevação dessa quando há diminuição da complacência pulmonar e cerebral concomitantemente.^(^ 40 ^,^ 41 ^)^

Modos ventilatórios

**Sugestão -** Utilizar o modo VCV para pacientes com lesão neurológica grave na fase aguda,^(^ 42 ^)^ visando evitar oscilações de VC.

**Recomendação - **Pacientes com lesão neurológica grave, na fase aguda com hipertensão intracraniana, não devem ser mantidos em modo ventilatório espontâneo.^(^ 43 ^)^

Abordagem de vias aéreas

**Recomendação - **Intubar os pacientes com escala de coma de Glasgow ≤8, com abolição de reflexos de tosse.^(^ 44 ^)^

**Sugestão -** Utilizar intubação sequencial rápida nos pacientes com suspeita ou com hipertensão intracraniana (HIC), minimizando o risco de lesão secundária cerebral pelo aumento da pressão intracraniana. Essa técnica utiliza uma combinação de sedativos com baixo efeito cardiodepressor, como cetamina (1 a 2mg/kg/IV) ou etomidato (0,3mg/kg IV), particularmente nos pacientes hipotensos ou com risco de hipotensão associado a uso de bloqueador neuromuscular de ação rápida (succinilcolina 1,5mg/kg IV) para que, dentro de 45 a 60 segundos, a meta de paralisia e sedação seja atingida.

Estratégias ventilatórias não convencionais

**Sugestão -** Nos pacientes com comprometimento pulmonar grave, individualizar a utilização de novas estratégias ventilatórias como manobras de recrutamento, prona, remoção de CO_2_ por membrana extracorpórea artério-venosa (A-V) e oxigenação por membrana extracorpórea (ECMO) avaliando risco* versus *benefício caso a caso.^(^ 45 ^-^ 47 ^)^

Cabeceira da cama entre 30º e 45º 

**Recomendação - **Manter a cabeceira do leito entre 30º e 45º uma vez que melhora o retorno venoso encefálico e diminui a influência da PEEP sobre a PIC.^(^ 48 ^)^

## Ventilação mecânica nos pacientes com doenças neuromusculares

**Comentário -** Na insuficiência respiratória consequente a doenças neuromusculares, a assistência ventilatória depende da topografia das lesões (desde lesões medulares até o envolvimento muscular direto) (Quadro 1).

**Quadro 1:** Topografia das lesões neuromusculares e exemplos.

Polirradiculoneurite aguda (síndrome de Guillain-Barré)

**Comentário -** Um terço dos pacientes com polirradiculoneurite aguda (síndrome de Guillain-Barré) requer ventilação mecânica durante o curso da doença. Fraqueza generalizada, progressão rápida e envolvimento bulbar estão associados à necessidade de ventilação mecânica nesses pacientes.^(^ 49 ^-^ 52 ^)^

**Sugestão -** Pacientes com polirradiculoneurite aguda devem ser avaliados periodicamente com medidas de pressão inspiratória máxima (PImax) a partir do volume residual, pressão expiratória máxima (PEmax) a partir da CPT e capacidade vital (CV). Pacientes que apresentem PImax <-30cmH_2_O, PEmax <40cmH_2_O, CV<20mL/kg, ou uma redução da CV de mais de 30% devem ser intubados eletivamente para evitar IOT de urgência.^(^ 49 ^-^ 52 ^)^

**Recomendação - **O uso da VNI deve ser feito de forma cuidadosa, devido à instabilidade da polirradiculoneurite aguda. Portanto, a IOT e a ventilação mecânica invasiva não devem ser retardadas, quando houver deterioração da função pulmonar.^(^ 49 ^-^ 52 ^)^

**Sugestão -** A decisão de traqueostomizar pacientes com Guillain-Barré pode ser adiada por 2 semanas. Se, após 2 semanas, as provas de função pulmonar não melhorarem significativamente, traqueostomia deve ser considerada. Se as provas de função pulmonar estiverem melhorando, traqueostomia pode ser adiada, até que o desmame seja realizado.^(^ 49 ^-^ 52 ^)^

Miastenia gravis 

**Comentário -** Insuficiência respiratória aguda ocorre nas crises miastênicas por falência da placa neuromuscular, geralmente acompanhada de fraqueza bulbar. O tratamento específico para crise miastênica com imunoglobulina ou plasmaférese deve ser instituído precocemente.^(^ 53 ^-^ 55 ^)^

**Sugestão -** Considerar o uso da VNI (BiPAP, sigla do inglês *Bilevel Positive Airway Pressure*), em pacientes com crise miastênica, na tentativa de evitar IOT, sendo PCO_2_>50mmHg fator preditivo de falência, podendo ser considerado na fraqueza persistente ou recorrente após extubação.^(^ 53 ^-^ 55 ^)^

**Sugestão -** Avaliar pacientes com crise miastênica periodicamente com medidas de PImax, PEmax e CV. Pacientes que apresentem CV<20mL/kg, PImax<-30cmH_2_O, PEmax>40cmH_2_O podem ser submetidos a uma tentativa de tratamento com VNI (BiPAP) e, caso falhem, devem ser intubados eletivamente, para evitar IOT de urgência.^(^ 53 ^-^ 55 ^)^

**Sugestão -** Realizar um programa respiratório intensivo nos pacientes sob ventilação mecânica por crise miastênica, incluindo suspiros, uso de PEEP, aspiração frequente da árvore brônquica, fisioterapia respiratória, mudança de decúbito e administração de antibioticoterapia, em casos de infecção documentada.^(^ 53 ^-^ 55 ^)^

Distrofia muscular de Duchenne

**Comentário -** A distrofia muscular de Duchenne (DMD) é uma doença de caráter recessivo ligada ao X que ocorre em 1:3.000 nascimentos masculinos. Cursa com perda progressiva da força muscular, com a CV diminuindo progressivamente até a ocorrência de insuficiência respiratória, em geral entre os 18 a 20 anos de idade, com consequente necessidade de suporte ventilatório. A maioria dos pacientes desenvolve cardiomiopatia. A CV forçada (CVF) <1L e a ocorrência de hipoventilação noturna são sinais de mau prognóstico, podendo ser utilizada VNI para melhora dos desfechos de sobrevida e qualidade de vida.^(^ 56 ^-^ 58 ^)^

**Sugestão -** Usar a VNI nos casos de hipoventilação noturna e/ou diminuição de CV (<1L) 

**Sugestão -** A ventilação invasiva, via traqueostomia eletiva é indicada para aqueles pacientes que não toleram a VNI ou que tenham sido intubados por causas agudas.^(^ 56 ^-^ 58 ^)^

Esclerose lateral amiotrófica 

**Comentário -** Esclerose lateral amiotrófica (ELA) é uma doença do neurônio motor, de caráter degenerativo, podendo ocorrer insuficiência respiratória por falência da musculatura no decorrer dela. Aspiração crônica por disfunção da musculatura bulbar e tosse ineficaz são complicações adicionais. A maioria dos pacientes morre de complicações respiratórias com curso variável.^(^ 59 ^,^ 60 ^)^

**Recomendação - **Utilizar VNI em pacientes com ELA, excluindo-se o subgrupo de pacientes com disfunção bulbar grave.

**Recomendação - **Indicar VMI via traqueostomia em pacientes com dificuldade de proteção de vias aéreas e disfunção bulbar grave, após discussão ampla com o paciente e sua família, quanto às complicações e às implicações sociais e logísticas.

**Recomendação - **Critérios para indicação de início do suporte ventilatório levam em consideração a CV: <50% do previsto, PImax <-30cmH_2_O ou <60% do previsto, pico de fluxo expiratório (PFE) <270L/min, PCO_2_>45mmHg e hipoventilação noturna.^(^ 59 ^,^ 60 ^)^

**Sugestão -** Os parâmetros de VNI e invasiva são os seguintes: poderá ser feito com máscara oral ou nasal com os cuidados referentes à adaptação adequada do dispositivo. Deve-se usar BiPAP. Quanto à VMI via traqueostomia geralmente é feita com modo ventilatório de acordo com o tipo de demanda, caso haja doença pulmonar associada. Devem-se monitorizar as ocorrências de atelectasia, o acúmulo de secreção e as pneumonias nesses pacientes.^(^ 61 ^)^

## Ventilação mecânica nos cardiopatas

**Comentário -** O objetivo da ventilação mecânica no paciente cardiopata é adequar a oxigenação e a ventilação, e assegurar o débito cardíaco. 

**Recomendação - **Alcançar SpO_2_ ≥94%, por meio da menor FiO_2_.

**Recomendação - **A ventilação mecânica não invasiva aplicada por pressão positiva contínua nas vias aéreas (CPAP) ou BiPAP é segura, e ambas as modalidades têm efeitos semelhantes e são eficazes em prevenir a IOT. Devem ser aplicadas como forma de suporte ventilatório durante edema agudo pulmonar.^(^ 62 ^-^ 68 ^)^

**Recomendação - **Aplicar a estratégia protetora no paciente cardiopata em ventilação mecânica. ^(^ 69 ^,^ 70 ^)^

**Recomendação - **A aplicação de manobras de recrutamento é segura no paciente cardiopata, com a devida monitorização e os cuidados apropriados.^(69,70)^

**Sugestão -** A monitorização do débito cardíaco e a mensuração da água extravascular pulmonar são sugeridos no paciente cardiopata portador da SDRA em ventilação mecânica, com o objetivo da adequação volêmica e otimização hemodinâmica.^(^ 71 ^)^

**Sugestão -** A monitorização do débito cardíaco, no paciente cardiopata, pode ser realizada por meio do cateter de artéria pulmonar (CAP) ou de maneira não invasiva, por meio do contorno do pulso.^(^ 69 ^,^ 70 ^)^

**Sugestão -** O ecocardiograma transtorácico pode ser realizado no paciente cardiopata em VM com instabilidade hemodinâmica.^(^ 70 ^)^

**Sugestão -** Realizar o ecocardiograma transtorácico nos pacientes submetidos à MR que apresentem instabilidade hemodinâmica para verificar *status* volêmico e disfunção de ventrículo direito (VD).^(^ 69 ^,^ 70 ^)^

**Recomendação - **A retirada da ventilação mecânica no paciente cardiopata segue as recomendações do paciente não cardiopata. O uso de VNI deve ser priorizado para facilitar o processo de retirada de ventilação mecânica, devendo ser aplicada imediatamente após à extubação.^(^ 62 ^-^ 70 ^)^

**Sugestão -** Níveis elevados de peptídeo natriurético cerebral (BNP) durante o desmame ventilatório no cardiopata têm acurácia para predizer falha de desmame.^(^ 70 ^,^ 72 ^)^

**Recomendação - **Balanço hídrico positivo deve ser evitado no paciente cardiopata em VM sem instabilidade hemodinâmica.^(^ 70 ^)^

**Recomendação - **Óxido nítrico inalatório é estratégia eficaz no paciente cardiopata com disfunção de VD e hipertensão pulmonar em VM.^(^ 70 ^)^

**Recomendação - **Não se recomenda a opção por uma modalidade ventilatória em detrimento da outra no paciente cardiopata.^(^ 70 ^)^

**Sugestão -** Nos pacientes sob uso de inotrópico, pode-se manter o uso até após a extubação.^(^ 70 ^)^

Ventilação mecânica no paciente cardiopata submetido à cirurgia

Volume corrente

**Recomendação - **A utilização de VC de 6mL/kg de peso predito, na modalidade volume controlado ou pico/platô de pressão inspiratória, é suficiente para manter esse mesmo volume na PCV.^(^ 70 ^)^

Pressão positiva ao final da expiração 

**Recomendação - **Aplicar PEEP durante anestesia geral, por associar-se à melhora da oxigenação e à prevenção da formação de atelectasias.^(70)^

Manobras de recrutamento alveolar

**Sugestão -** Podem-se utilizar as manobras de recrutamento no intraoperatório com o objetivo de evitar o colapso alveolar.^(^ 69 ^)^

Fração inspirada de oxigênio

**Recomendação - **Na indução anestésica, utilizar FiO_2_ de 100%, para assegurar oxigenação adequada para a realização da intubação. Recomendam-se frações de oxigênio necessárias para manutenção da SpO_2_ >94%.^(^ 70 ^)^

Retirada da ventilação mecânica

**Recomendação - **A retirada da VM deve ser gradual, podendo ser realizada a pressão de suporte (PSV). A VNI é recurso importante, que deve ser usado imediatamente após a extubação.^(^ 62 ^-^ 70 ^)^

Analgesia pós-operatória

**Recomendação - **A obtenção de analgesia pós-operatória adequada associa-se à otimização da função pulmonar pós-operatória.

## Ventilação mecânica nas doenças intersticiais pulmonares

**Comentário -** As doenças pulmonares intersticiais (DPI) são um grupo heterogêneo de doenças que acomete predominantemente o interstício pulmonar, com variados graus de inflamação e fibrose,^(73,74) ^podendo evoluir com graus variáveis de hipoxemia e diminuição progressiva dos volumes pulmonares. Pacientes com DPI podem requerer ventilação mecânica por uma série de fatores, entre eles durante a anestesia em procedimentos cirúrgicos, como biópsia pulmonar a céu aberto ou outras cirurgias eletivas ou de emergência, por infecções respiratórias que levem à insuficiência respiratória, ou por exacerbações agudas (EA - não infecciosas) da doença intersticial de base.^(^ 75 ^,^ 76 ^)^

Indicação de ventilação mecânica

**Comentário -** A insuficiência respiratória em pacientes com DPI deve ser dividida em dois grupos: evolução da doença de base e EA. As EA são caracterizadas por piora aguda, em geral nos últimos 30 dias, sem causa aparente, dos sintomas clínicos da DPI, em especial dispneia e tosse, acompanhadas de piora radiológica, frequentemente na forma de vidro fosco sobreposto às alterações prévias. As EA foram inicialmente descritas em pacientes com fibrose pulmonar idiopática (FPI), mas podem ocorrer em outras formas de DPI.^(76-79) ^A incidência de EA parece ocorrer em algum momento no curso da FPI em até 5 a 10% dos pacientes, com mortalidade daqueles que necessitam de VM próxima de 100% 

**Recomendação - **Antes de se caracterizar uma EA, devem ser afastadas infecções, tromboembolismo pulmonar, disfunções cardíacas e toxicidade pulmonar por drogas, entre outras.^(^ 76 ^-^ 80 ^)^

Complicação aguda

**Sugestão -** Nas EA de DPI, deve ser avaliada a condição prévia do paciente. Indicar ventilação mecânica invasiva quando não se caracterizar que a causa da insuficiência respiratória aguda seja por evolução da doença de base. 

Evolução da doença de base

**Recomendação - **Deve ser evitada a indicação de internação em UTI e de ventilação mecânica invasiva, devendo ser discutido com o paciente ou com os familiares.

Ventilação mecânica não invasiva

**Sugestão -** A VNI pode ser usada como tratamento inicial de pacientes com DPI que desenvolvam insuficiência respiratória aguda, ou como suporte ventilatório paliativo para pacientes que previamente expressaram o desejo de não serem intubados. Pode ser usado CPAP ou VNI com dois níveis de pressão, aplicados precocemente.

**Recomendação - **O uso de VNI deve ser monitorado por profissional da saúde à beira do leito de 0,5 a 2 horas. Para ser considerado com sucesso, deve ser observada a diminuição da f, o aumento do VC, a melhora do nível de consciência, a diminuição ou a cessação de uso de musculatura acessória, o aumento da PaO_2_ e/ou da SpO_2_ e a diminuição da PaCO_2_ sem distensão abdominal significativa. Quando não há sucesso, recomenda-se imediata IOT e ventilação invasiva. Espera-se sucesso nessa população em 50%.^(^ 80 ^)^

Ventilação mecânica invasiva

**Comentário -** Como o achado histológico nas EA é de dano alveolar difuso (DAD), semelhante ao observado em pacientes com SDRA, e na falta de estudos prospectivos, alguns autores sugerem que estratégias utilizadas para a SDRA poderiam ser extrapoladas para pacientes com EA de DPI. Assim, alguns especialistas advogam o uso de ventilação protetora, com volumes correntes baixos, em termo de 6mL/kg de peso ideal, e limitação da PPlatô ≤30cmH_2_O.^(80-84) ^O uso de PEEP elevada não foi testado em nenhum estudo para pacientes com DPI. Dois estudos retrospectivos encontraram associação da PEEP com o desfecho de pacientes com DPI submetidos à VM: Suh et al. descreveram baixa mortalidade em um grupo de pacientes com pneumonia intersticial aguda que receberam uma estratégia de intervenção precoce, que envolvia uma série de medidas, entre elas VM com volumes correntes baixos e PEEP moderada, com mediana de 11cmH_2_O.^(^ 79 ^)^ Por outro lado, em um grupo mais heterogêneo de pacientes com DPI submetidos a VM, Fernandez-Perez et al. observaram que PEEP >10cmH_2_O no primeiro dia de VM esteve associada à maior mortalidade, porém os próprios autores comentam que a PEEP alta pode ser marcadora de maior gravidade da insuficiência respiratória.^(^ 76 ^)^

**Sugestão -** Pacientes com DPI, que necessitam de VM, devem ser ventilados com baixos volumes correntes, em torno de 6mL/Kg de peso ideal, e limitação da PPlatô <30cmH_2_O; podem ser usadas altas frequências respiratórias, >30 rpm, e tempo inspiratório curto, para evitar hipercapnia. Usar PEEP entre 5 e 10cmH_2_O.

**Sugestão -** A utilização de PEEP elevada pode ser tentada com cautela e deve ser individualizada para cada paciente. Manobras de resgate para hipoxemia refratária, como posição prona, MR, óxido nítrico podem ser usados em centros de referência com experiência em tais manobras. 

## Retirada do paciente da ventilação mecânica

Identificar o paciente apto para iniciar desmame

**Recomendação - **Retirar o paciente da ventilação invasiva o mais rápido quanto clinicamente possível.^(^ 85 ^,^ 86 ^)^

**Recomendação - **Nas diretrizes internas das UTI, deixar claro as definições envolvendo a retirada da ventilação mecânica. O conceito de "sucesso de desmame" refere-se ao paciente que tem sucesso no teste de respiração espontânea (TRE), ainda conectado ao ventilador. Já "sucesso de extubação" refere-se ao paciente que tem a prótese endolaríngea retirada (extubação) após passar no TRE e não é reintubado nas próximas 48 horas. No caso dos traqueostomizados, equivalerá ao sucesso na extubação o paciente que tolerou desconexão do ventilador após passar no TRE e não precisou voltar a ser reconectado ao ventilador nas próximas 48 horas.^(^ 87 ^-^ 89 ^)^

**Recomendação - **Avaliar e identificar diariamente o paciente (busca ativa por meio de diretrizes internas preestabelecidas pela equipe multiprofissional) com vistas à possibilidade de descontinuar a ventilação, visando diminuir o tempo de ventilação mecânica e haver um menor custo.^(^ 90 ^-^ 94 ^)^

Sedação 

**Recomendação - **Deve-se realizar a suspensão diária da sedação, para se verificar a capacidade de ventilação espontânea do paciente.^(^ 95 ^)^ (vide tema Sedação e Analgesia, destas Recomendações - Parte 1).

Critérios para considerar a aptidão para o desmame

**Recomendação - **Realizar a busca ativa, incluindo os tópicos do quadro 2.^(^ 88 ^,^ 90 ^-^ 96 ^)^

**Quadro 2:** Tópicos a serem buscados de forma ativa rotineiramente nos pacientes sob ventilação mecânica. PaO2 - pressão parcial de oxigênio; FIO2 - fração inspirada de oxigênio; PEEP - pressão expiratória final positiva.

### Índices preditivos

**Recomendação - **Os índices preditivos de desmame com melhor acurácia são a taxa da f dividida pelo VC (f/VC) ou índice de ventilação superficial rápida (IVSR), e o índice de desmame integrado (*Integrative Weaning Index* - IWI) (Quadro 3). Eles só devem ser calculados em situações de difícil decisão e NÃO como um instrumento isolado na tomada de decisão para se realizar o TRE.^(^ 97 ^-^ 99 ^)^

**Quadro 3:** Parâmetros com significância para predizer o sucesso do desmame. CROP - índice de complacência, frequência respiratória, oxigenação e pressão; PImax - pressão inspiratória máxima; f - frequência respiratória; VC - volume corrente.

### Teste de autonomia respiratória (teste de respiração espontânea)

**Recomendação - **No TRE, o paciente deve ser colocado em tubo em T ou PSV de 5 a 7cmH_2_O durante 30 a 120 minutos. Durante o TRE, o paciente deve ser monitorizado para sinais de insucesso.^(85-94) ^São considerados com sucesso no TRE os pacientes que mantiverem padrão respiratório, troca gasosa, estabilidade hemodinâmica e conforto adequados (Quadro 4). ^(^ 85 ^-^ 94 ^,^ 100 ^-^ 102 ^)^

**Quadro 4:** Sinais de intolerância ao teste de respiração espontânea.

### **Recomendação - **Após um TRE bem-sucedido, avaliar se as vias aéreas estão pérvias e se o paciente é capaz de protegê-las.

### Como avaliar o momento da extubação

Avaliação da proteção das vias aéreas

**Recomendação - **Avaliar se o paciente tem nível consciência (escala de coma de Glasgow >8), tosse eficaz (teste do cartão branco positivo e pico de fluxo >60 lpm) e pouca secreção (sem necessidade de aspiração a cada 1 ou 2 horas).^(^ 103 ^)^

Avaliação da permeabilidade das vias aéreas

**Sugestão -** Testar a permeabilidade das vias aéreas em pacientes de maior risco para estridor laríngeo e obstrução das vias aéreas (ventilação prolongada e trauma), podendo ser feito pelo método qualitativo ou quantitativo. Aspirar bem boca e laringe antes da desinsuflação do balão da prótese para o teste, a fim de evitar entrada de material indesejado nas vias aéreas inferiores de forma iatrogênica (Quadro 5).^(^ 104 ^)^

**Quadro 5:** Como realizar o teste de vazamento do balonete do tubo traqueal (cuff-leak test) em pacientes ventilados mecanicamente. VCV - ventilação cliclada a volume; VCe - volume corrente expirado; VCi - volume corrente inspirado.

### Uso de corticoides

**Recomendação - **Em pacientes de alto risco para estridor laríngeo e edema laríngeo, avaliados pelo teste de permeabilidade (*cuff leak test*), pode haver benefício com o uso preventivo de corticoide. As doses descritas oscilam entre 20 e 40mg de metilprednisolona IV a cada 4 a 6 horas, iniciadas pelo menos 4 horas, mais comumente 12 a 24 horas antes da extubação.^(^ 105 ^)^

### Uso da ventilação não invasiva na retirada da ventilação mecânica 

Uso da ventilação não invasiva para facilitar a retirada da ventilação mecânica - desmame precoce (ventilação não invasiva facilitadora)

**Recomendação - **Recomenda-se o uso da VNI como facilitadora de retirada da VM de forma precoce em pacientes portadores de doença pulmonar obstrutiva crônica (DPOC), mesmo naqueles que não passaram no TRE, desde que sob adequada condição clínica. O paciente deve ser conduzido em centros com experiência no uso de VNI (Figura 1).^(^ 106 ^)^

### Uso da ventilação não invasiva para prevenir a falha de extubação (ventilação não invasiva preventiva)

**Recomendação - **Deve-se fazer uso de VNI imediatamente após a extubação, de forma preventiva, em pacientes selecionados como de maior risco, especialmente nos hipercápnicos (Figura 1 e Quadro 6).^(^ 107 ^-^ 111 ^)^

**Quadro 6:** Ventilação não invasiva preventiva - fatores de risco para falência respiratória. VMI - ventilação mecânica invasiva.

**Figura 1:** Utilização da ventilação não invasiva para retirada da ventilação mecânica. TRE - teste de respiração espontânea; VNI - ventilação não invasiva; DPOC - doença pulmonar obstrutiva crônica; IResp - insuficiência respiratória aguda ou agudizada.

Uso da ventilação não invasiva na falência respiratória após a extubação (ventilação não invasiva curativa) 

**Recomendação - **Evitar o uso da VNI após nova falência respiratória se apresentar em pacientes extubados até 48 horas. Não retardar a reintubação nessa situação, exceto em grupos cirúrgicos que desenvolvam falência respiratória no pós-operatório (Figura 1).^(^ 112 ^)^

Como conduzir o paciente com falência de desmame (não obteve êxito no primeiro teste de respiração espontânea)

**Recomendação - **Reconduzir para um suporte ventilatório que lhe proporcione conforto e trocas gasosas adequadas, por um período de 24 horas, para poder repetir o TRE. Procurar identificar as causas da falha^(86) ^(para pacientes com desmame difícil ou prolongado, vide tema específico destas recomendações).

Técnicas de desmame gradual

**Recomendação - **Evitar o uso do modo *Synchronized Inspiratory Mandatory Ventilation* (SIMV), pois pode prolongar o período de retirada da VMI.^(^ 113 ^)^

Como conduzir o paciente com falência de extubação 

**Recomendação - **Reintubar o paciente o quanto antes; identificar e tratar as causas da falência; e, assim que possível, reiniciar o processo de retirada (exceção: pode-se tentar VNI curativa no paciente cirúrgico). 

## Paciente com desmame prolongado

**Sugestão -** Usar definições de classificação da duração do desmame para enquadrar seu paciente, em desmame simples^(^ 114 ^)^ (quando houver sucesso no primeiro TRE), desmame difícil (quando o paciente falhar no primeiro TRE e necessitar de até três TRE ou até sete dias após o primeiro TRE) e, por fim, desmame prolongado (quando o paciente falhar em mais de três TRE consecutivos ou com necessidade >7 dias após o primeiro TRE).

**Sugestão -** Usar o conceito de ventilação mecânica prolongada (VMP) como necessidade de VM ≥21 dias consecutivos por mais de 6 horas por dia.

**Recomendação - **Identificar causas de falha na retirada da VM (Quadro 7).^(^ 88 ^,^ 114 ^-^ 130 ^)^

**Quadro 7:** Causas de falha na retirada da ventilação mecânica.(89,115-131)

Distúrbios musculares 

**Sugestão -** Avaliar possibilidade de polineuropatia da doença crítica e distúrbios de fósforo, magnésio, cálcio e potássio. 

Doenças endócrinas e metabólicas

**Recomendação - **Realizar o controle adequado da diabetes, do hipotireoidismo e da insuficiência suprarrenal. 

**Comentário -** A obesidade pode ser um fator a mais para o desmame prolongado, pois cursa com aumento do consumo de O_2_ (VO_2_) e da produção de CO_2 _(VCO_2_), diminuição da complacência estática, da CV, da CPT e de possível aumento da PIA e da Rva.

Distúrbios hidroeletrolíticos e ácido-base

**Recomendação - **Realizar monitorização, diagnóstico e tratamento dos estados de hiper-hidratação, que se associam a aumento de morbidade, mortalidade e tempo de permanência na UTI. Identificar e resolver casos de alcalose metabólica que têm por causas mais comuns a acidose respiratória crônica e o uso de diuréticos. A alcalose metabólica está associada com: aumento de mortalidade, diminuição do estímulo respiratório, da oferta de O_2_ (DO_2_), desvio da saturação da oxihemoglobina para a esquerda, distúrbio ventilação/perfusão (V/Q) e vasoconstricção sistêmica. Realizar o adequado processo de nutrição (vide tema específico nestas recomendações), visando evitar a desnutrição, o aumento do catabolismo proteico, a diminuição da massa muscular corporal e da bomba torácica, com diminuição da força e resistência e aumento da VO_2_, e perpetuando a dependência ao ventilador. Diminuição da albumina está associada com desmame prolongado e deve ser monitorizada.^(^ 114 ^-^ 130 ^)^

Estratégias para reabilitar e facilitar a retirada da ventilação mecânica 

Reavaliação da doença de base e comorbidades

**Recomendação - **Tratar o máximo possível as doenças de base cardíacas, pulmonares, psiquiátricas e infecciosas, e manter nutrição adequada para o quadro clínico.

Cuidados específicos para retirada da ventilação mecânica 

**Sugestão -** Se disponível, no desmame prolongado, transferir o paciente para uma unidade especializada em retirada da ventilação mecânica. 

**Recomendação - **Indicar traqueostomia em pacientes que falharam repetidamente em TRE, a partir do 10^o^ dia de VM, como parte de um protocolo de retirada e de acordo com especificações no tema relativo a esse tópico nestas Recomendações.

**Sugestão -** Realizar TRE com uso de colar ou peça T diariamente. Na tolerância (f<35/minuto ou acima de 35/minuto por menos de 5 minutos consecutivos; SaO2 >90%; FC<140/minuto ou alteração sustentada em qualquer sentido de 20%; 90mmHg>PA<180mmHg com ausência de ansiedade e diaforese), aumentar progressivamente o tempo de uso da peça T, realizar repouso noturno em ventilação assisto-controlada e, nos casos de falha, retornar para modos assistido-controlados para repouso e visando à nova tentativa em 24 horas.^(^ 123 ^)^

**Sugestão -** Caracteriza-se como ventilação mecânica de longo prazo quando houver falha de todo processo de retirada, especialmente em pacientes com trauma raquimedular (TRM), DPOC em estágio terminal, demência avançada, fibrose pulmonar e doença neuromuscular em evolução irreversível. Nessas situações, conceituar tratamento fútil e paliativo para o paciente e familiar, concluindo em conjunto a melhor conduta.^(^ 114 ^)^

**Recomendação - **A fisioterapia precoce e a mobilização passiva devem ser realizadas nos pacientes em ventilação mecânica e também durante o processo de retirada. Essas atividades são consideradas seguras e estão associadas a melhores resultados funcionais e menor tempo de ventilação mecânica^(121,130) ^(vide tema específico nestas recomendações). 

**Sugestão -** O treinamento muscular inspiratório pode ser considerado em pacientes que falharam no desmame, com o objetivo de elevar a PImax e facilitar a retirada do suporte ventilatório.^(^ 130 ^,^ 131 ^)^

## Alterações e cuidados hemodinâmicos no paciente sob ventilação mecânica invasiva

### **Comentário -** Os efeitos cardiovasculares da ventilação mecânica com pressão positiva estão descritos no quadro 8.

**Quadro 8:** Efeitos cardiovasculares da ventilação mecânica com pressão positiva VD - ventrículo direito; VE - venctriculo esquerdo.

Cuidados hemodinâmicos nos pacientes sob ventilação mecânica

**Recomendação - **A ressuscitação da perfusão tecidual se inicia com a fase aguda precoce, na qual se deve realizar a ressuscitação o mais rápido possível objetivando saturação venosa mista de O_2_ (SvO_2_) >65%, ou saturação venosa central (SvcO_2_) >70%, lactato arterial <2mmol/L (18mg/dL) e adequação da DO_2_/VO_2_.^(132-135) ^O processo deve seguir na fase pós-ressuscitação (quando se atingir perfusão tecidual adequada), sendo que se deve realizar restrição hídrica para manter balanço hídrico menor ou igual a zero.^(^ 136 ^)^

**Sugestão -** Em paciente com SDRA utilizando PEEP ≥15cmH_2_O e <20cmH_2_O, fazer ecocardiograma pelo menos uma vez e, se necessário, monitorar débito cardíaco.^(137,138) ^Em paciente com SDRA e PEEP ≥20cmH_2_O ou instabilidade hemodinâmica, monitorar com ecocardiograma seriado e/ou com CAP volumétrico, se disponível.^(^ 137 ^,^ 138 ^)^

**Sugestão -** Durante a MRM com titulação da PEEP pelo método decremental, monitorizar paciente com pressão arterial invasiva, realizar ecocardiograma após 6 horas para avaliar função VD, ou precocemente se apresentar instabilidade hemodinâmica.^(^ 137 ^,^ 138 ^)^

**Sugestão -** Na SDRA moderada/grave, considerar monitorização da água extravascular pulmonar (se disponível).^(139,140)^

**Sugestão -** Evitar vasodilatadores sistêmicos em hipoxemia refratária (inibem a vasoconstrição hipóxica).^(^ 141 ^)^

**Sugestão -** Interpretar a SvO_2_, considerando a PaO_2_. Valores elevados de PaO_2_ podem aumentar a SvO_2_.^(^ 142 ^)^

Ventilação mecânica no paciente com falência de ventrículo esquerdo 

**Recomendação - **Para o diagnóstico da falência de ventrículo esquerdo (VE) utilizar o ecodoplercardiograma que deve demonstrar fração de ejeção (FE), velocidade-tempo integral (VTI), bem como avaliar a função diastólica [E/A, E/E', volume diastólico final global].^(^ 143 ^)^ Se utilizar o CAP, os valores da pressão de oclusão da artéria pulmonar (POAP) >18mmHg e de índice cardíaco (IC) <2,2L/min/m^2^ caracterizam a falência de VE.^(^ 143 ^)^

**Recomendação - **Utilizar inotrópicos, vasopressores se necessário, diuréticos e vasodilatadores quando possível. Em casos selecionados, realizar suporte circulatório mecânico.^(^ 143 ^)^

**Sugestão -** Favorecer PEEP elevada (por diminuição da pré-carga e da pós-carga do VE). Se houver falência de VD concomitante, aumentar PEEP com cuidado (monitorizar o VD e fluxo). ^(^ 144 ^)^ Evitar hipercapnia grave (pH<7,15 ou PaCO_2_ >80mmHg).^(^ 145 ^)^ Considerar ultrafiltração renal visando ao balanço hídrico negativo nas situações de refratariedade ao uso de diuréticos.^(^ 146 ^)^

Ventilação mecânica no paciente com falência de ventrículo direito 

**Recomendação - **Quanto ao diagnóstico, usar ecodoplercardiograma (diâmetro diastólico do VD >3,5cm, relação VD/VE >1, retificação ou movimento paradoxal do septo interventricular, pressão sistólica da artéria pulmonar (PSAP) >35mmHg, *tricuspid annular plane systolic excursion *(TAPSE) <1,8cm)^(^ 147 ^)^ ou o CAP (se disponível tipo volumétrico): pressão venosa central (PVC) > POAP, pressão média da artéria pulmonar (PMAP) >25mmHg, índice sistólico (IS) <30mL.min^-1^.m^-2^, índice de volume diastólico final de ventrículo direito (IVDFVD) >140mL.m^-2^.^(^ 137 ^)^

**Sugestão -** Detalhes sobre a monitorização, tratamento e cuidados específicos encontram-se no quadro 9 e na figura 2.

**Quadro 9:** Sugestões em monitorização, tratamento e cuidados espefícos no paciente com falência de ventrículo direito.(146,148-152) CAP - cateter de artéria pulmonar; PEEP - pressão expiratória final positiva; VC - volume corrente; VD - ventrículo direito.

**Figura 2:** Algoritmo de condução hemodinâmica no paciente sob ventilação mecânica. VE - ventrículo esquerdo; VD - ventrículo direito; VM - ventilação mecânica; PEEP - pressão expiratória final positiva.

### Recursos disponíveis de monitorização hemodinâmica no paciente sob ventilação mecânica

**Sugestão -** Métodos de monitorização hemodinâmica em pacientes sob VM sugeridos incluem a previsão de resposta a volume (aumento >15% do índice cardíaco em pacientes com com as seguintes características: PEEP <10 cmH_2_O, VC 8 a 10mL.kg-1 peso ideal, f <30 min-1, complacência respiratória >30mL.cmH_2_O, ausência de arritmias, ausência de esforço respiratório, ausência de cor pulmonale, considerar delta-PP (pressão de pulso) >13%, variação de volume sistólico (VVS) >10%, delta-VTI (*velocity time integral*) >15%, colapsabilidade de cava superior >36% e distensibilidade da veia cava inferior >18%. ^(153,154) ^Realizar também a manobra com oclusão de válvula expiratória - em pacientes com ventilação espontânea e PEEP ≤10cmH_2_O,^(^ 155 ^)^ delta-PVC (delta de pressão venosa central) >1mmHg (1,36cmH_2_O). ^(^ 156 ^)^ Nos pacientes com PEEP alta e/ou VC baixo, podem-se usar manobra de elevação de membros inferiores,^(^ 157 ^)^ prova volêmica com pequenas alíquotas de fluido (250mL) e monitoração do índice cardíaco (Figura 2).^(^ 158 ^)^

## Cuidados de fonoaudiologia na reabilitação do paciente pós-ventilação mecânica

**Comentário -** A atuação do fonoaudiólogo em UTI está respaldada pelo RDC 07/2010 da Agência Nacional de Vigilância Sanitária (ANVISA). Em disfagia, a atuação do fonoaudiólogo em UTI foi regulamentada pelo Conselho Federal de Fonoaudiologia, pela Resolução 356, de 6 de dezembro de 2008, e publicada no Diário Oficial da União, em 9 de dezembro de 2008. Na equipe multiprofissional, o fonoaudiólogo avalia a segurança da deglutição por via oral e realiza o gerenciamento da deglutição, podendo contribuir para a prevenção de pneumonias aspirativas e com o processo de desmame da traqueostomia.

**Sugestão -** Solicitar avaliação fonoaudiológica^(^ 159 ^-^ 165 ^)^ para todos os pacientes que passaram por IOT por período ≥48 horas, para os pacientes que foram submetidos à IOT de repetição (recorrente) e para os que estão traqueostomizados (com ou sem ventilação mecânica).

**Recomendação - **Com relação ao momento da avaliação fonoaudiológica após a extubacão, recomendam-se avaliar os pacientes após 24^(^ 166 ^)^ a 48 horas e instituir fonoterapia nos pacientes disfágicos com risco de aspiração.^(^ 167 ^-^ 171 ^)^

**Sugestão -** NÃO realizar terapia fonoaudiológica em pacientes durante a IOT, por falta de evidências clínicas quanto aos benefícios na função de deglutição, embora seja indicada a identificação precoce dos pacientes que, mesmo em IOT, apresentem vários fatores de risco associados, que podem comprometer a dinâmica da deglutição (neuropatas, especialmente doença de Parkinson e pós-AVC e demenciados). 

**Sugestão -** Realizar a avaliação fonoaudiológica clínica à beira do leito (estrutural e funcional) ^(163,172) ^e indicar a necessidade de exames objetivos da deglutição (nasofibroscopia funcional e videodeglutograma).^(^ 173 ^)^

**Sugestão -** A fonoaudióloga deve definir o tipo de consistência alimentar e a necessidade do uso de espessantes para a administração de líquidos, em concordância com o serviço de nutrição, quando liberada a dieta via oral.

**Sugestão -** Adaptar válvula de fala (no circuito da ventilação mecânica ou diretamente na traqueostomia), com o auxílio do fisioterapeuta e/ou médico, desde que sejam avaliadas a viabilidade e a tolerância à desinsuflação do balonete (*cuff*).^(^ 174 ^,^ 175 ^)^

**Sugestão -** No caso de pacientes traqueostomizados, modificar a cor dos alimentos na oferta da dieta por via oral e/ou na avaliação da deglutição de saliva, usando-se corante alimentício azul, para verificar a ocorrência de saída de saliva e/ou secreções de cor azul pela traqueostomia, caracterizando o evento aspiração.^(^ 176 ^-^ 178 ^)^

**Sugestão -** Verificar sinais e sintomas de disfagia durante a oferta da dieta por via oral, especialmente aqueles que podem estar associados à broncoaspiração, como engasgos, tosse, voz molhada.^(^ 179 ^-^ 181 ^)^

**Sugestão -** Discutir com a equipe médica a utilização de medicamento xerostômico, nos casos de pacientes em ventilação mecânica e/ou traqueostomizados e que não tolerem a desinsuflação do *cuff* ou que apresentem grande quantidade de aspiração de saliva.^(^ 182 ^)^

**Sugestão -** Realizar terapia indireta e direta da deglutição nos pacientes disfágicos e/ou com risco de aspiração.^(^ 183 ^)^

## Cuidados de enfermagem nos pacientes em suporte ventilatório invasivo e não invasivo

**Comentário -** A enfermagem como integrante da equipe multidisciplinar da UTI, participa ativamente das ações administrativas e assistenciais que envolvem o suporte invasivo e não invasivo nos pacientes em ventilação mecânica.

Cuidados com circuito, filtros e umidificadores

**Recomendação - **Manter umidificação e aquecimentos das vias aéreas inferiores durante a ventilação mecânica.

**Recomendação - **Trocar os dispositivos trocadores de umidade e calor de a cada 7 dias (higroscópico e hidrofóbico), desde que sejam mantidas a altura e a posição adequadas do dispositivo em relação ao tubo endotraqueal (o dispositivo deve ficar vertical, conectado ao tubo e ao circuito, de forma que as microgotas e sujidades não o inundem). Em caso de sujidade, condensação ou dano, o filtro deve ser trocado.^(^ 184 ^)^

**Recomendação - **Não realizar troca rotineira do circuito do ventilador mecânico, somente quando apresentar sujidade visível a olho nu, dano ou ventilação prolongada (>30 dias).^(^ 185 ^,^ 186 ^)^

Limpeza e conservação dos equipamentos

**Recomendação - **Os circuitos dos ventiladores mecânicos requerem desinfecção de alto nível (hipoclorito de sódio na concentração de 0,5% e tempo de contato de 60 minutos), ou esterilização.^(^ 187 ^)^

Cuidados durante banho de leito e mudança de decúbito

**Recomendação - **Realizar avaliação dos sinais vitais, análise e registro dos parâmetros do ventilador mecânico (modo ventilatório, pressão de pico, PEEP, f, VC e FIO_2_), checagem dos alarmes e de parâmetros clínicos antes da realização do banho de leito e da mudança de decúbito. Manter a monitorização cardíaca e da SatO_2 _durante banho de leito e mudança de decúbito. Observar um período de equilíbrio de 5 a 10 minutos antes de determinar a intolerância/instabilidade hemodinâmica pela mudança de decúbito e/ou banho de leito.^(^ 188 ^,^ 189 ^)^

**Recomendação - **Discutir com a equipe multiprofissional o momento mais adequado para o banho de leito em pacientes graves clinicamente instáveis. O enfermeiro deve avaliar o paciente antes de liberar a realização do banho, adiando-o no caso de gravidade que possa comprometer a segurança do paciente.

**Recomendação - **Realizar mudança de decúbito a cada 2 horas, com lençol móvel e, no mínimo, com dois profissionais de enfermagem.^(^ 190 ^)^

**Sugestão -** Realizar terapia de rotação lateral contínua (CLRT, sigla do inglês *continuous lateral rotation therapy*) com utilização da cama cinética (*kinetic bed therapy*), quando disponível.^(^ 191 ^)^

**Recomendação - **Manter elevada a cabeceira da cama dos pacientes ventilados para uma posição entre 30º a 45º. As evidências são conflitantes a respeito de aspiração de conteúdo gástrico (45º) e úlceras por pressão (30º). Há preferência pela posição de 30°, enquanto esta não representar riscos ou conflitos com procedimentos médicos e de enfermagem.^(^ 192 ^)^

**Sugestão -** Utilizar a posição tipo "cadeira de praia" (*beach chair position* - BCP) de 2 a 4 vezes/dia, que exige menos pessoal do que outras intervenções, permitindo mobilidade precoce de pacientes de UTI e melhora da função pulmonar.^(^ 193 ^)^

**Recomendação - **Manter a pressão do balonete da prótese traqueal entre 18 a 22mmHg ou 25 a 30cmH_2_O (cuffômetro) visando evitar vazamentos de ar sem compressão excessiva da mucosa traqueal. Evitar pressões do balonete >22mmHg ou 30cmH_2_O. Verificar a pressão balonete no mínimo quatro vezes ao dia e antes de realizar a higiene bucal.

**Recomendação - **Manter o tubo traqueal fixado e centralizado com fixador adesivo ou cadarço, para que ocorra uma distribuição homogênea da pressão do balonete na traqueia. Atentar para as lesões na cavidade oral, comissura labial e face.^(^ 194 ^)^

**Recomendação - **Cuidados durante a mudança de decúbito e ao lateralizar o paciente no banho de leito estão discriminados no quadro 10.^(^ 195 ^)^

**Quadro 10:** Cuidados durante a mudança de decúbito e ao lateralizar o paciente no banho de leito.

**Recomendação - **Em pacientes em posição prona, recomenda-se que o procedimento seja realizado com pelo menos cinco membros da equipe da UTI presentes, incluindo-se pelo menos um médico e um enfermeiro. Devem-se utilizar proteções para a pele das regiões: frontal, nariz, joelhos, crista ilíaca, genitália e mamilos. A rotação do paciente deve ser em dois passos com total atenção aos dispositivos invasivos. Deve-se monitorizar o eletrocardiograma no dorso e a posição Trendelemburg reverso pode ser utilizada para diminuir o edema facial.^(^ 194 ^,^ 195 ^)^

**Recomendação - **Utilizar o sistema de aspiração fechado para aspiração traqueal em pacientes instáveis hemodinamicamente, para evitar dessaturação em pacientes de risco (por exemplo: neuropatas), em pacientes com SDRA com PEEP ≥10cmH_2_O para manter RM e para prevenir atelectasias. Deve-se trocar o sistema a cada 7 dias. O sistema de aspiração fechado não mostrou diminuição no desenvolvimento de pneumonia associada à VM (PAV), na mortalidade e no tempo de internação em UTI quando comparado ao sistema aberto.^(^ 196 ^)^

Cuidados específicos na higiene bucal, alimentação oral e enteral

**Recomendação - **É preconizada a realização de higiene bucal com escovação a cada 12 horas, com solução aquosa de digluconato de clorexidina a 0,12%. Nos intervalos, a higiene bucal deve ser realizada com água destilada ou filtrada e/ou aromatizante bucal sem álcool quatro vezes/dia.^(^ 196 ^-^ 198 ^)^

**Recomendação - **Verificar a pressão do balonete do tubo orotraqueal ou traqueostomia antes de realizar a higiene bucal.^(^ 189 ^)^

**Recomendação - **As vias gástrica e pós-pilórica podem ser utilizadas para alimentação enteral em pacientes sob VM, reservando o posicionamento pós-pilórico para pacientes com intolerância e/ou contraindicação gástrica.^(^ 200 ^)^

**Recomendação - **Utilizar dispositivo fixador de sonda nasoenteral (dispositivo disponível mercado ou adequado adesivo) para redução da taxa de deslocamento da sonda não intencional.^(^ 201 ^)^

**Sugestão -** Monitorar a diferença entre a nutrição entérica prescrita e a recebida como um marcador de aceitação da dieta.^(^ 202 ^)^

## Cuidados de fisioterapia nos pacientes em suporte ventilatório

**Comentários -** Pacientes internados na UTI podem apresentar disfunções respiratórias e musculares, e, ao longo do tempo, desenvolver fraqueza neuromuscular e complicações do imobilismo, o que pode dificultar a retirada da ventilação mecânica. A imobilidade prolongada leva à perda das funções motoras e da qualidade de vida, podendo ser minimizadas com a instituição de mobilização precoce e cuidados respiratórios. Pacientes que requerem VMP apresentam incidência de fraqueza muscular adquirida na UTI (neuromuscular) entre 25 e 60%,^(^ 203 ^)^ que contribui para o aumento do tempo de internação na UTI e hospitalar. A fisioterapia atua no sentido de manter e/ou restabelecer a funcionalidade do paciente por meio da prevenção de alterações osteomioarticulares e de complicações respiratórias.

**Recomendação - **Diagnóstico fisioterapêutico deve preceder qualquer intervenção.^(^ 204 ^)^

**Recomendação - **A fisioterapia em pacientes sob ventilação mecânica na UTI deve ser implementada em regime de 24 horas, com benefícios na redução do tempo de ventilação mecânica, tempo de internação em UTI e hospitalar, na redução do custo hospitalar e da mortalidade. ^(^ 200 ^,^ 205 ^)^

Manobras e condutas fisioterapêuticas no paciente em ventilação mecânica

**Recomendação - **Terapia de higiene brônquica (posicionamento, insuflação manual, vibração e compressão torácica): indicada em pacientes com aumento de Rva gerada por presença de secreção, causando assincronia da ventilação mecânica e/ou queda da oxigenação. Mandatória em atelectasias lobares.^(^ 206 ^)^

**Sugestão -** Pode-se realizar técnicas de expansão pulmonar, na presença de colabamento pulmonar, com redução da complacência e oxigenação.^(^ 207 ^)^

**Recomendação - **Realizar treinamento muscular inspiratório em pacientes com fraqueza muscular inspiratória e VMP para melhorar a força muscular. Seu papel para diminuição do tempo de ventilação mecânica e sucesso de retirada da VM ainda não está estabelecido.^(^ 208 ^)^

Mobilização precoce na ventilação mecânica invasiva e não invasiva

**Recomendação - **A mobilização precoce deve ser iniciada em menos de 72 horas do início da VM, pois é viável, segura e resulta em benefícios funcionais significantes.^(^ 206 ^)^

**Sugestão -** Estimulação elétrica neuromuscular e cicloergômetro podem ser consideradas como complemento do programa de mobilização precoce.^(^ 208 ^)^

**Sugestão -** O treinamento de transferência de sedestação para ortostase pode ser incluído no plano terapêutico e preceder à deambulação, considerando a correlação com a limitação funcional, conforme consenso obtido junto à equipe multiprofissional.^(^ 209 ^)^

**Sugestão -** Pode-se intervir no declínio funcional, visando aumentar as chances de retorno à independência para realizar as Atividades de Vida Diária (AVDs) após a alta hospitalar.^(^ 209 ^)^

## Cuidados em nutrição no paciente sob ventilação mecânica

Determinação das necessidades calóricas

**Sugestão -** Utilizar calorimetria indireta (CI) ou fórmulas preditivas (equações ou fórmula de bolso) para determinar as necessidades calóricas de pacientes graves em VM. A CI deve ser considerada quando disponível, porém é necessário considerar a condição clínica e a frequência da realização. Não existe evidência suficiente para indicar superioridade entre as fórmulas disponíveis na literatura.^(210-214) ^O quadro 11 sugere as fórmulas mais utilizadas na prática diária

**Quadro 11:** Fórmula de bolso e cálculo de Harris Benedict IMC - índice de massa corporal; GEB - gasto energético basal; P - peso; A - altura; I - idade.

**Recomendação - **Iniciar a dieta enteral com uma quantidade menor (20 a 25% da meta) e progredir o volume até alcançá-la em 48 a 72 horas, visando evitar o risco da síndrome da realimentação. Antes de efetuar a evolução, avaliar a tolerabilidade.

Determinação das necessidades proteicas

**Sugestão -** Usar a quantidade de proteína para pacientes em VM de acordo com o IMC,^(^ 210 ^- 215)^ segundo quadro 12.

**Quadro 12:** Quantidade de proteína para pacientes em ventilação mecânica de acordo com índice de massa corporal

**Sugestão -** Individualizar a necessidade proteica para pacientes graves em VM com disfunção renal aguda. Um aspecto importante a considerar é que esses pacientes não deveriam receber dieta restrita em proteína como meio de evitar ou retardar a terapia de substituição renal (TSR). Considerar que pacientes em TSR apresentam perda considerável de 10 a 15g de aminoácidos durante a sessão de diálise.^(210-216) ^Em pacientes não candidatos à diálise, podem-se utilizar dietas especiais para pacientes nefropatas.

Vias de administração 

**Recomendação - **Utilizar a via enteral como primeira opção, sempre que houver viabilidade do trato gastrintestinal.^(^ 211 ^,^ 217 ^)^

**Sugestão -** Evitar iniciar nutrição parenteral (NP) em pacientes graves em VM até que todas as estratégias para otimizar a nutrição enteral (NE) sejam esgotadas. 

Enteral precoce

**Recomendação - **Iniciar a nutrição enteral precoce (dentro de 24 a 48 horas da admissão na UTI), desde que o paciente esteja hemodinamicamente estável. A terapia nutricional (TN) enteral precoce demonstrou redução da taxa de mortalidade em pacientes graves em VM e, além disso, foi associada com uma redução nas complicações infecciosas e no tempo permanência hospitalar.^(^ 208 ^-^ 211 ^,^ 217 ^,^ 218 ^)^

Estratégia para otimizar o fornecimento da nutrição enteral e minimizar os riscos em pacientes sob ventilação mecânica^(^ 219 ^)^

Cabeceira elevada

**Recomendação - **Deve-se manter a cabeceira entre 30^o^ e 45^o^, salvo contra indicação, para todos os paciente intubados recebendo a nutrição enteral.^(^ 200 ^,^ 210 ^,^ 220 ^)^

Posicionamento da sonda para nutrição 

**Recomendação - **Devem-se considerar as duas vias (gástrica e/ou pós-pilórica) para pacientes em VM, reservando o posicionamento pós-pilórico para pacientes com intolerância e/ou contraindicação gástrica.^(^ 200 ^,^ 211 ^)^

**Sugestão -** Considerar gastrostomia ou jejunostomia para pacientes em VM, requerendo nutrição enteral <4 semanas, de acordo com a clínica do paciente.^(^ 221 ^)^

Monitoramento do resíduo gástrico em pacientes sob ventilação mecânica

**Recomendação - **Não utilizar o monitoramento do volume de resíduo gástrico (RG) em paciente mecanicamente ventilados com o objetivo de prevenir PAV.^(222,223) ^Como efeito positivo, oferecer NE precoce sem o monitoramento do RG nos pacientes em VM melhorou o fornecimento da nutrição enteral.

Nutrição enteral contínua comparada a outros métodos

**Sugestão -** Pode-se utilizar o método contínuo em bomba de infusão^(^ 210 ^)^ para aqueles pacientes graves em VM com intolerância à TNE.

Estabelecimento de protocolo de terapia nutricional

**Sugestão -** Podem-se implementar diretrizes no serviço para otimizar a TN enteral em pacientes sob VM, com intuito de amenizar o défice calórico proteico.^(^ 224 ^)^

**Sugestão -** Podem-se utilizar procinéticos (preferir metoclopramida) para melhorar a tolerância, visando alcançar a meta calórica enteral.^(^ 210 ^)^

Cuidados específicos

Dieta rica em lipídio e pobre em carboidrato

**Sugestão -** Podem-se utilizar formulações projetadas para manipular o quociente respiratório e reduzir a produção de CO_2_ (rica em lipídeos e baixo carboidrato) em pacientes selecionados (pacientes DPOC com retenção de CO_2_, com SDRA grave e hipercapnia permissiva e ventilação protetora limítrofe, naqueles apresentando desmame difícil ou prolongado com retenção de CO_2_). Esforços devem ser realizados para evitar o excesso de caloria total.^(^ 200 ^,^ 210 ^,^ 225 ^)^

Dieta enteral enriquecida com óleo de peixe, óleo de borragem, vitaminas antioxidantes

**Sugestão -** Pode-se utilizar formulações enterais com perfil lipídico anti-inflamatório e com antioxidantes para pacientes em VM com SDRA.^(200,211,226-229) ^Doses elevadas de ômega 3 devem ser evitadas para aqueles pacientes que apresentam distúrbios de coagulação.^(^ 200 ^,^ 211 ^,^ 226 ^-^ 229 ^)^

Reposição de fósforo

**Sugestão -** Pode-se repor o défice de fósforo em pacientes sob VM. Essa consideração se justifica pela associação entre hipofosfatemia e falência da retirada VM.^(^ 230 ^)^
